# Supplementary figures and images for: Septins function in exocytosis via physical interactions with the exocyst complex in fission yeast cytokinesis
Source: eLife. 2025 Oct 31;13:RP101113. doi: 10.7554/eLife.101113 (PMC12578440; doi:10.7554/eLife.101113)

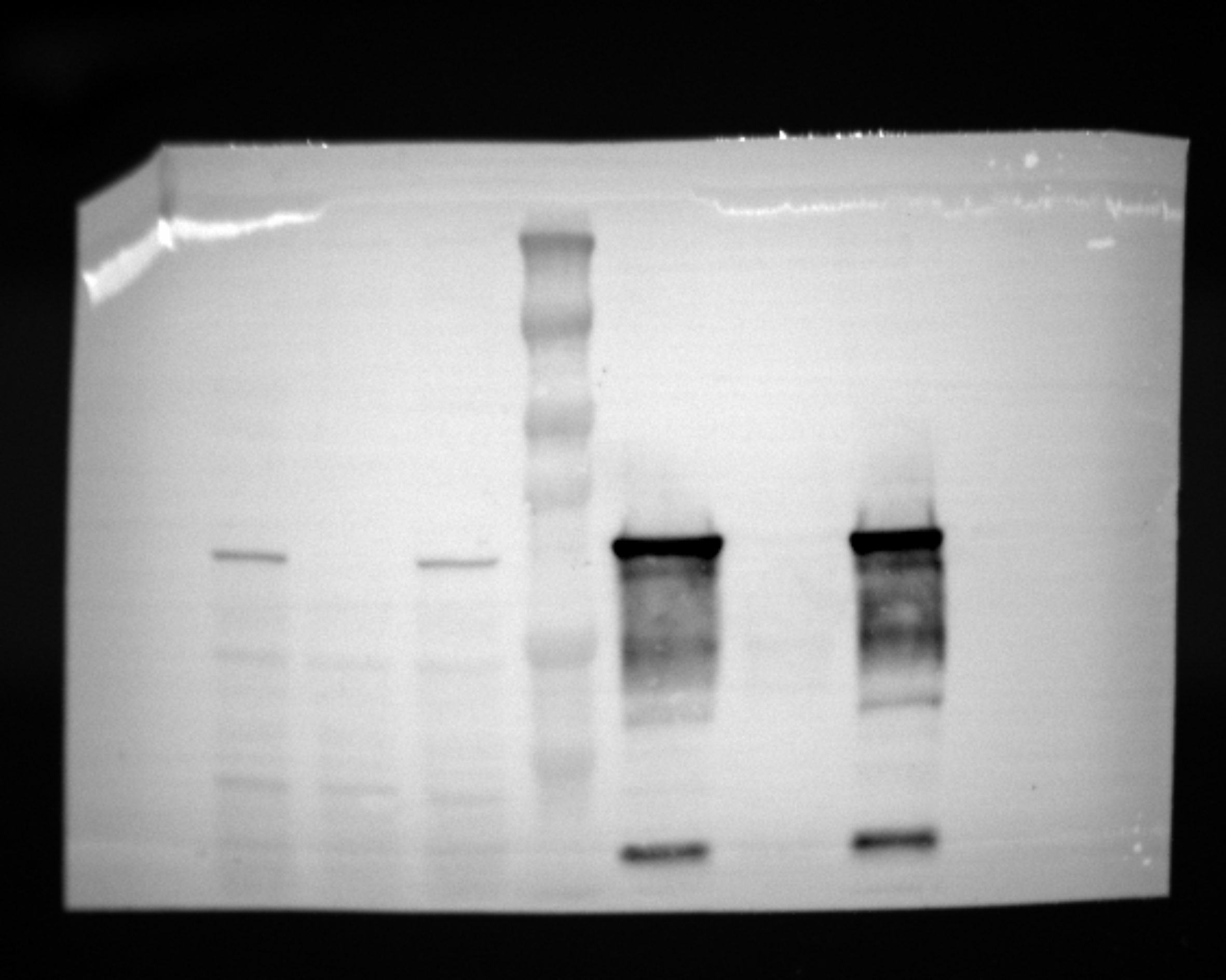

Supplement: Figure 4—source data 1. [file elife-101113-fig4-data1.zip › Figure 4-source data 1/Figure 4 Panel A GFP.tif]

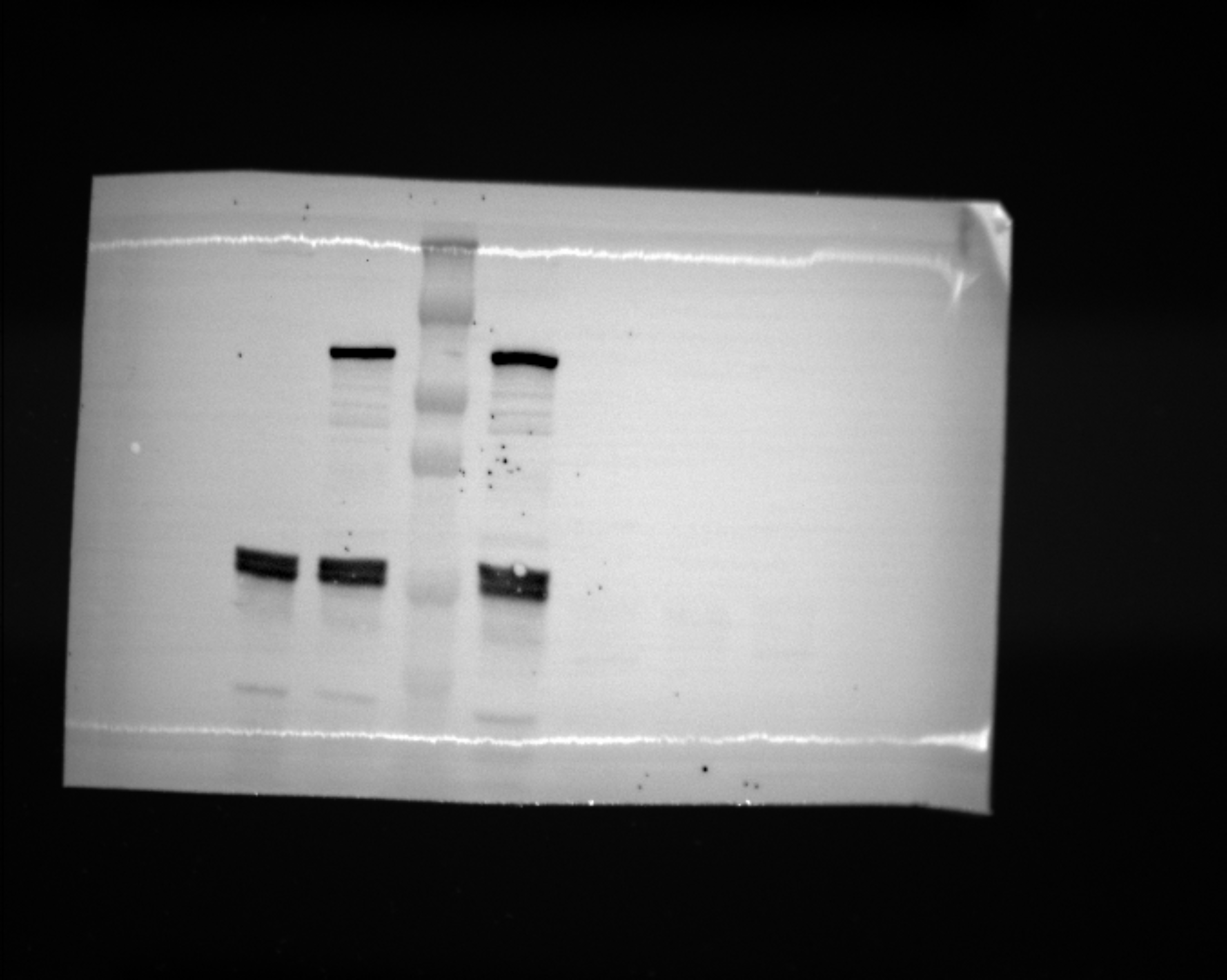

Supplement: Figure 4—source data 1. [file elife-101113-fig4-data1.zip › Figure 4-source data 1/Figure 4 Panel A Myc Tubulin.tif]

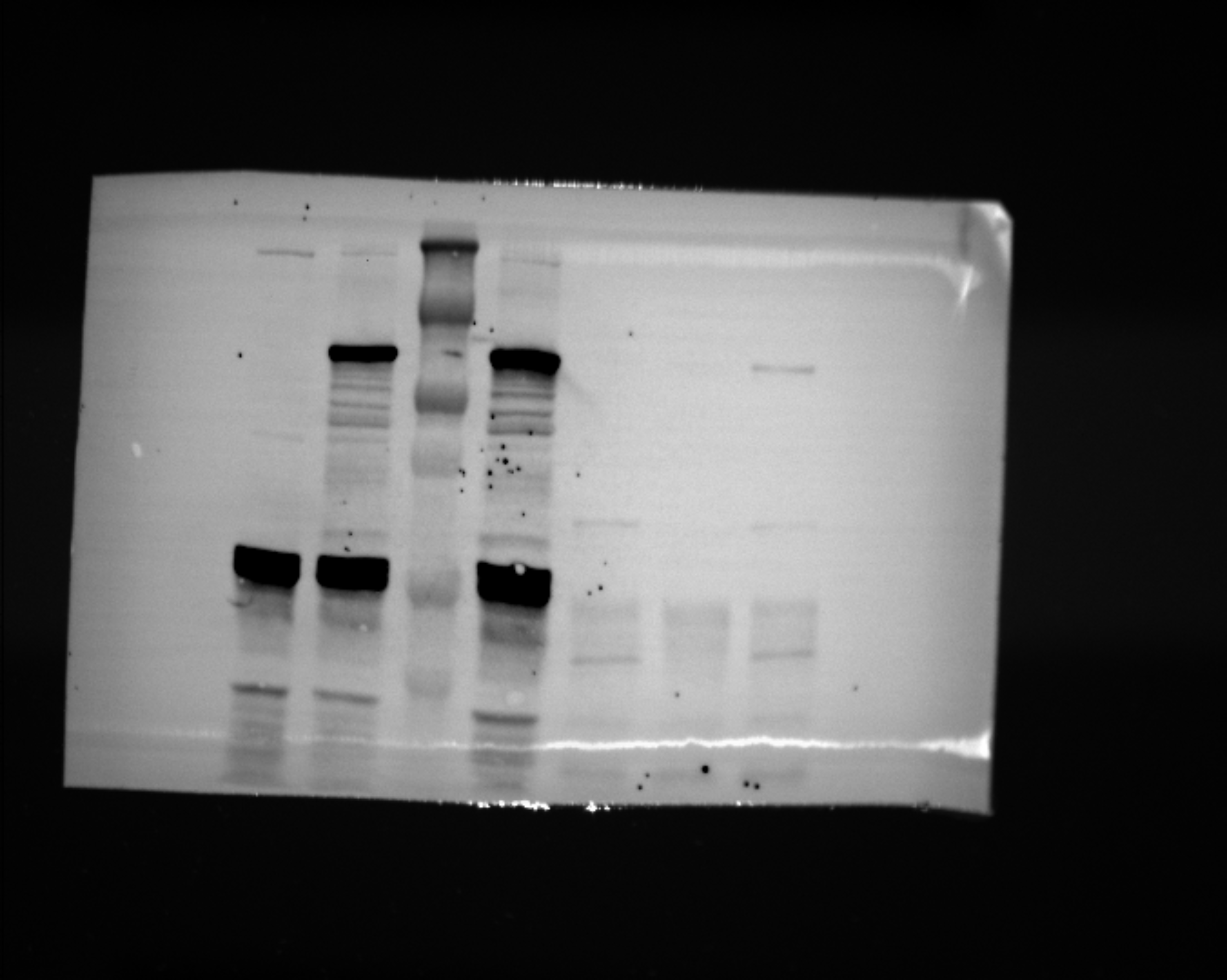

Supplement: Figure 4—source data 1. [file elife-101113-fig4-data1.zip › Figure 4-source data 1/Figure 4 Panel A Myc.tif]

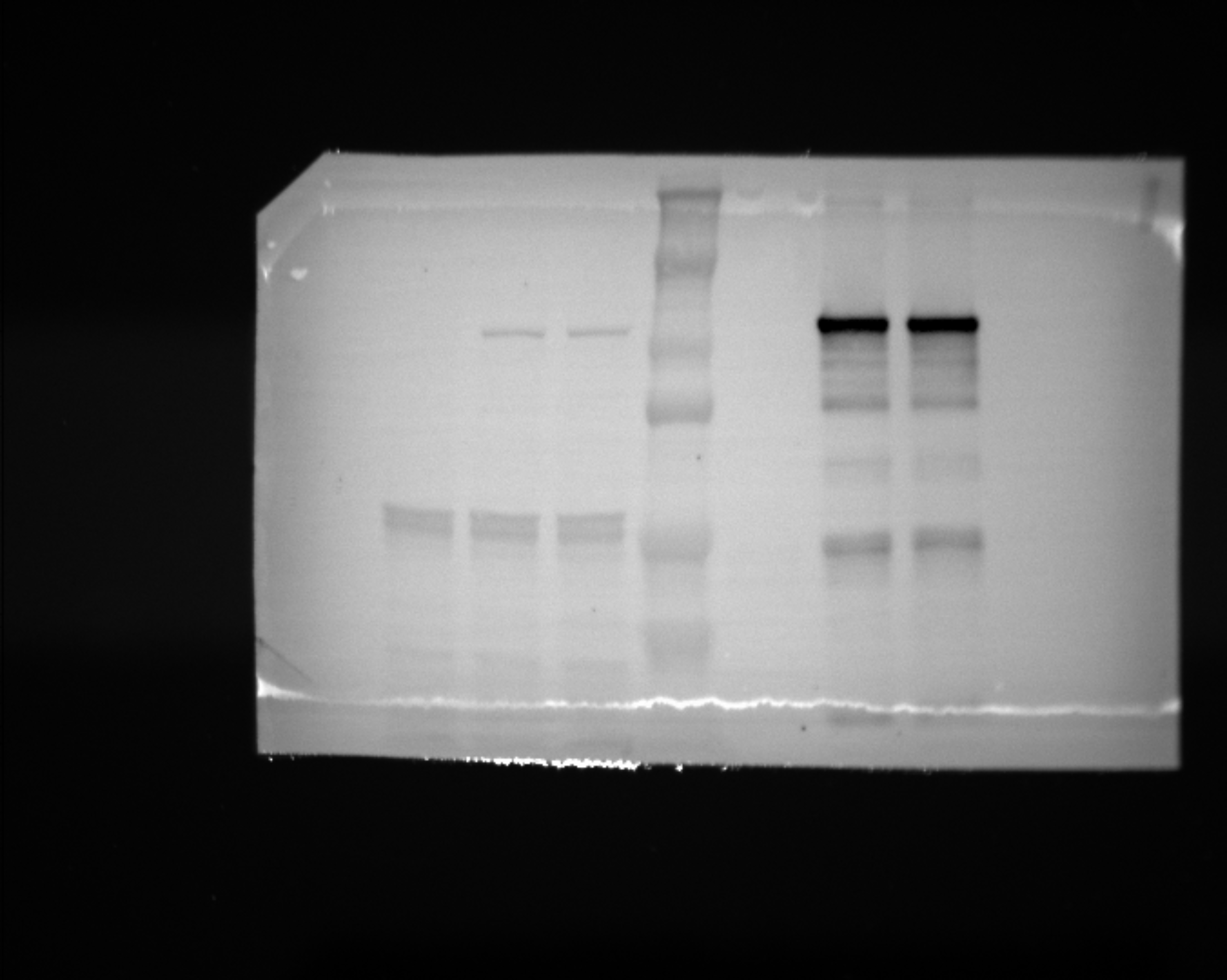

Supplement: Figure 4—source data 1. [file elife-101113-fig4-data1.zip › Figure 4-source data 1/Figure 4 Panel B GFP.tif]

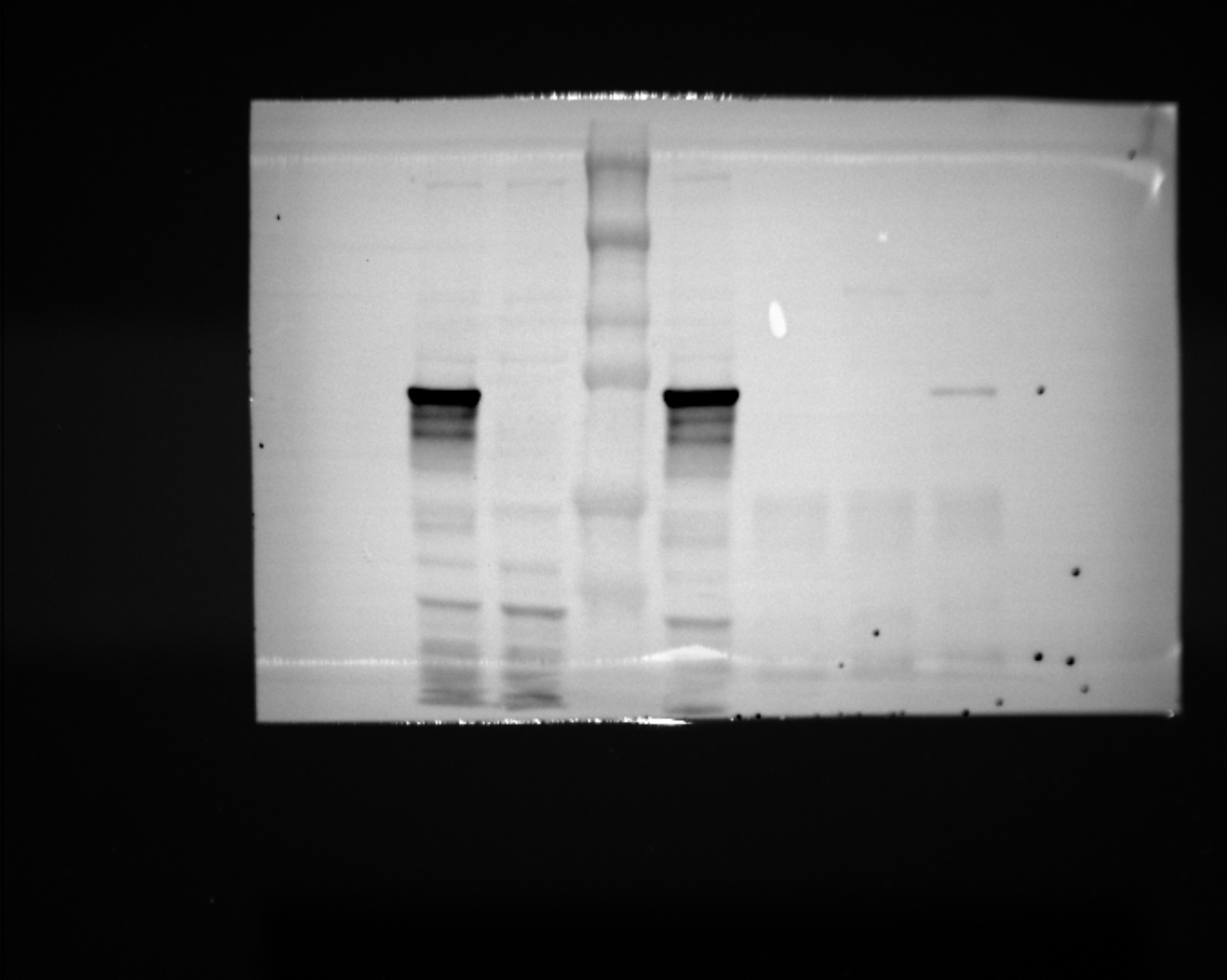

Supplement: Figure 4—source data 1. [file elife-101113-fig4-data1.zip › Figure 4-source data 1/Figure 4 Panel B Myc Tubulin.tif]

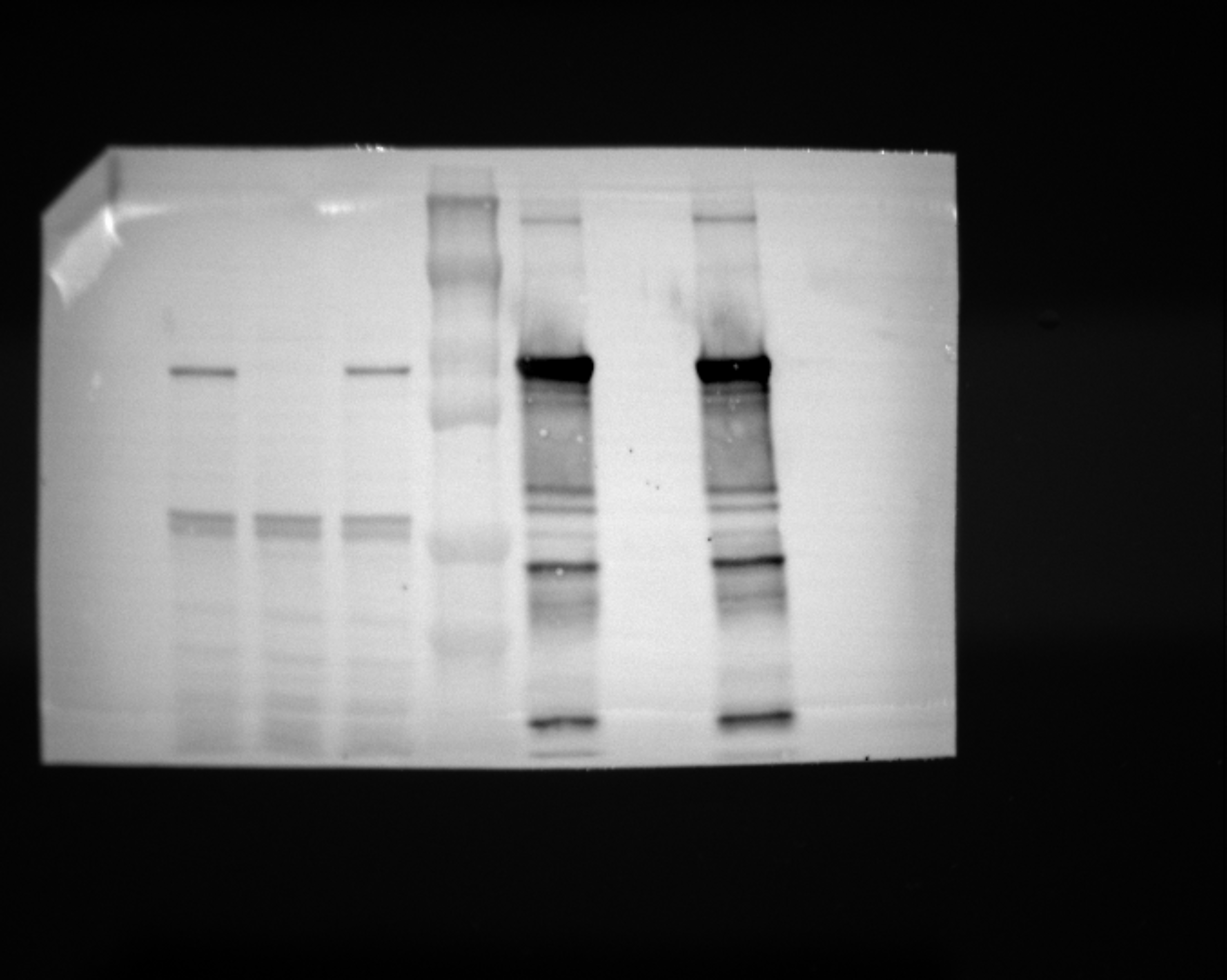

Supplement: Figure 4—source data 1. [file elife-101113-fig4-data1.zip › Figure 4-source data 1/Figure 4 Panel C GFP.tif]

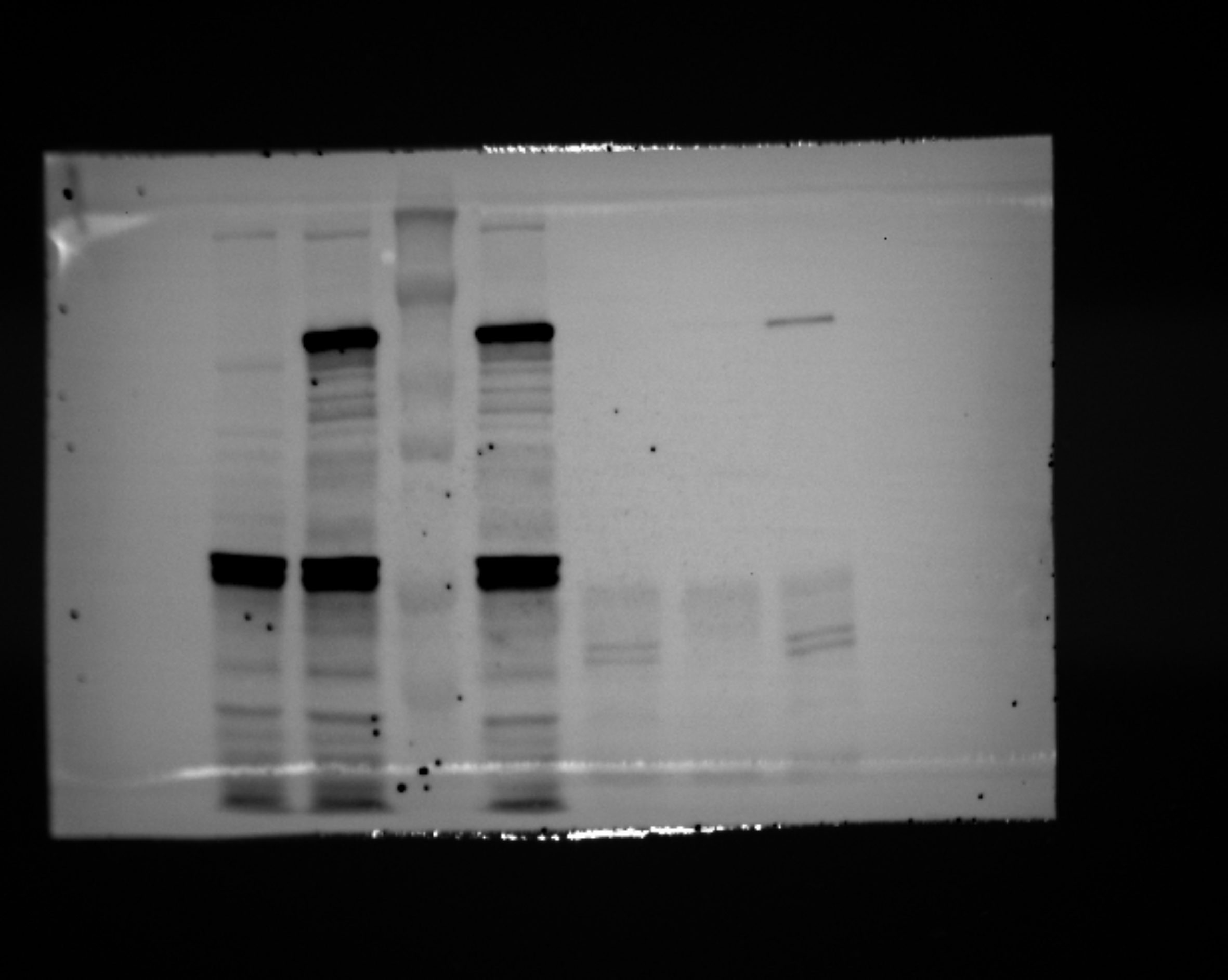

Supplement: Figure 4—source data 1. [file elife-101113-fig4-data1.zip › Figure 4-source data 1/Figure 4 Panel C Myc Tubulin.tif]

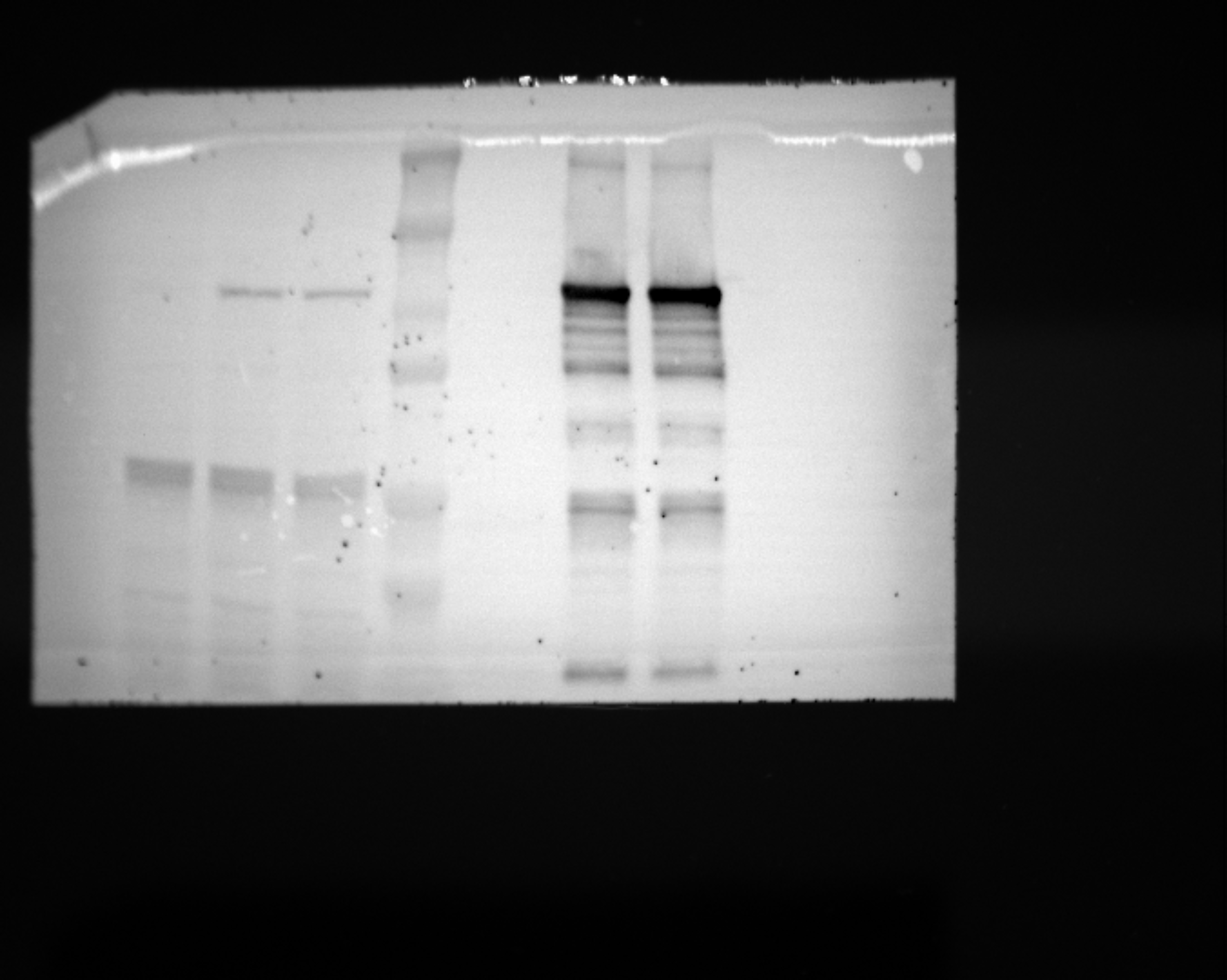

Supplement: Figure 4—source data 1. [file elife-101113-fig4-data1.zip › Figure 4-source data 1/Figure 4 Panel D GFP.tif]

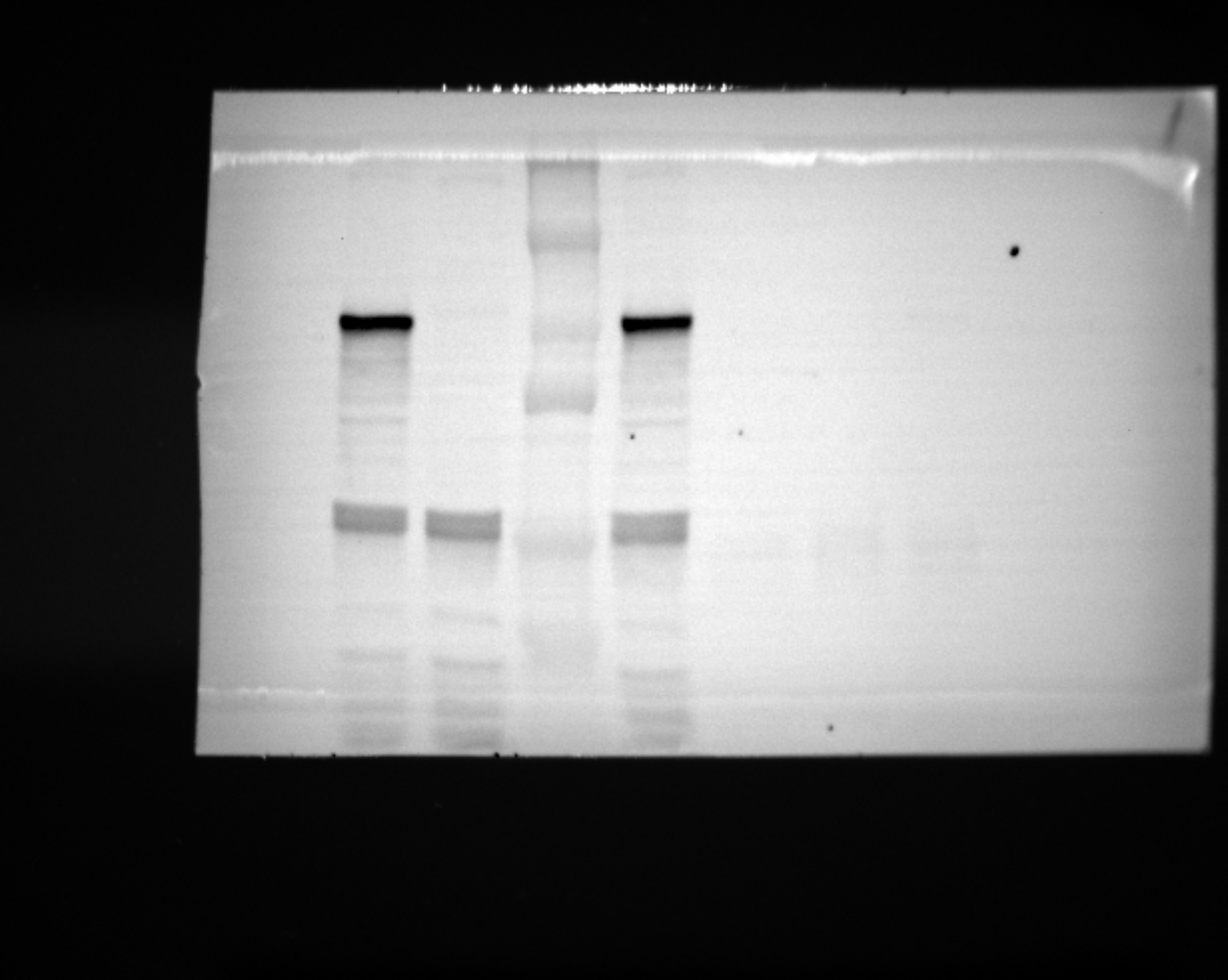

Supplement: Figure 4—source data 1. [file elife-101113-fig4-data1.zip › Figure 4-source data 1/Figure 4 Panel D Myc Tubulin.tif]

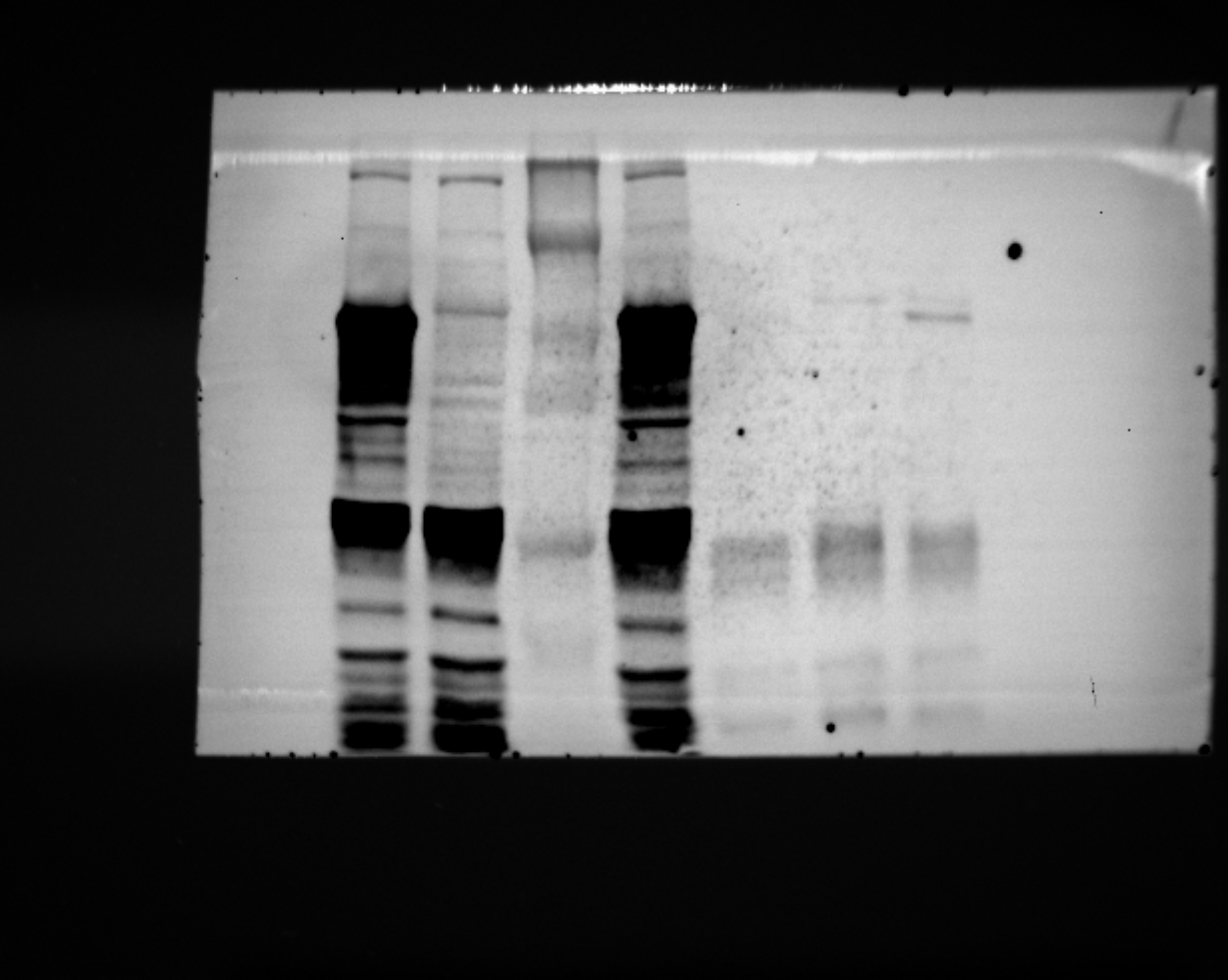

Supplement: Figure 4—source data 1. [file elife-101113-fig4-data1.zip › Figure 4-source data 1/Figure 4 Panel D Myc.tif]

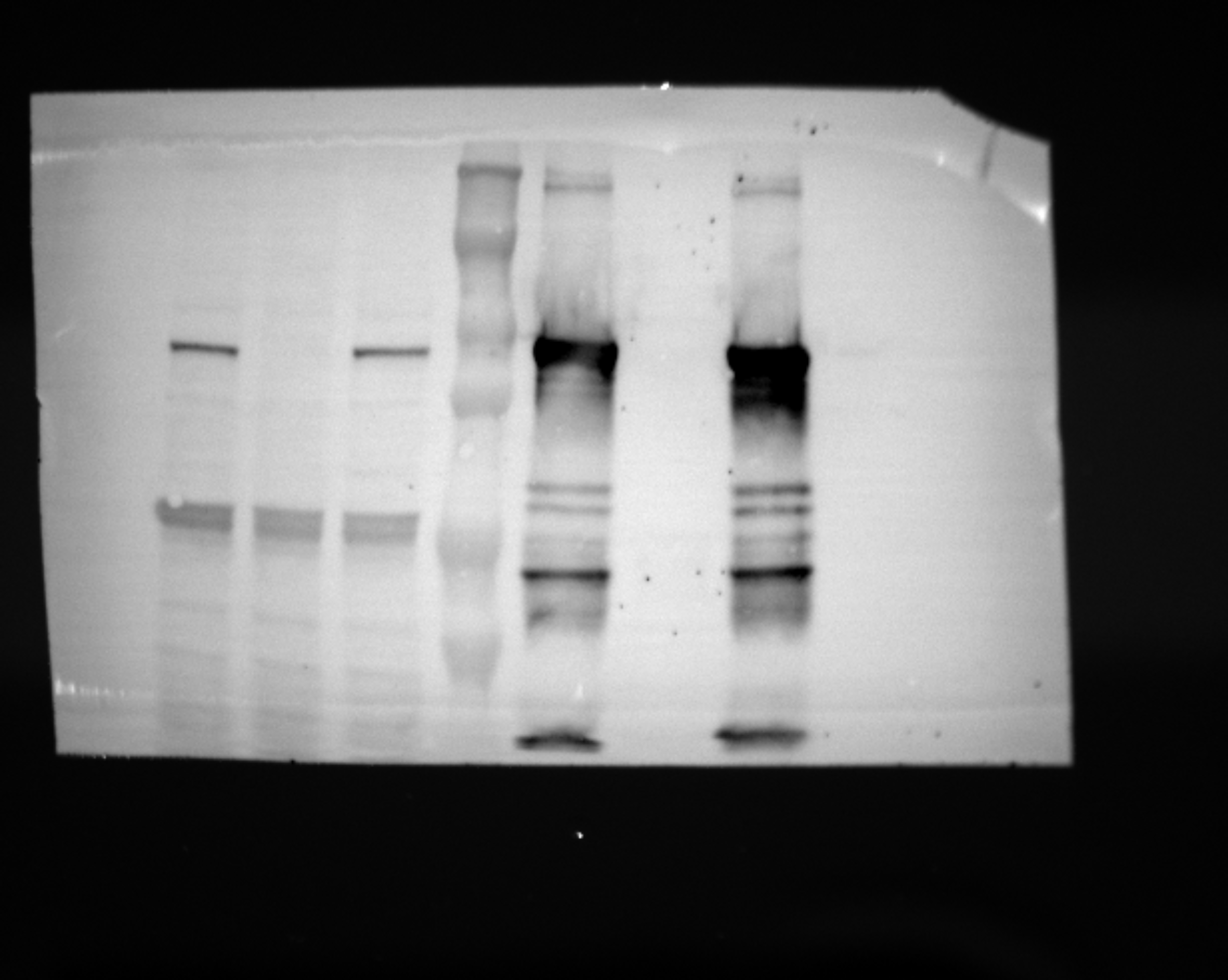

Supplement: Figure 4—figure supplement 1—source data 1. [file elife-101113-fig4-figsupp1-data1.zip › Figure 4-figure supplement 1-source data 1/Figure 4-figure supplement 1 Panel A GFP Tubulin.tif]

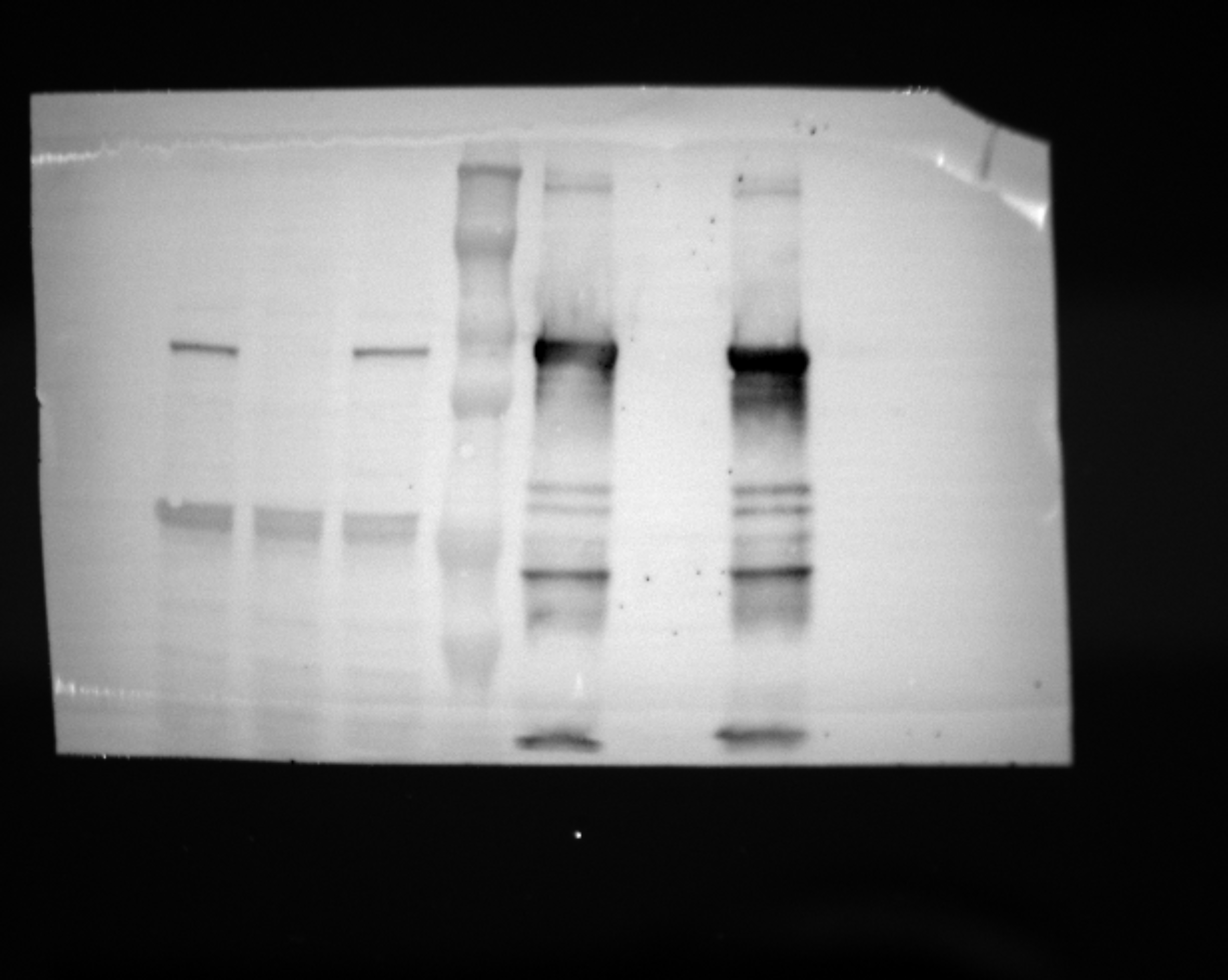

Supplement: Figure 4—figure supplement 1—source data 1. [file elife-101113-fig4-figsupp1-data1.zip › Figure 4-figure supplement 1-source data 1/Figure 4-figure supplement 1 Panel A GFP.tif]

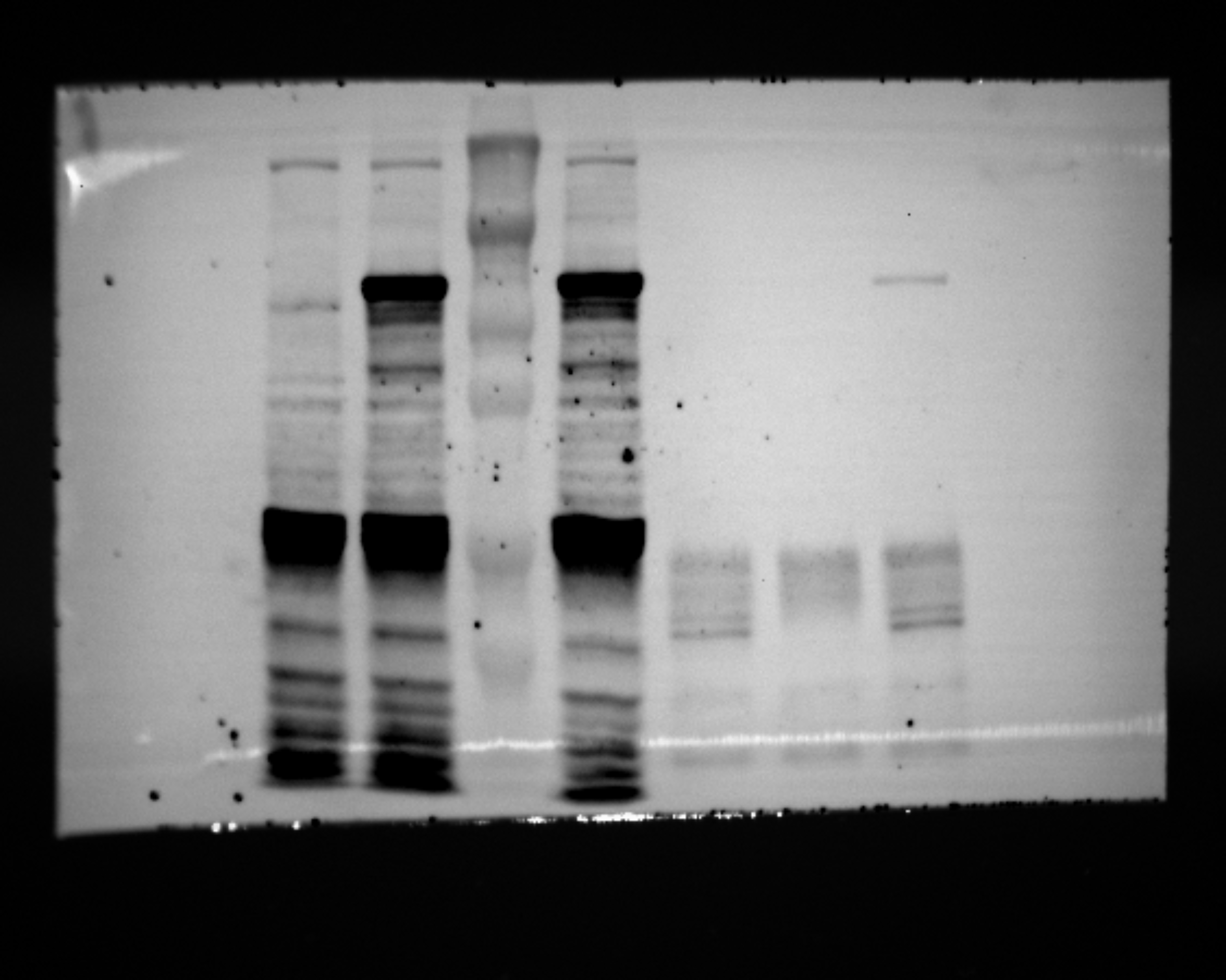

Supplement: Figure 4—figure supplement 1—source data 1. [file elife-101113-fig4-figsupp1-data1.zip › Figure 4-figure supplement 1-source data 1/Figure 4-figure supplement 1 Panel A Myc.tif]

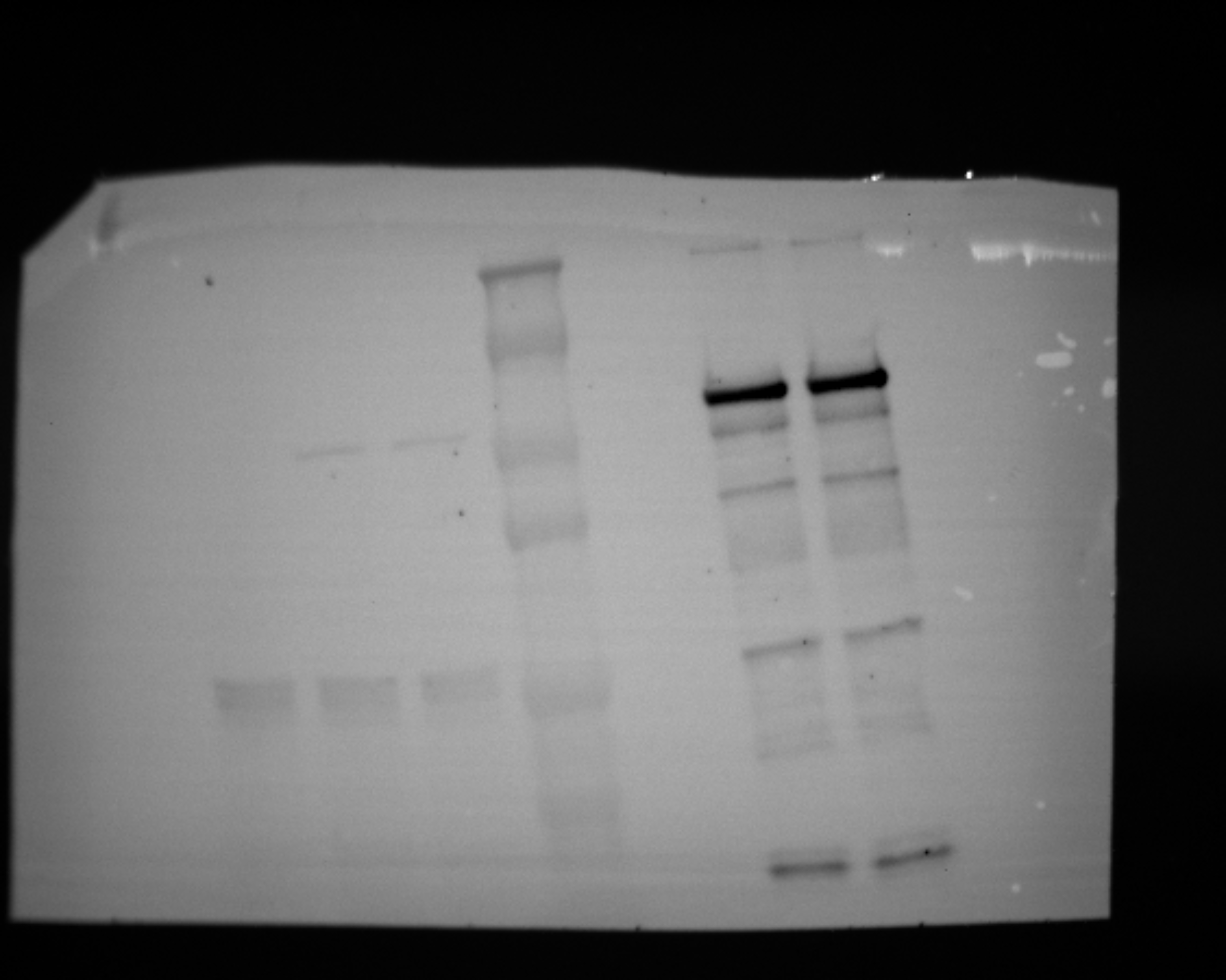

Supplement: Figure 4—figure supplement 1—source data 1. [file elife-101113-fig4-figsupp1-data1.zip › Figure 4-figure supplement 1-source data 1/Figure 4-figure supplement 1 Panel B GFP.tif]

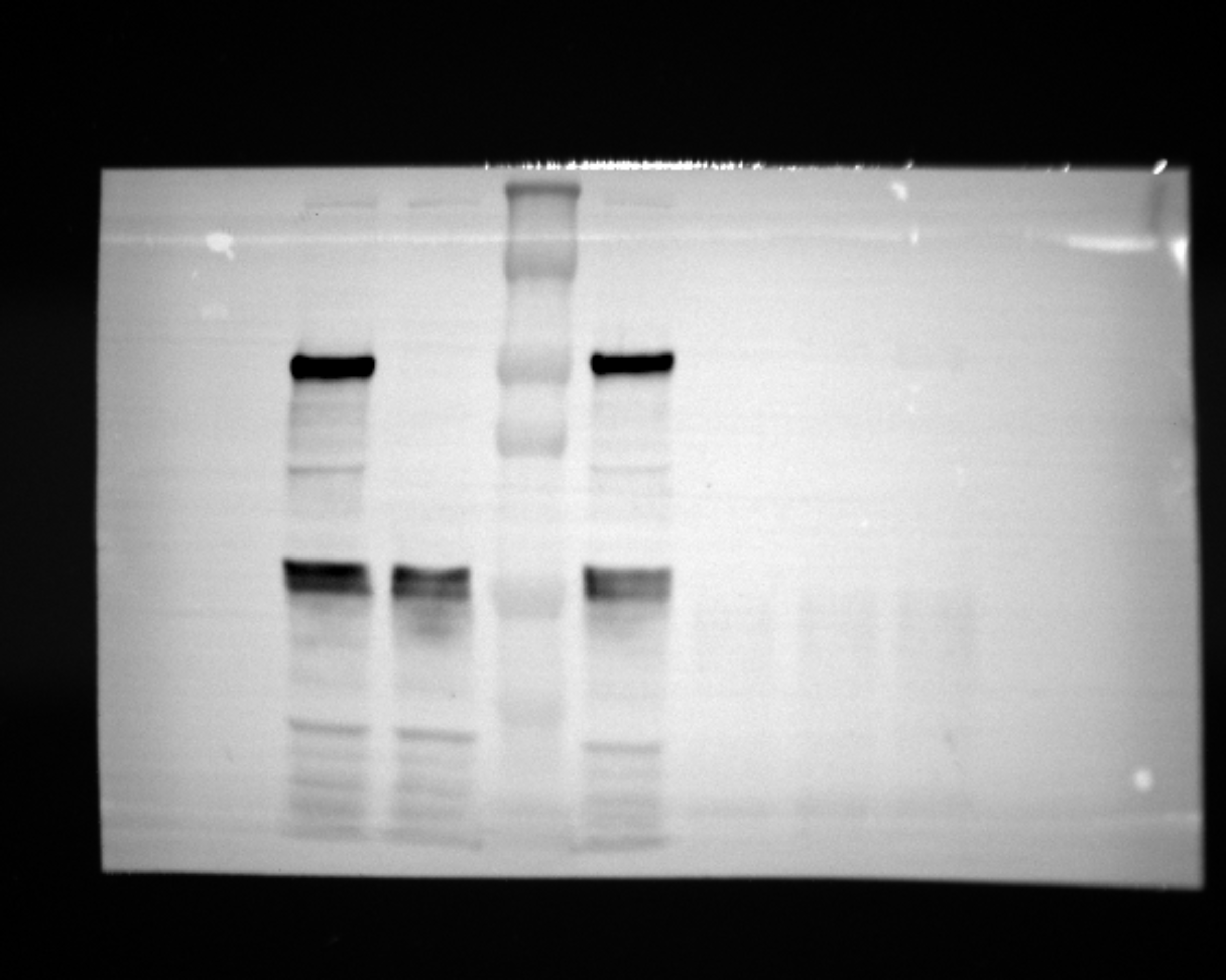

Supplement: Figure 4—figure supplement 1—source data 1. [file elife-101113-fig4-figsupp1-data1.zip › Figure 4-figure supplement 1-source data 1/Figure 4-figure supplement 1 Panel B Myc Tubulin.tif]

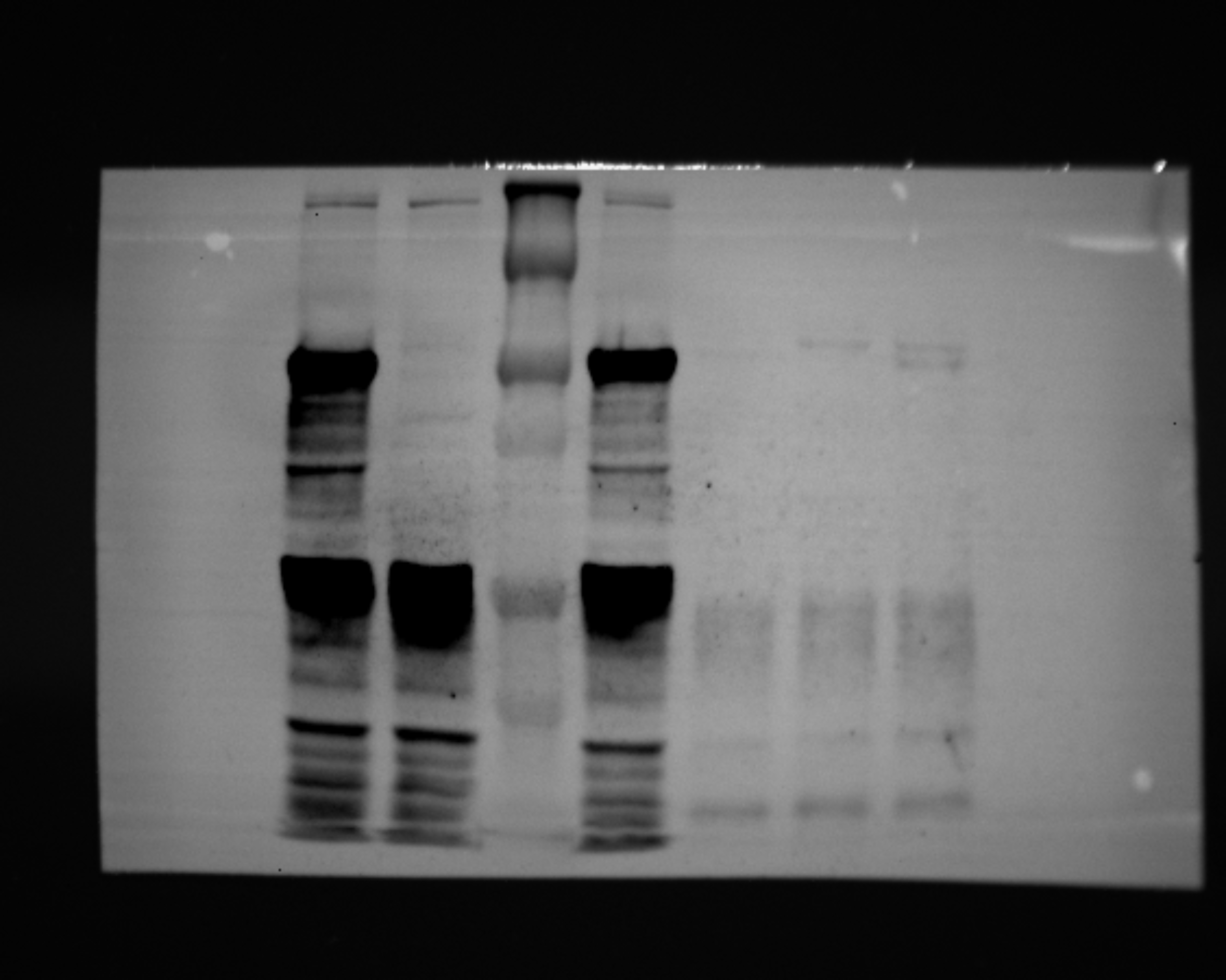

Supplement: Figure 4—figure supplement 1—source data 1. [file elife-101113-fig4-figsupp1-data1.zip › Figure 4-figure supplement 1-source data 1/Figure 4-figure supplement 1 Panel B Myc.tif]

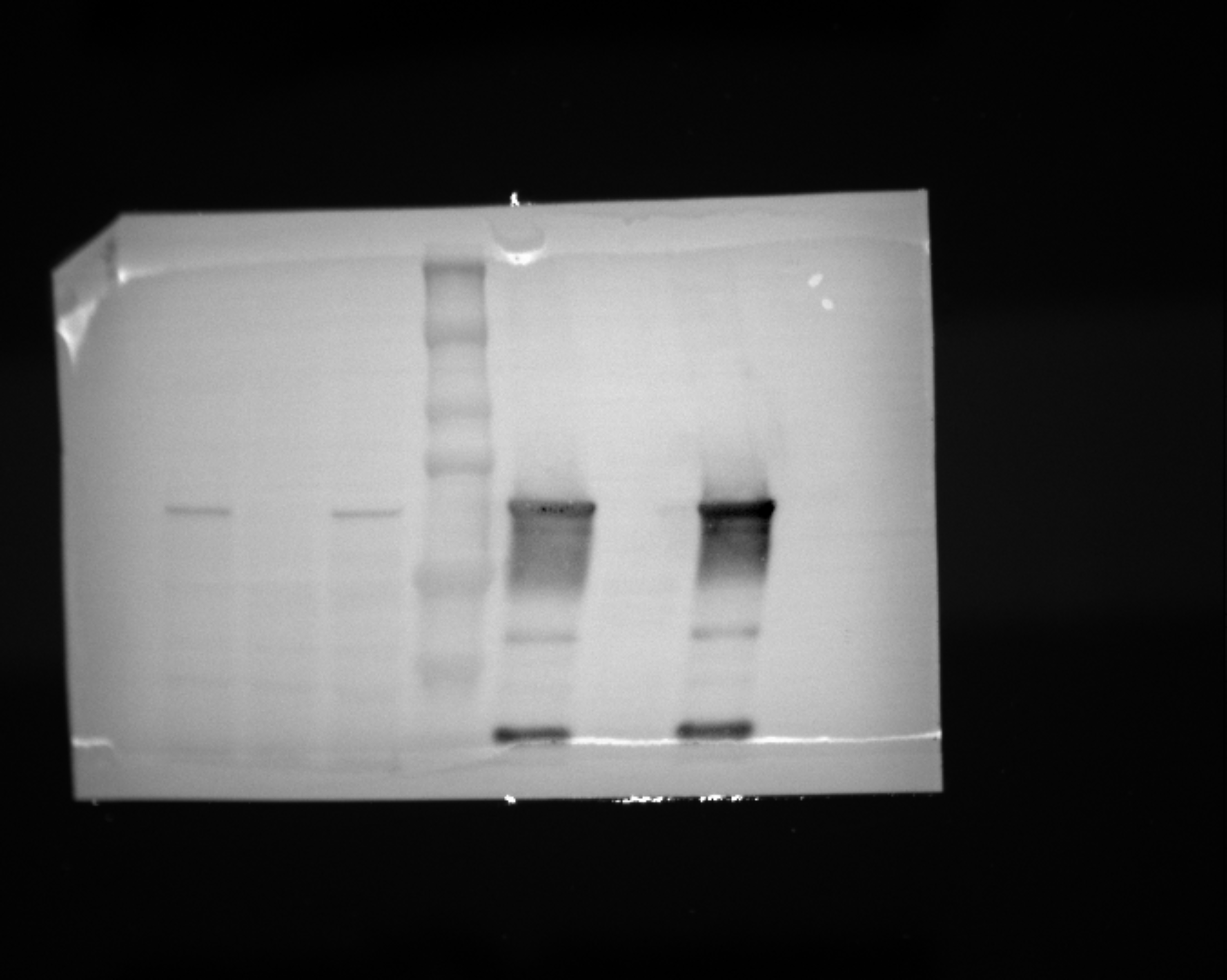

Supplement: Figure 4—figure supplement 1—source data 1. [file elife-101113-fig4-figsupp1-data1.zip › Figure 4-figure supplement 1-source data 1/Figure 4-figure supplement 1 Panel C GFP.tif]

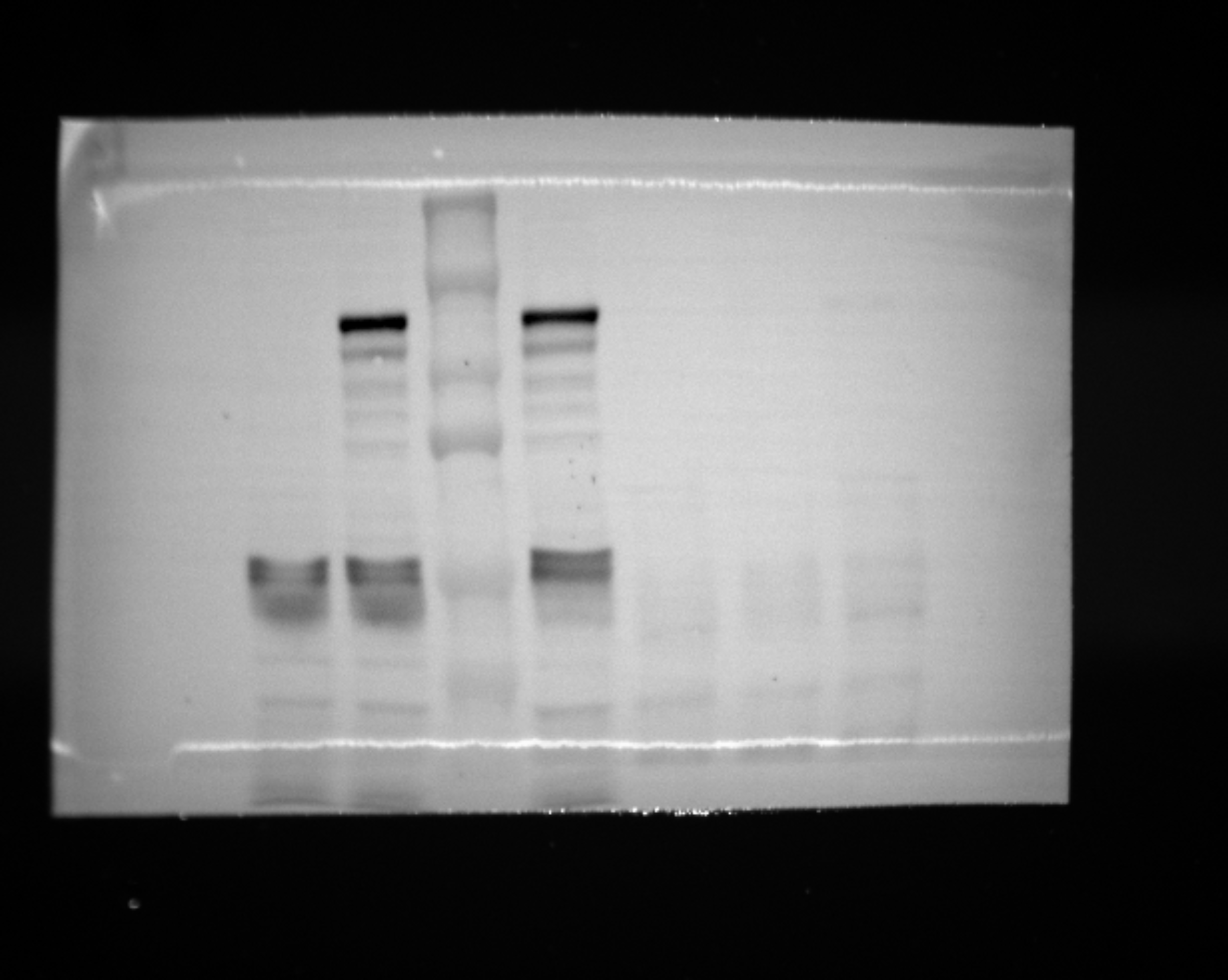

Supplement: Figure 4—figure supplement 1—source data 1. [file elife-101113-fig4-figsupp1-data1.zip › Figure 4-figure supplement 1-source data 1/Figure 4-figure supplement 1 Panel C Myc Tubulin.tif]

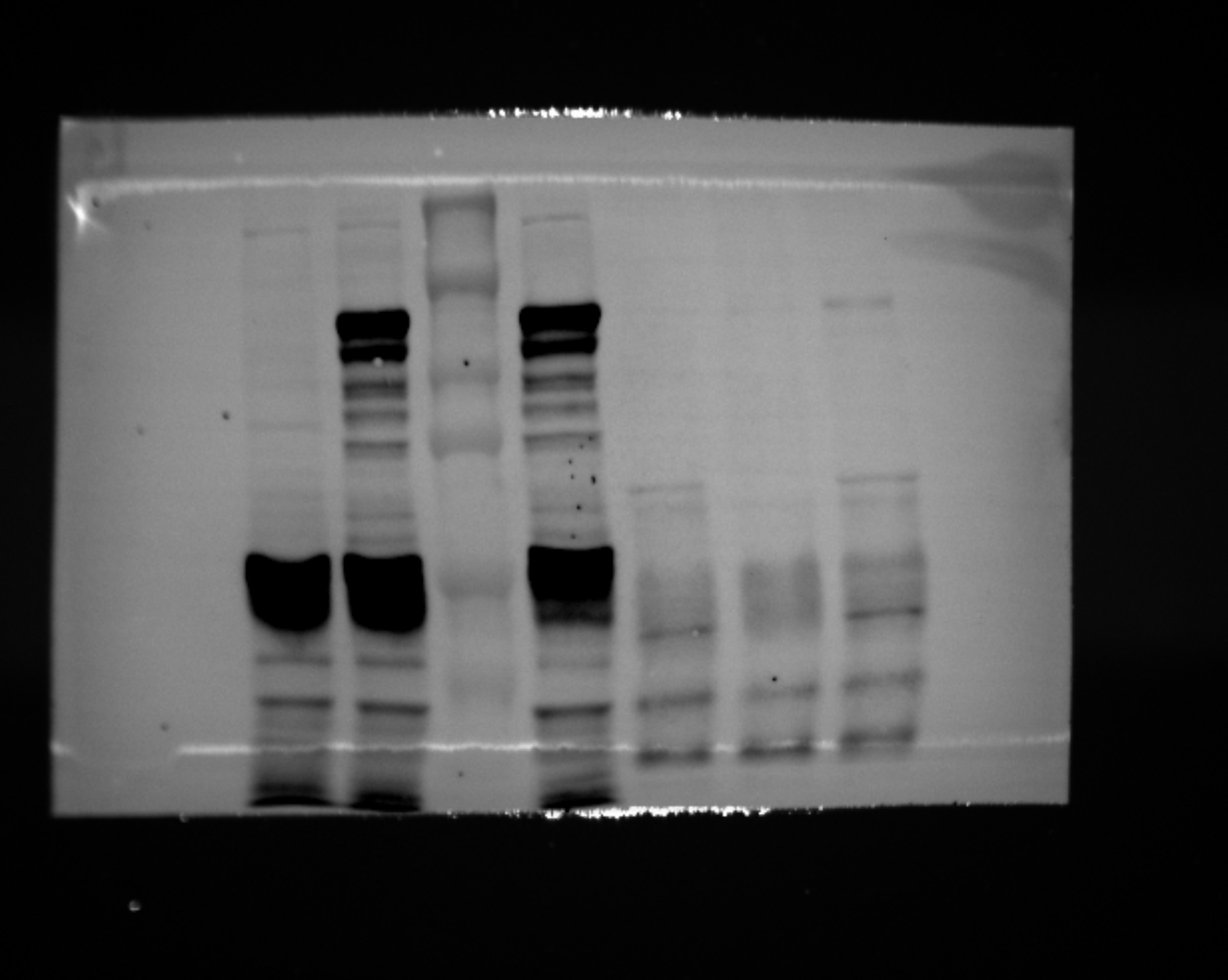

Supplement: Figure 4—figure supplement 1—source data 1. [file elife-101113-fig4-figsupp1-data1.zip › Figure 4-figure supplement 1-source data 1/Figure 4-figure supplement 1 Panel C Myc.tif]

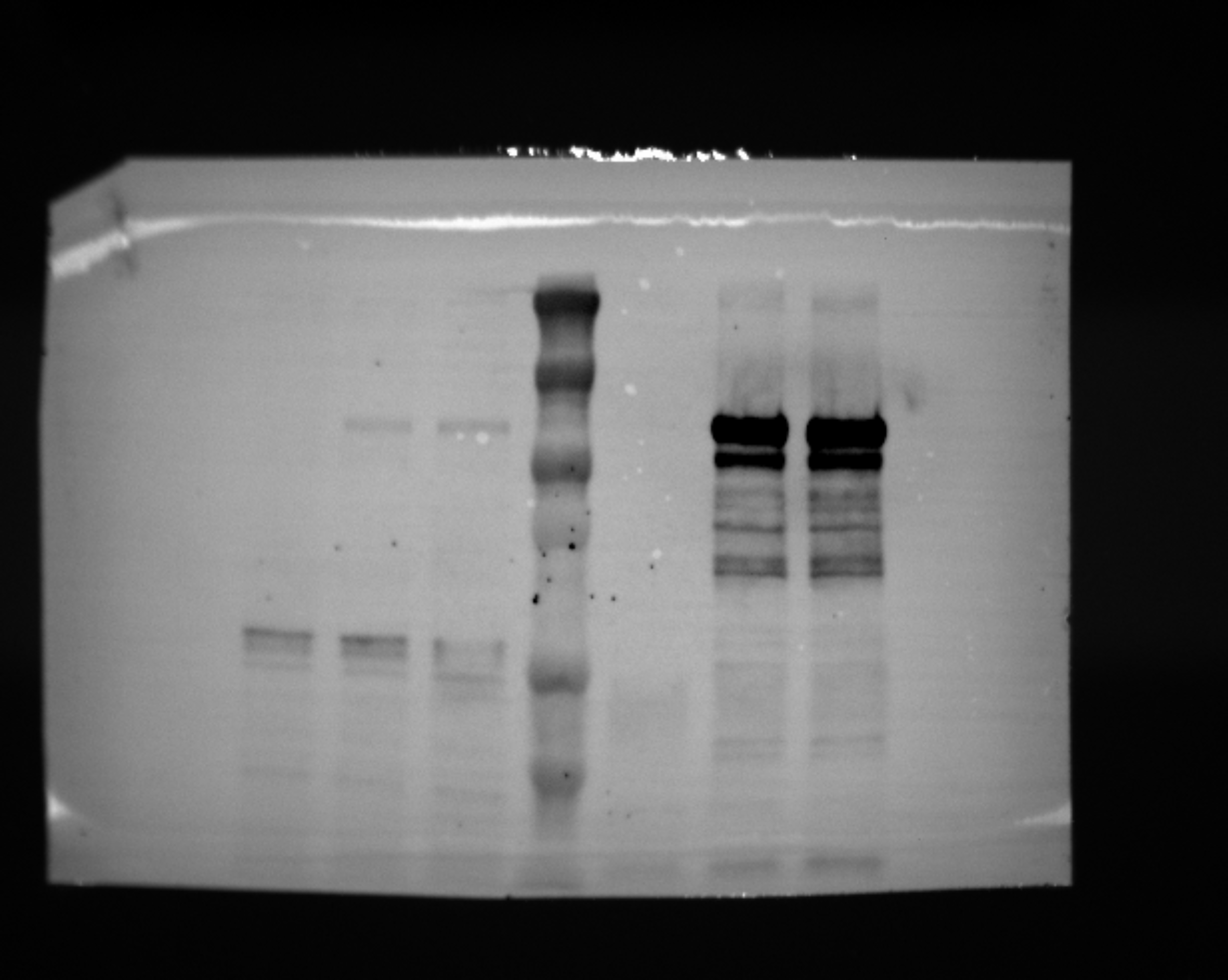

Supplement: Figure 4—figure supplement 1—source data 1. [file elife-101113-fig4-figsupp1-data1.zip › Figure 4-figure supplement 1-source data 1/Figure 4-figure supplement 1 Panel D GFP.tif]

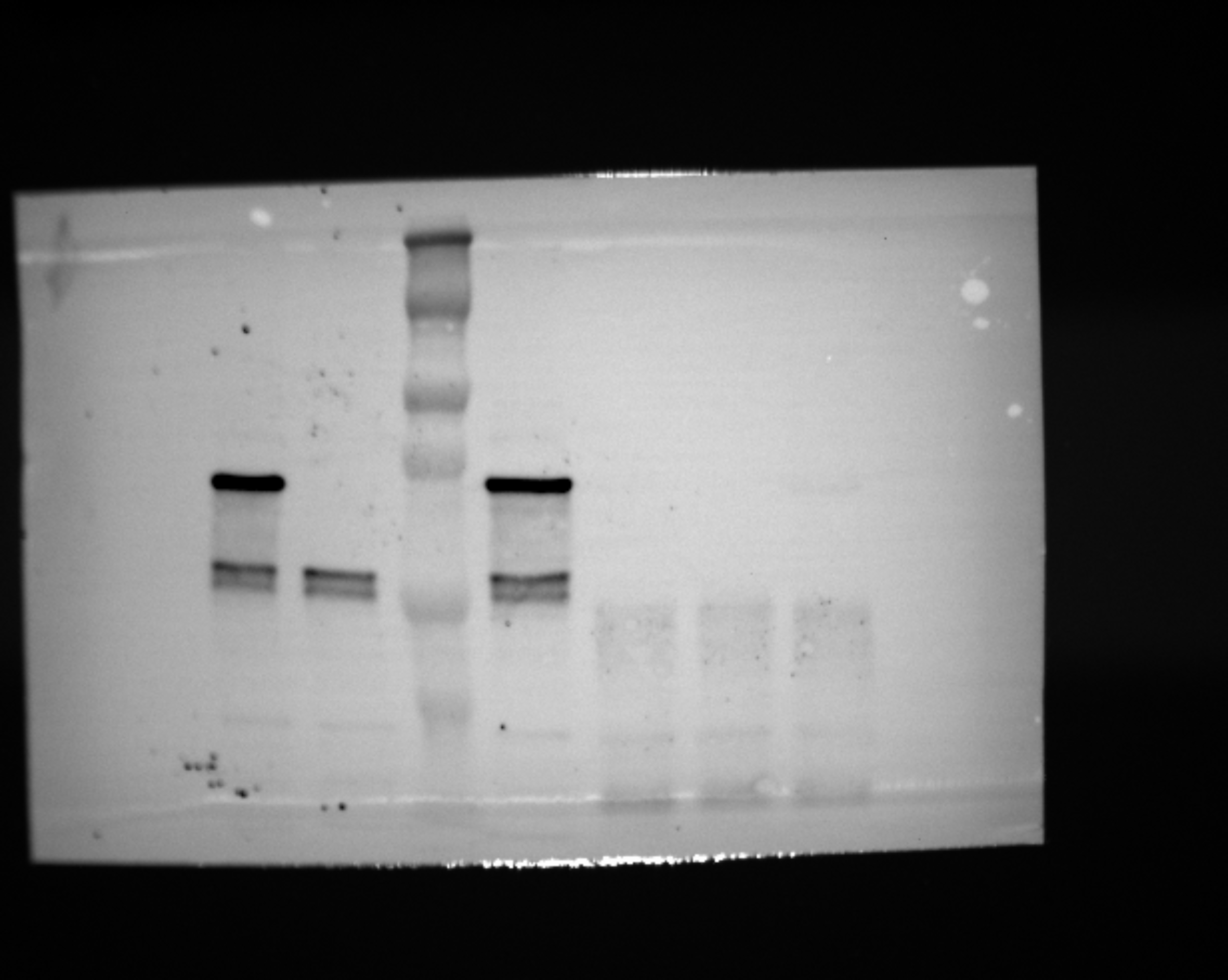

Supplement: Figure 4—figure supplement 1—source data 1. [file elife-101113-fig4-figsupp1-data1.zip › Figure 4-figure supplement 1-source data 1/Figure 4-figure supplement 1 Panel D Myc Tubulin.tif]

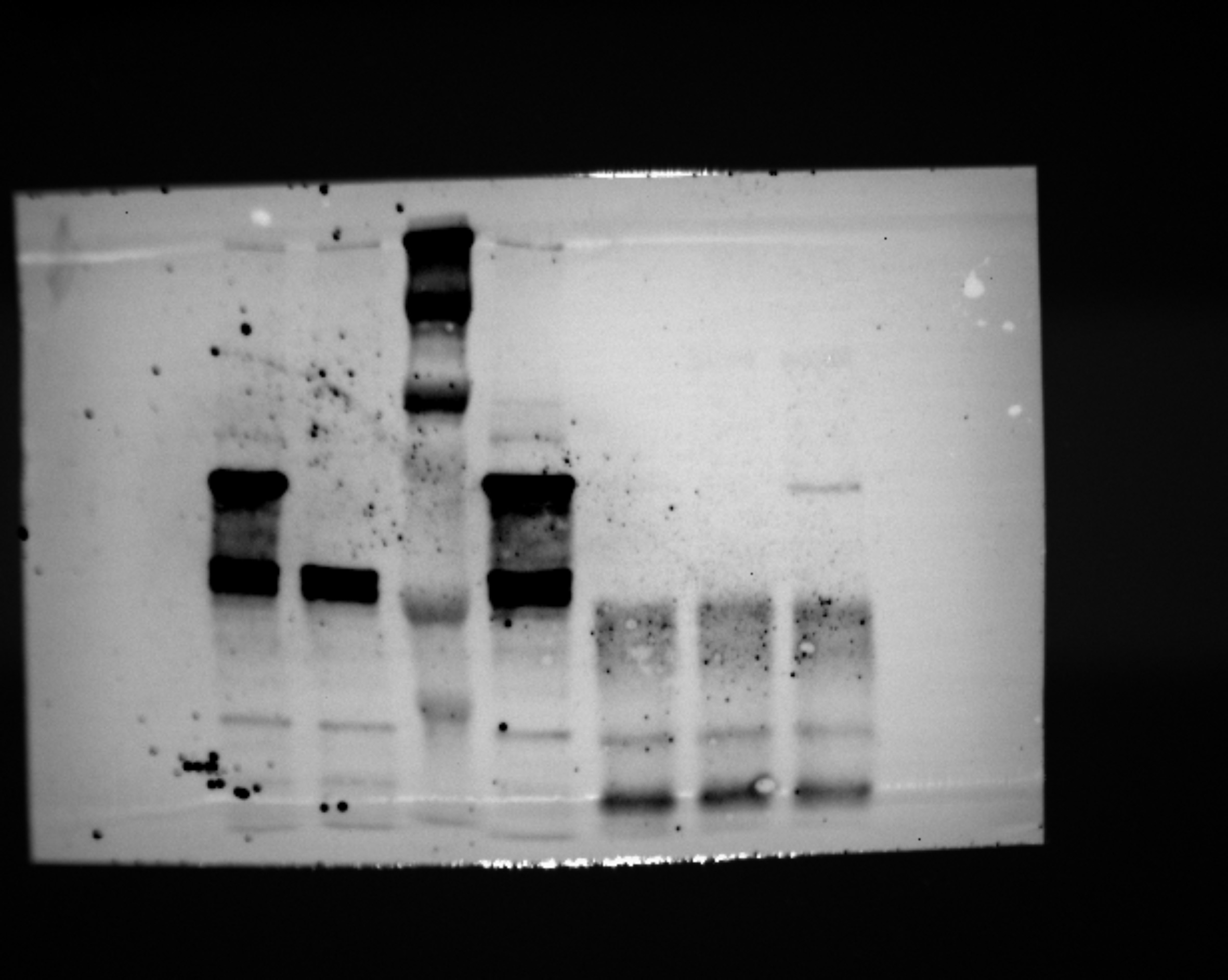

Supplement: Figure 4—figure supplement 1—source data 1. [file elife-101113-fig4-figsupp1-data1.zip › Figure 4-figure supplement 1-source data 1/Figure 4-figure supplement 1 Panel D Myc.tif]

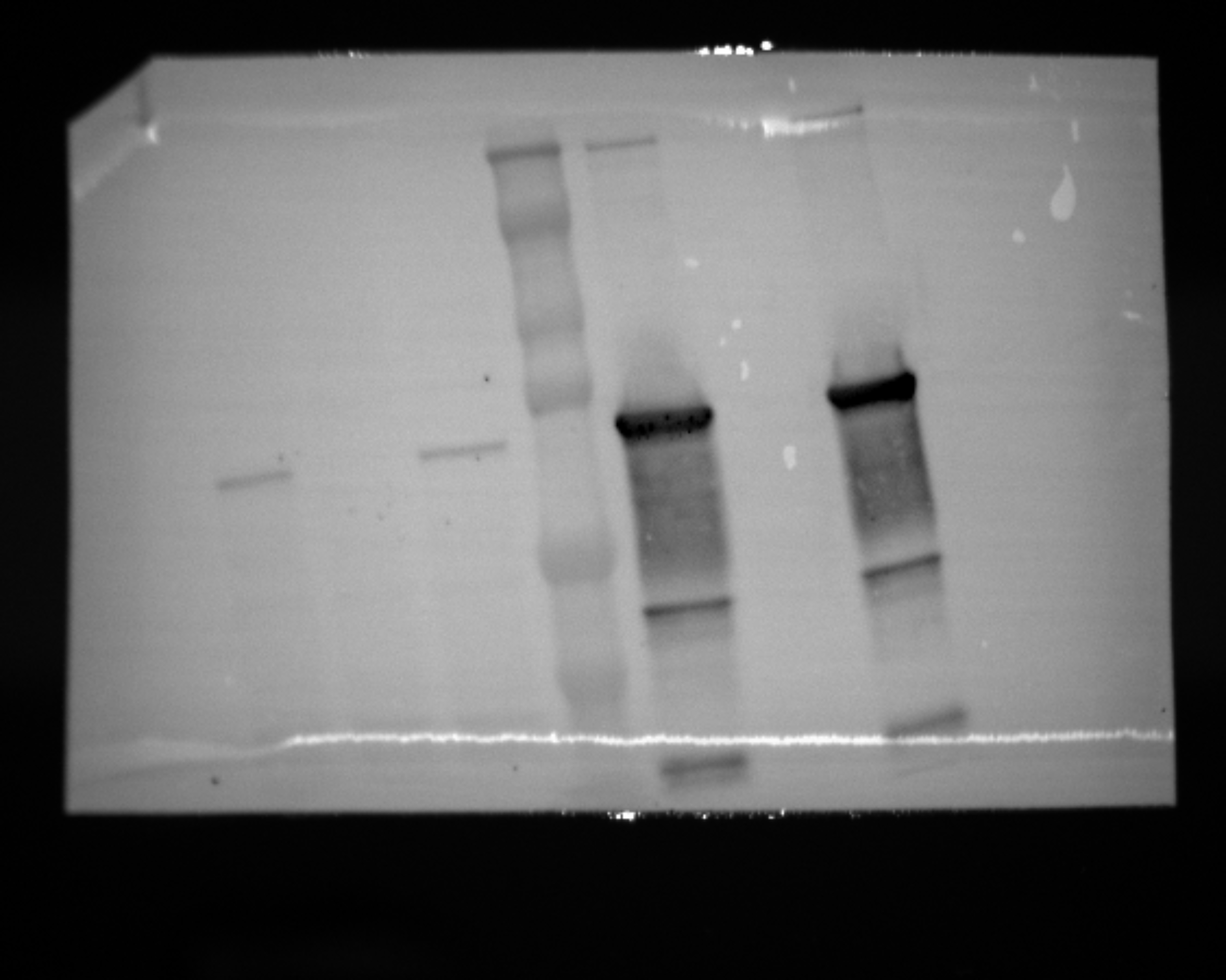

Supplement: Figure 4—figure supplement 1—source data 1. [file elife-101113-fig4-figsupp1-data1.zip › Figure 4-figure supplement 1-source data 1/Figure 4-figure supplement 1 Panel E GFP.tif]

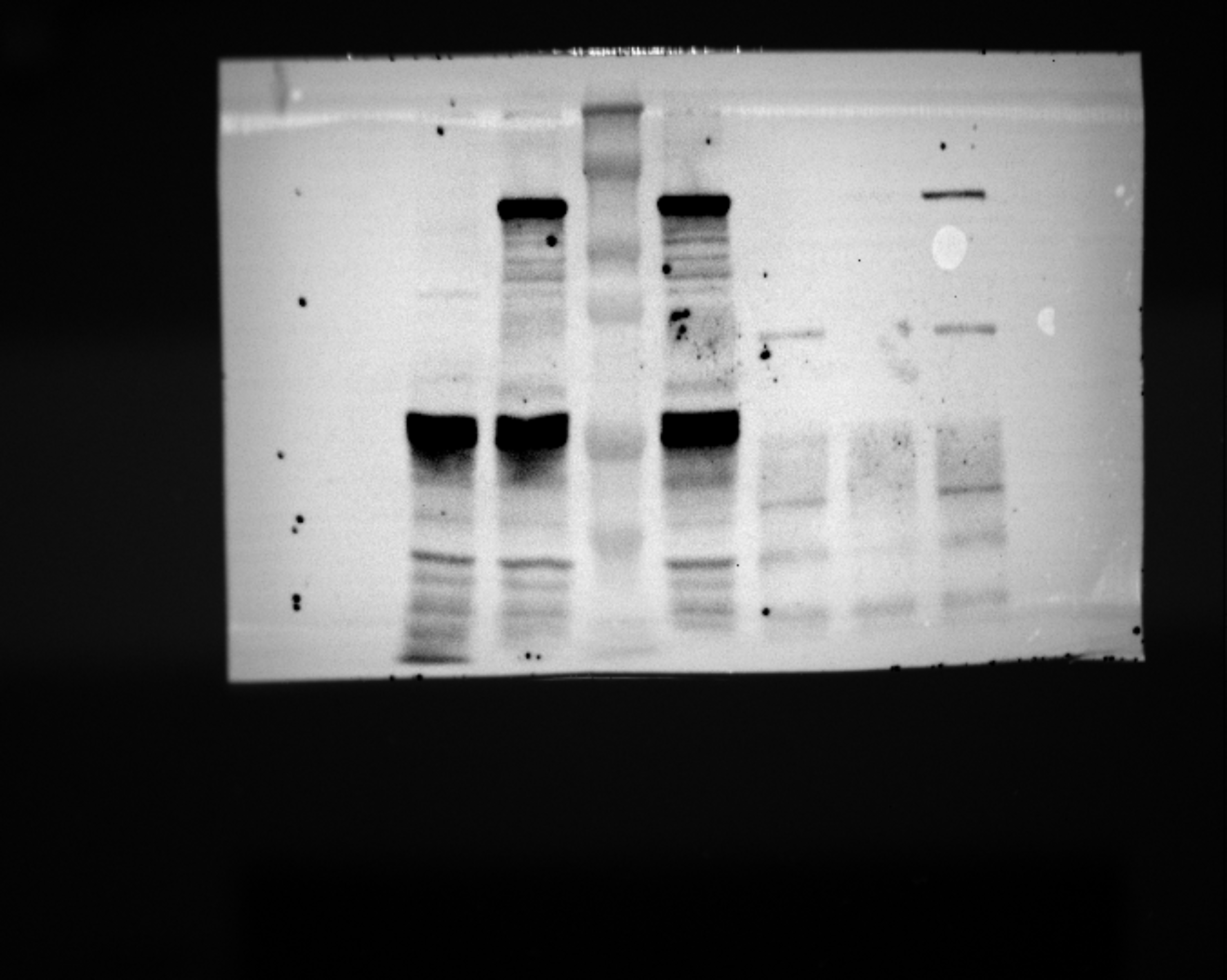

Supplement: Figure 4—figure supplement 1—source data 1. [file elife-101113-fig4-figsupp1-data1.zip › Figure 4-figure supplement 1-source data 1/Figure 4-figure supplement 1 Panel E Myc Tubulin.tif]

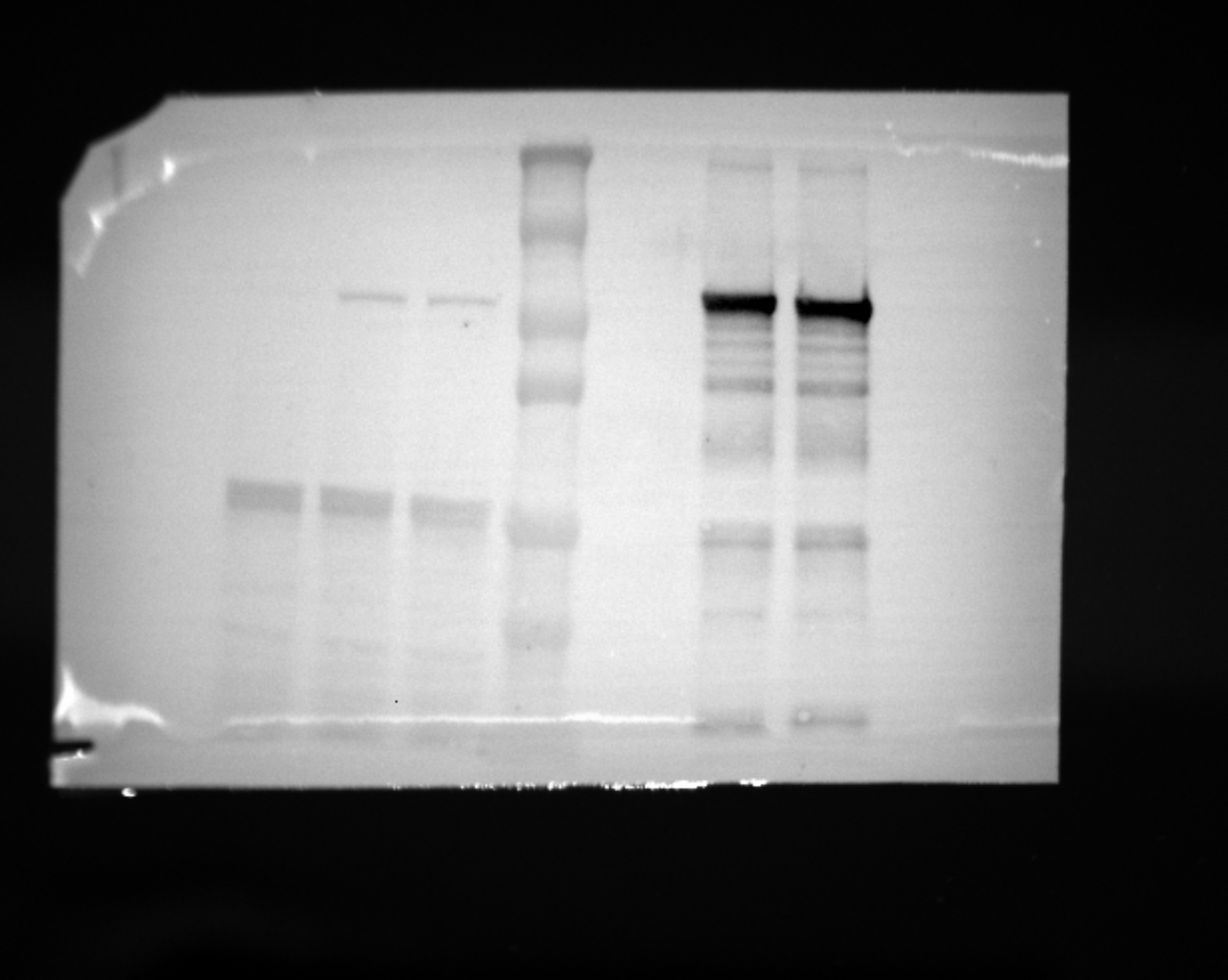

Supplement: Figure 4—figure supplement 1—source data 1. [file elife-101113-fig4-figsupp1-data1.zip › Figure 4-figure supplement 1-source data 1/Figure 4-figure supplement 1 Panel F GFP.tif]

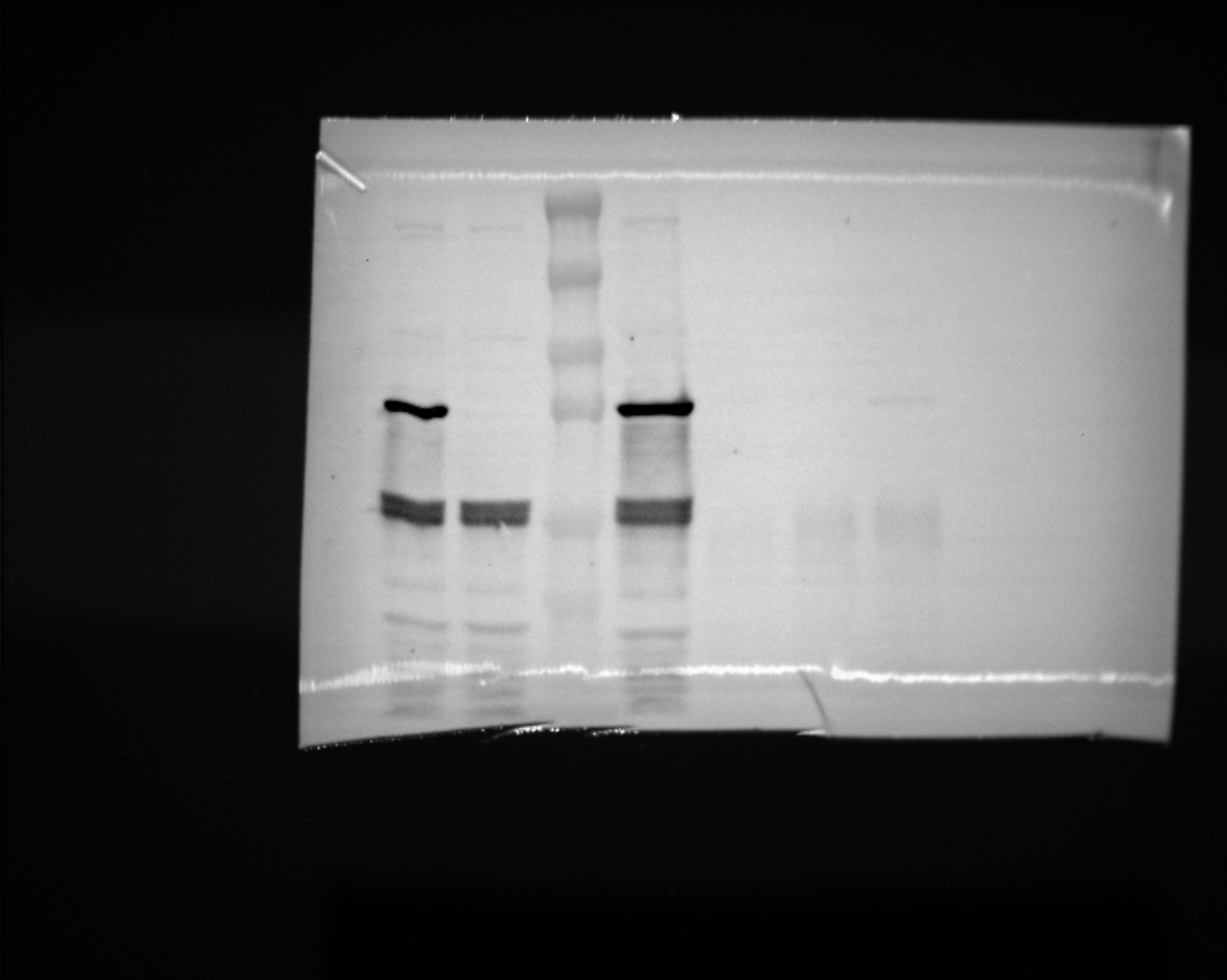

Supplement: Figure 4—figure supplement 1—source data 1. [file elife-101113-fig4-figsupp1-data1.zip › Figure 4-figure supplement 1-source data 1/Figure 4-figure supplement 1 Panel F Myc Tubulin.tif]

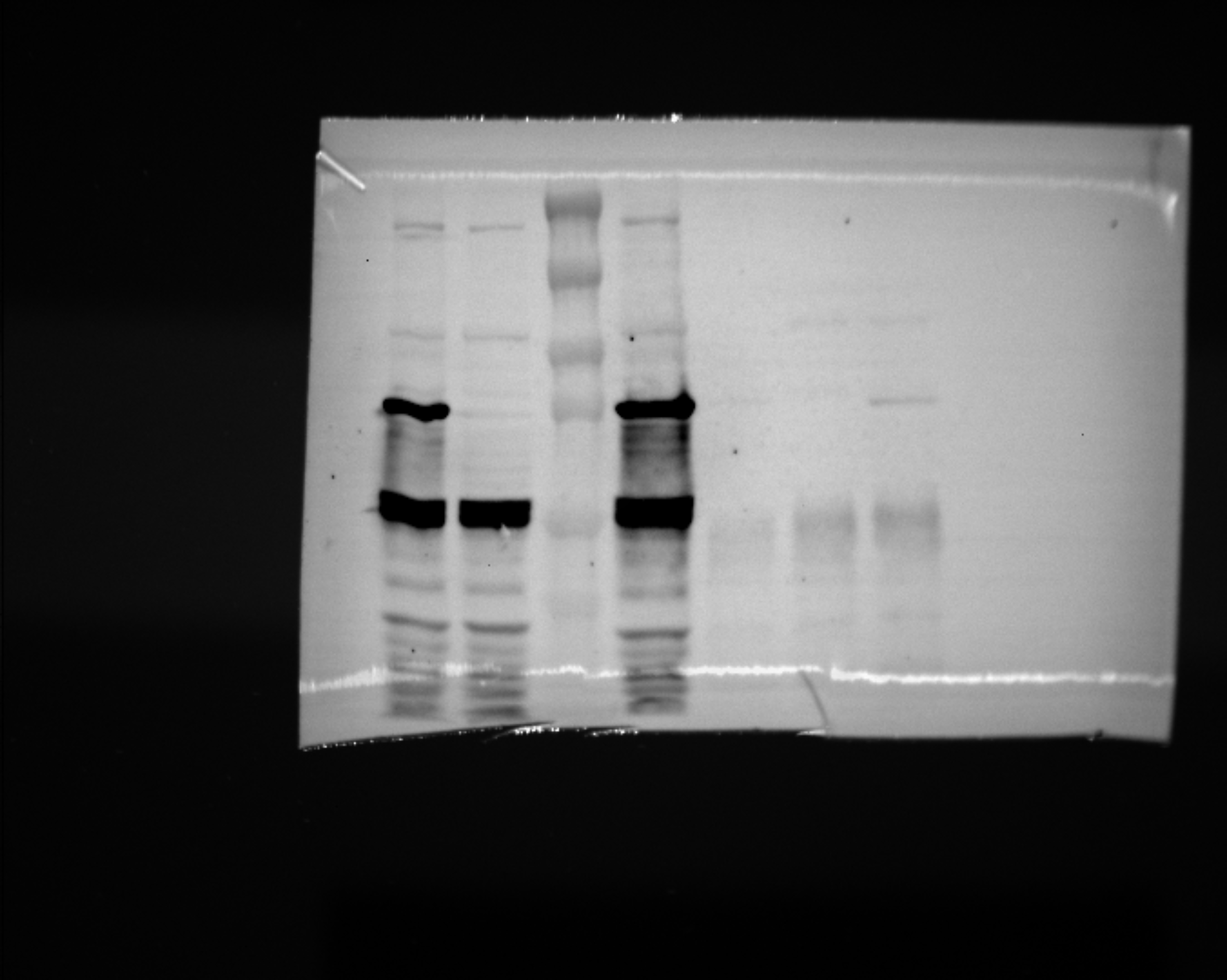

Supplement: Figure 4—figure supplement 1—source data 1. [file elife-101113-fig4-figsupp1-data1.zip › Figure 4-figure supplement 1-source data 1/Figure 4-figure supplement 1 Panel F Myc.tif]

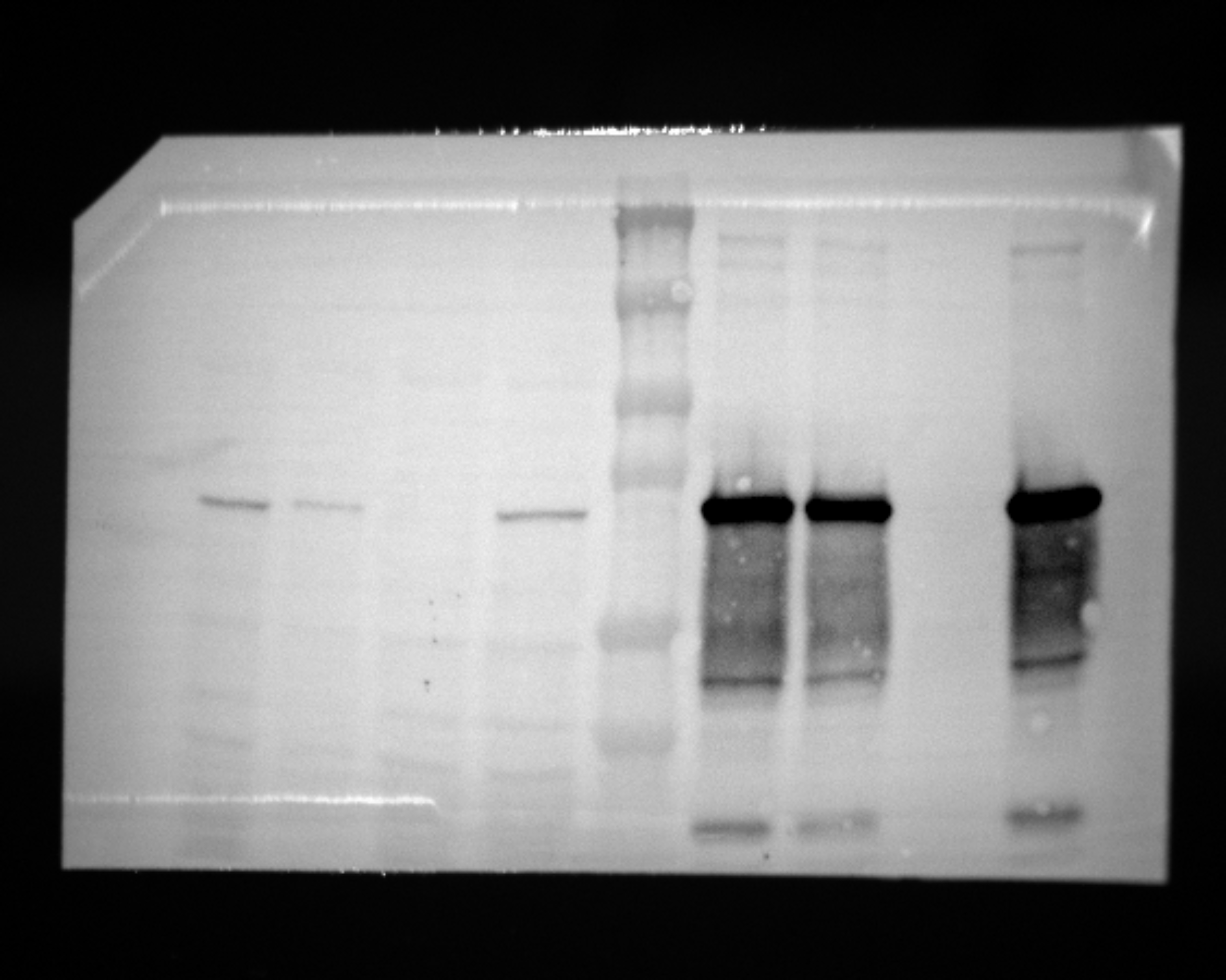

Supplement: Figure 4—figure supplement 1—source data 1. [file elife-101113-fig4-figsupp1-data1.zip › Figure 4-figure supplement 1-source data 1/Figure 4-figure supplement 1 Panel G GFP.tif]

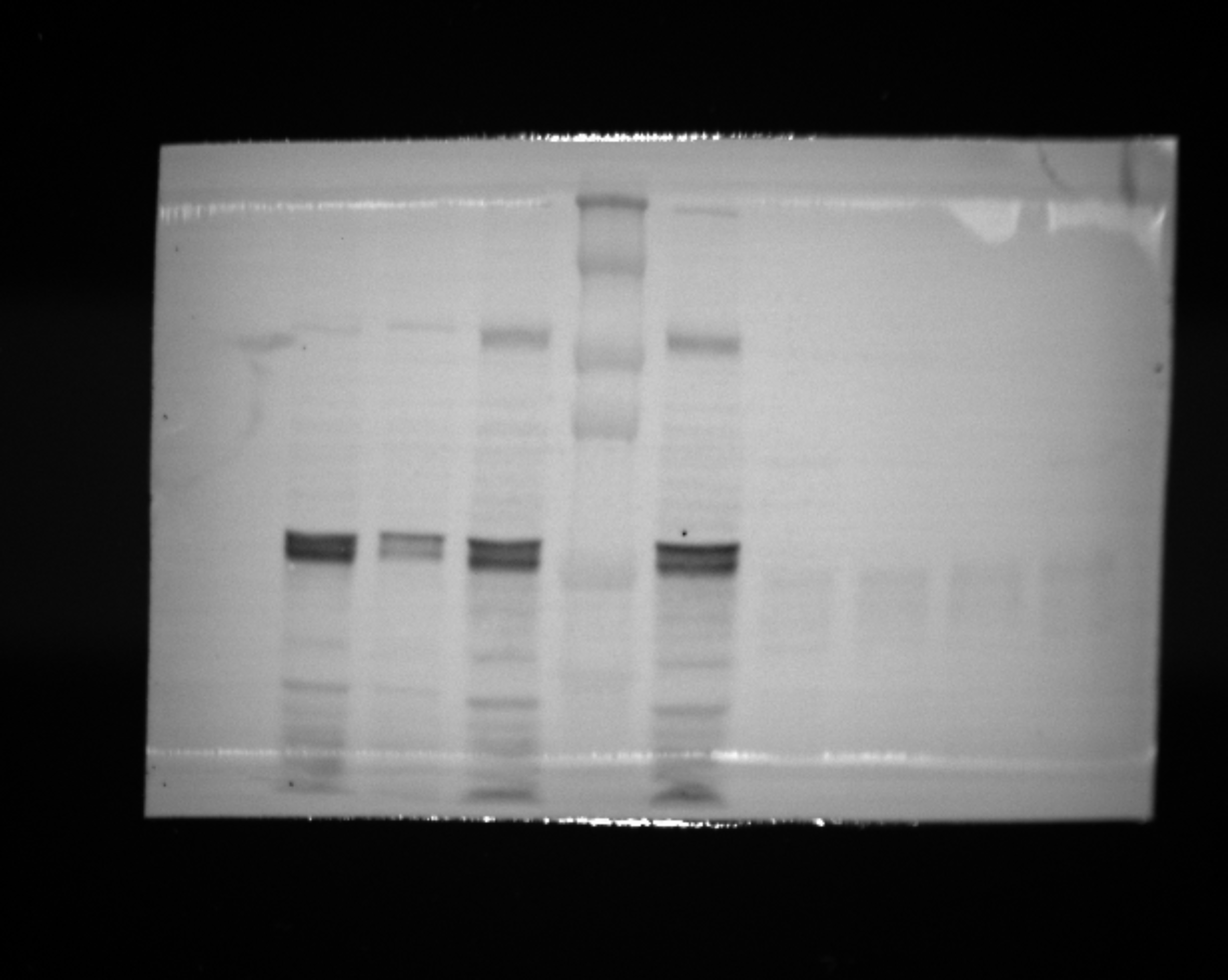

Supplement: Figure 4—figure supplement 1—source data 1. [file elife-101113-fig4-figsupp1-data1.zip › Figure 4-figure supplement 1-source data 1/Figure 4-figure supplement 1 Panel G Myc Tubulin.tif]

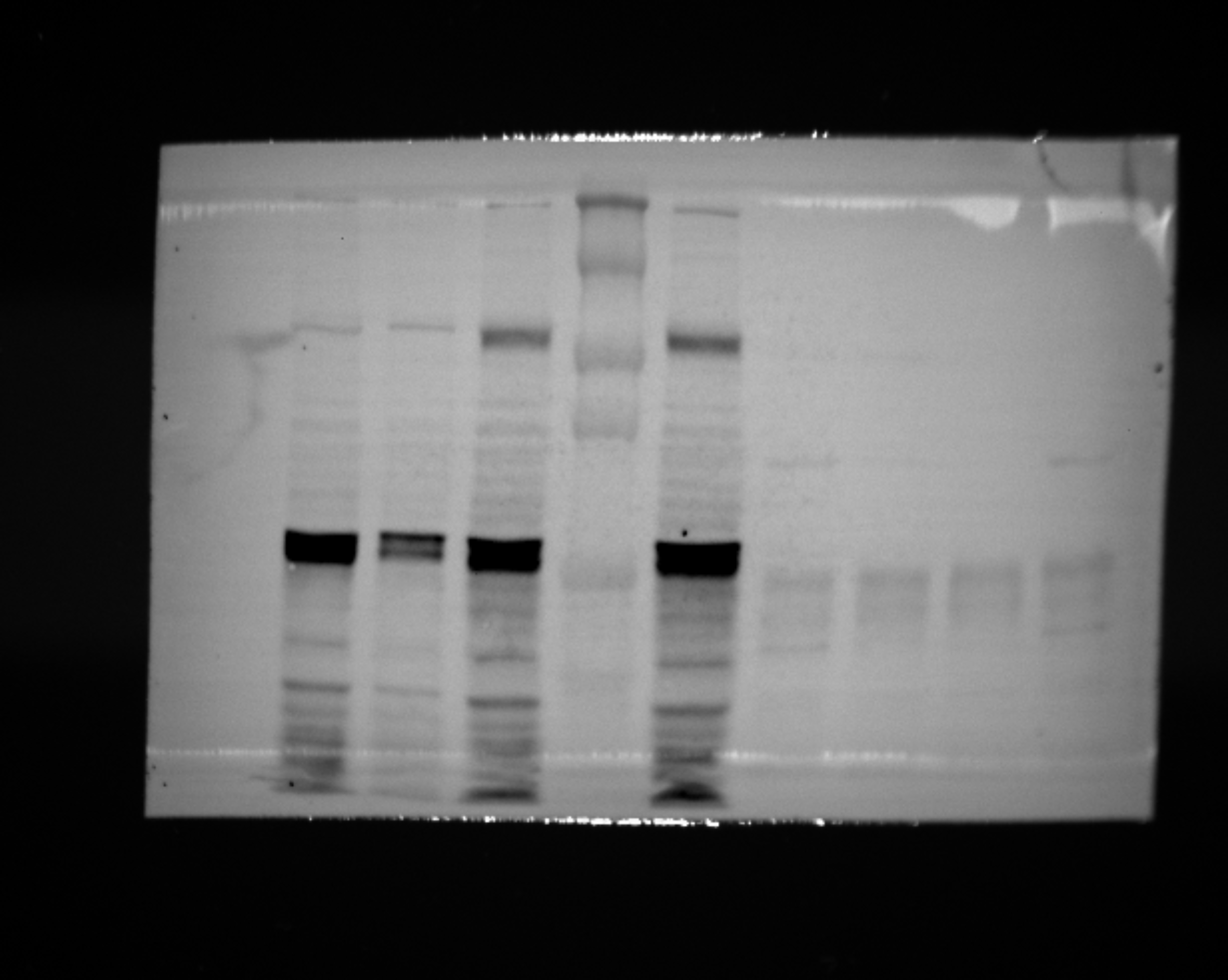

Supplement: Figure 4—figure supplement 1—source data 1. [file elife-101113-fig4-figsupp1-data1.zip › Figure 4-figure supplement 1-source data 1/Figure 4-figure supplement 1 Panel G Myc.tif]

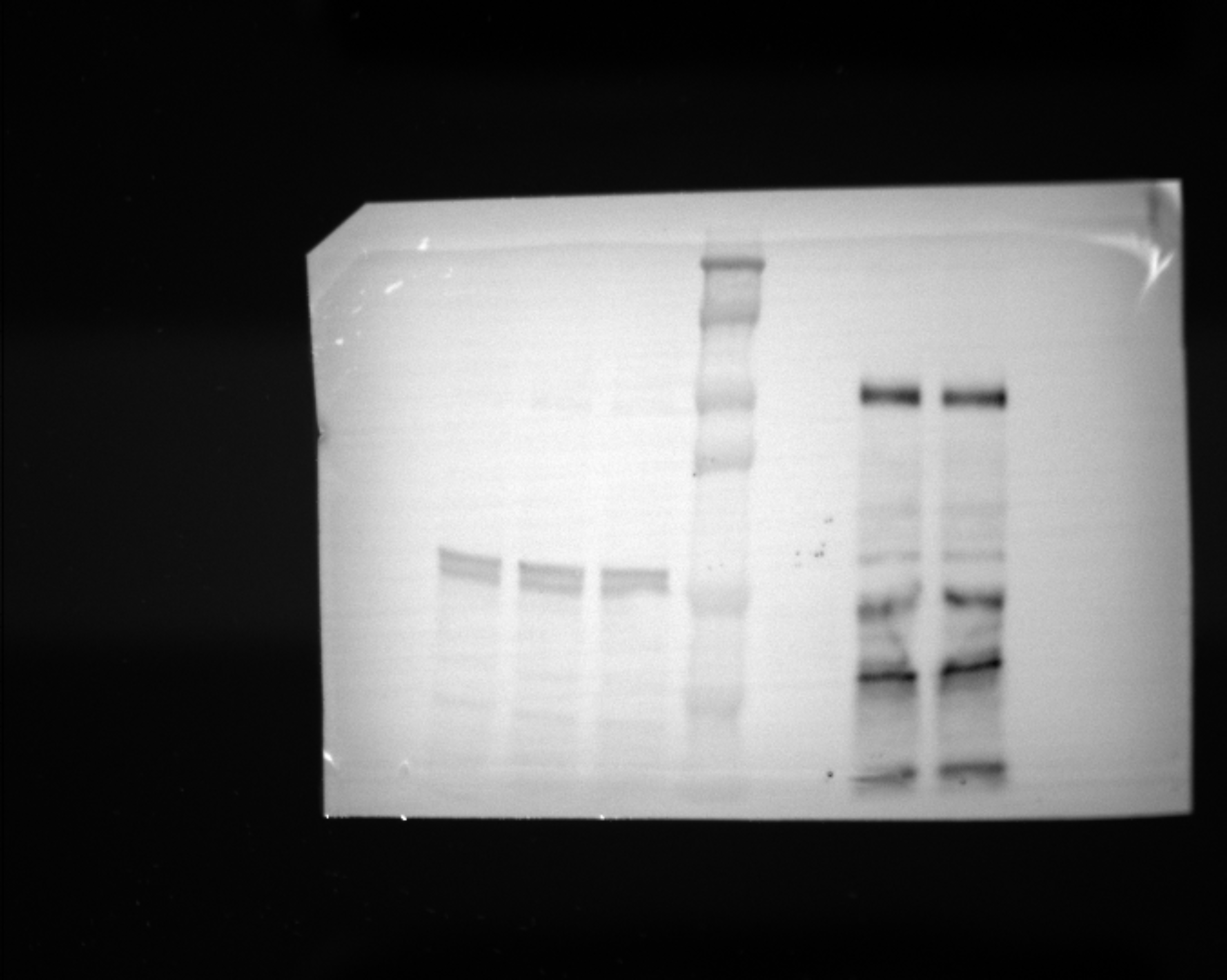

Supplement: Figure 4—figure supplement 1—source data 1. [file elife-101113-fig4-figsupp1-data1.zip › Figure 4-figure supplement 1-source data 1/Figure 4-figure supplement 1 Panel H GFP Tubulin.tif]

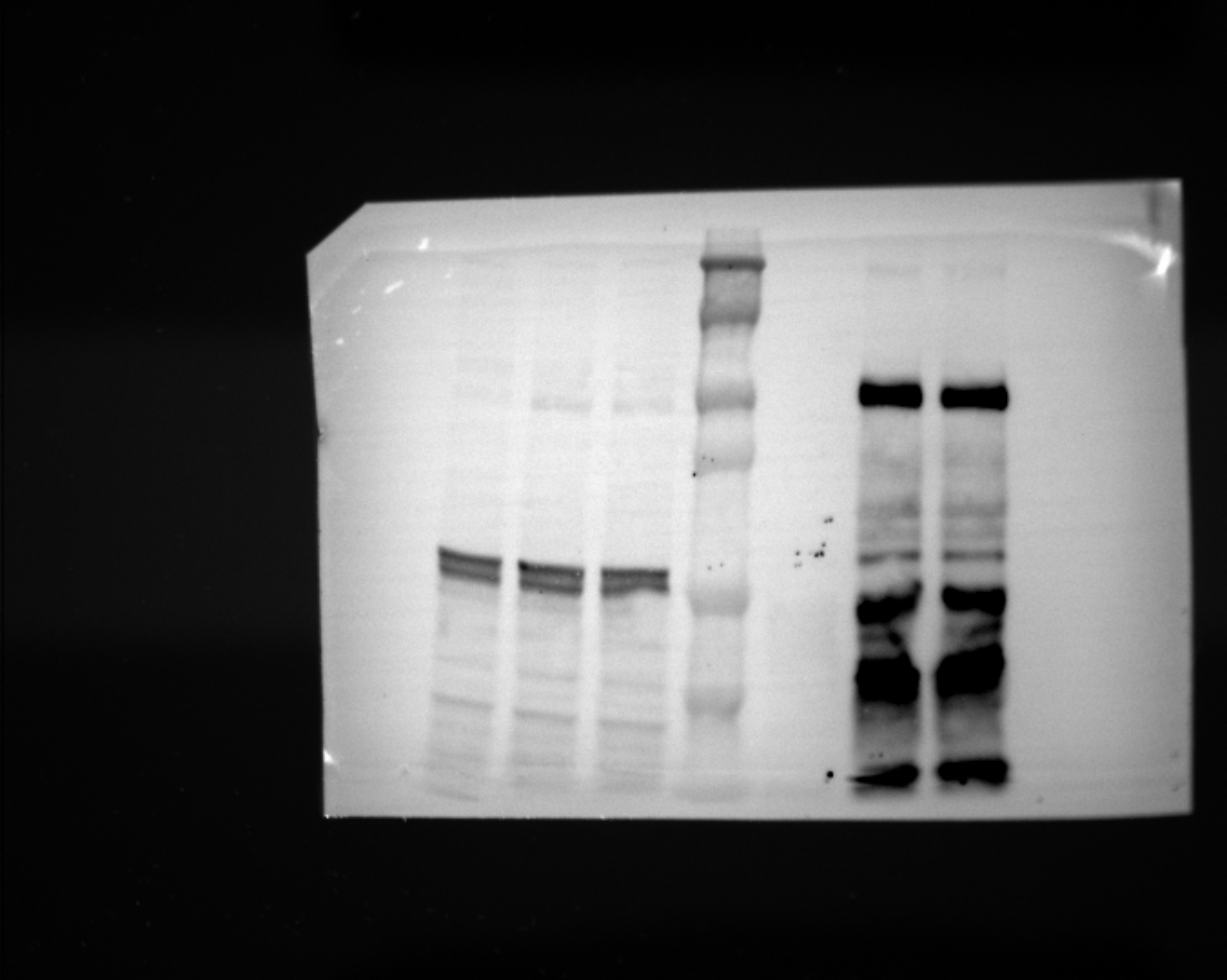

Supplement: Figure 4—figure supplement 1—source data 1. [file elife-101113-fig4-figsupp1-data1.zip › Figure 4-figure supplement 1-source data 1/Figure 4-figure supplement 1 Panel H GFP.tif]

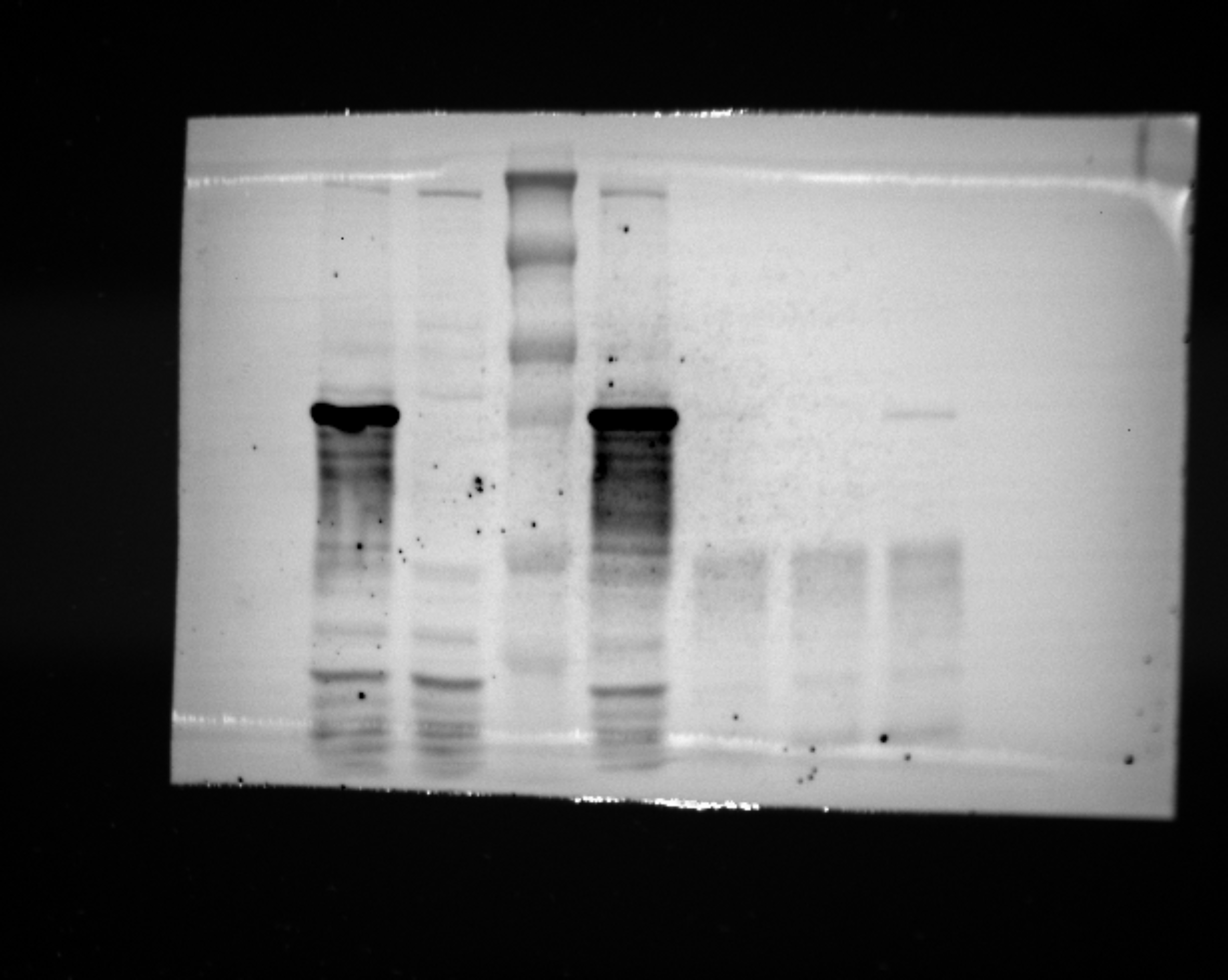

Supplement: Figure 4—figure supplement 1—source data 1. [file elife-101113-fig4-figsupp1-data1.zip › Figure 4-figure supplement 1-source data 1/Figure 4-figure supplement 1 Panel H Myc.tif]
