# Supplementary material for: Septins function in exocytosis via physical interactions with the exocyst complex in fission yeast cytokinesis
Source: eLife. 2025 Oct 31;13:RP101113. doi: 10.7554/eLife.101113 (PMC12578440; doi:10.7554/eLife.101113)
Supplement: Figure 4—source data 2. [file elife-101113-fig4-data2.zip › Figure 4-source data 2/Figure 4 source files labeled.pdf]

Figure 4 panel A labeled

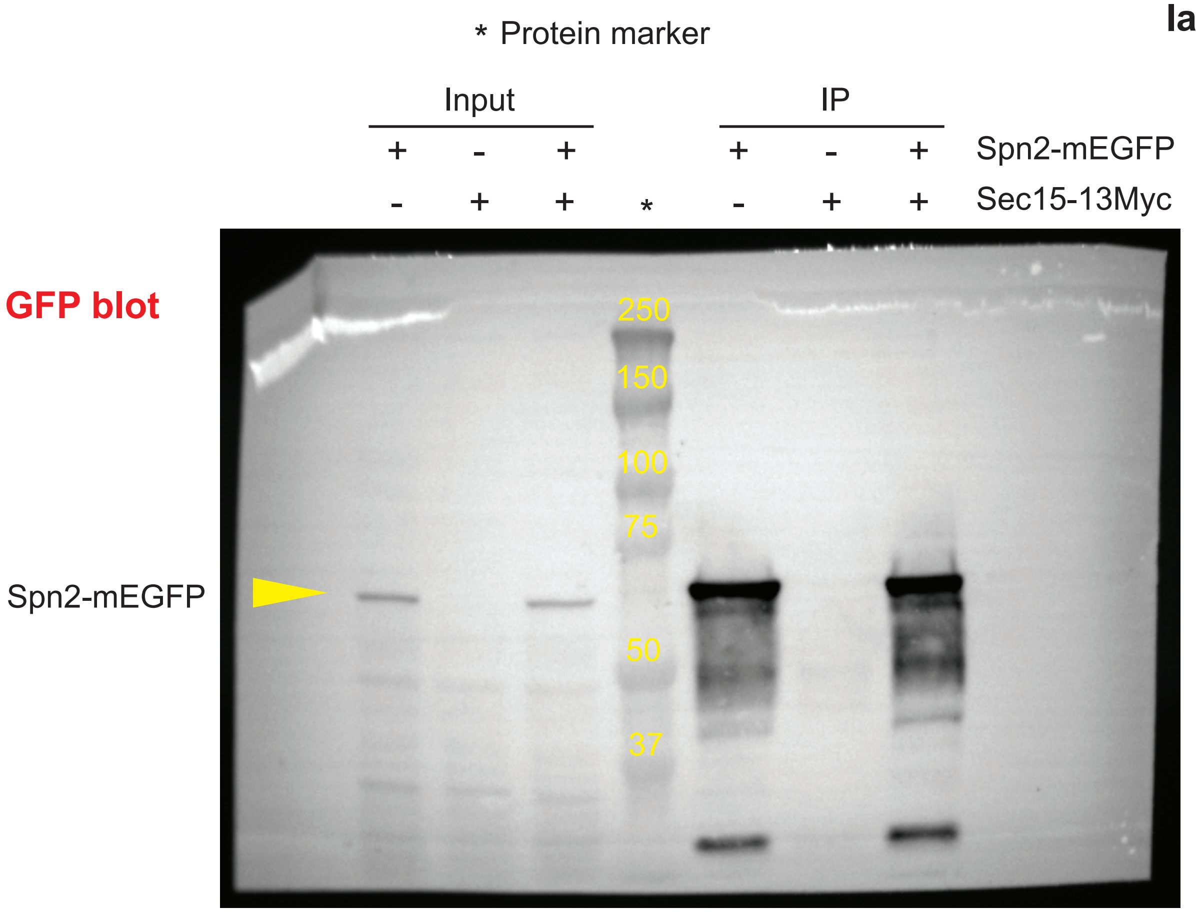

Note: White illumination near the edges of the blots are unwanted reflection of liquid from membrane and does not appear in chemiluminisence channel.

Figure 4 panel A labeled

* Protein marker

	Input				IP			
	+	-		+	+	-	+	Spn2-mEGFP
	-	+	*	+	-	+	+	Sec15-13Myc

Myc blot

Sec15-13Myc

Tubulin
(Not used in paper)

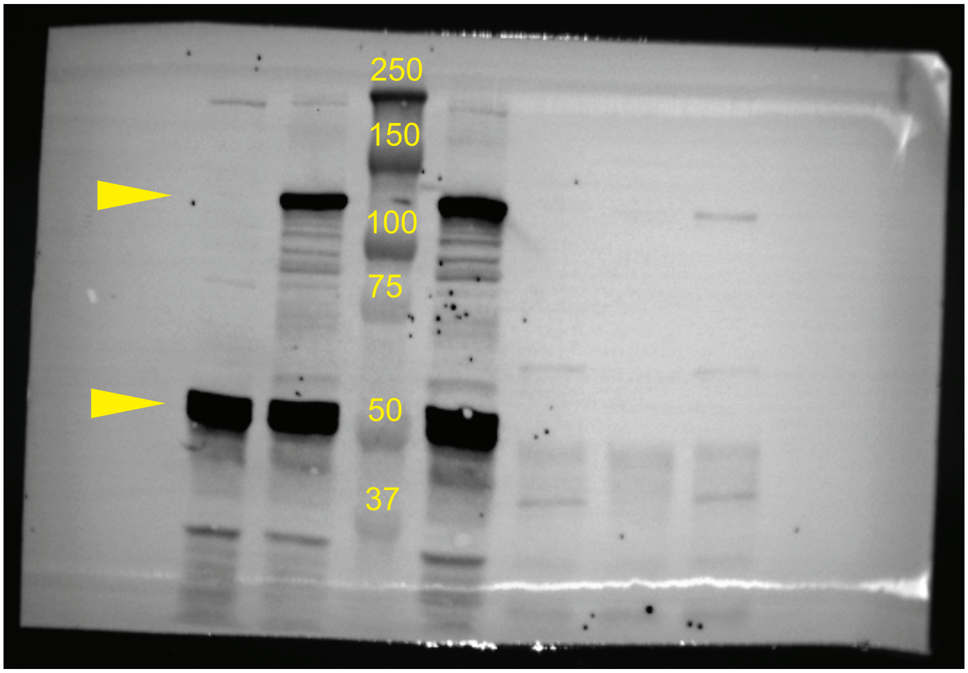

Long exposure

	Input				IP			
	+	-		+	+	-	+	Spn2-mEGFP
	-	+	*	+	-	+	+	Sec15-13Myc

Myc blot

Sec15-13Myc

Tubulin

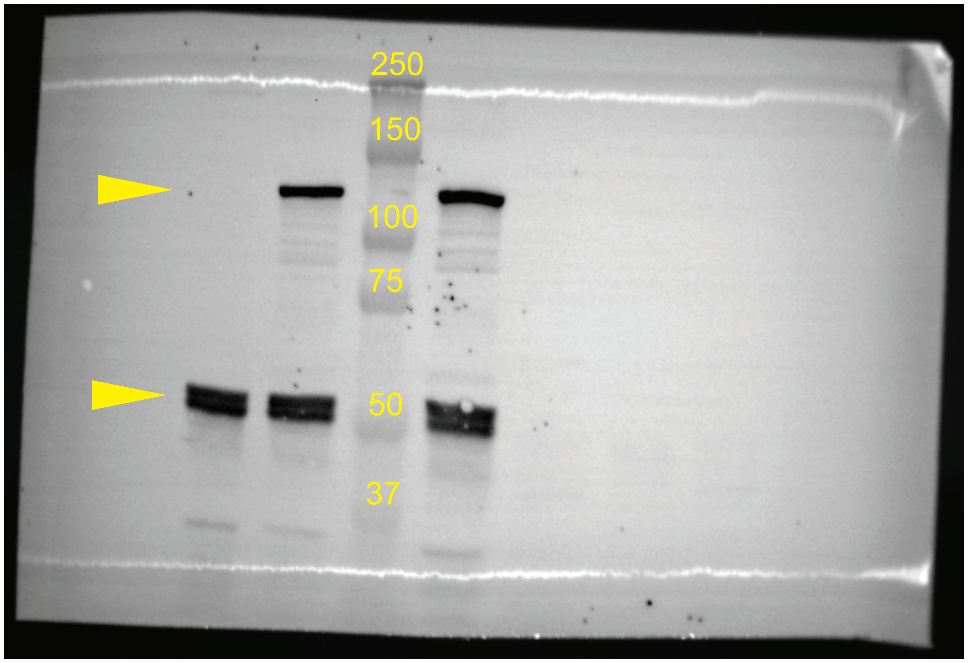

Short exposure

Note: White illumination near the edges of the blots are unwanted reflection of liquid from membrane and does not appear in chemiluminisence channel.

Figure 4 panel B labeled

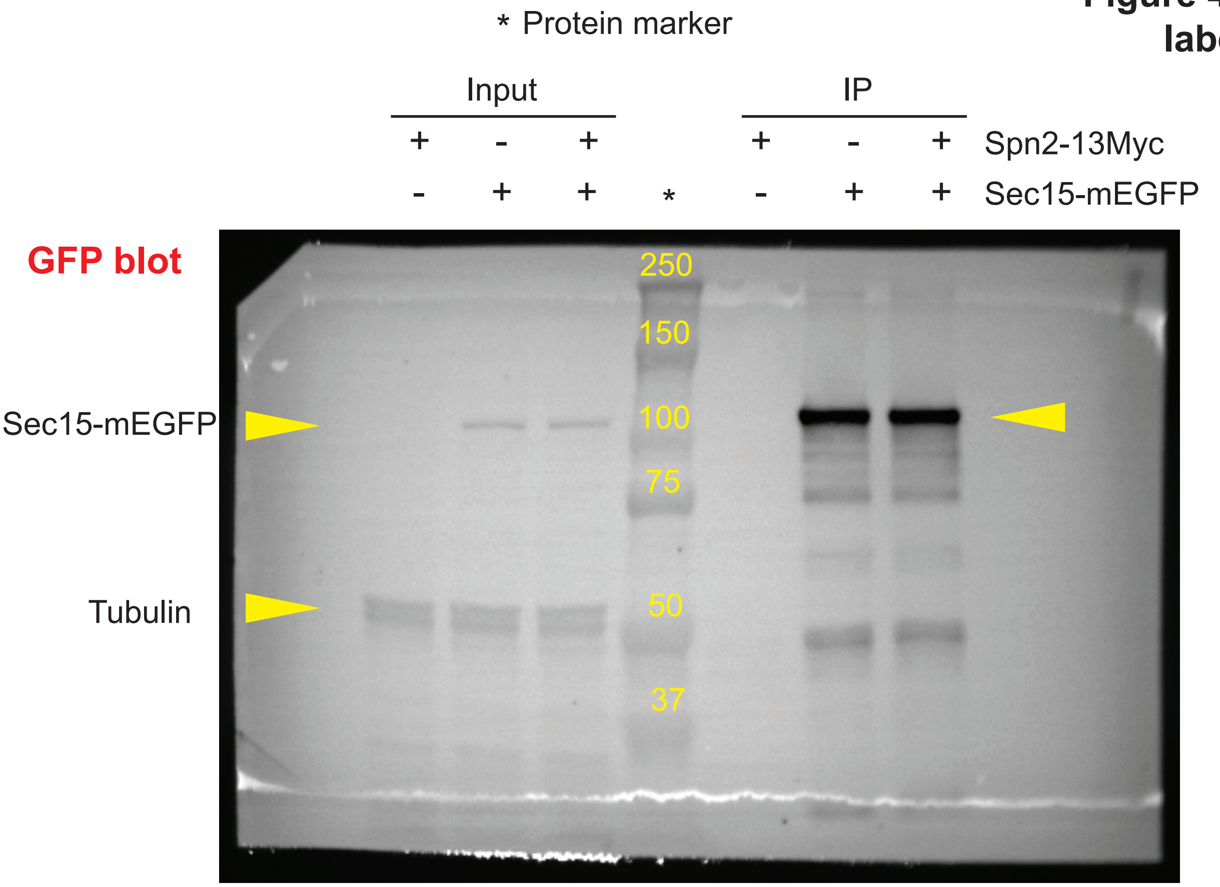

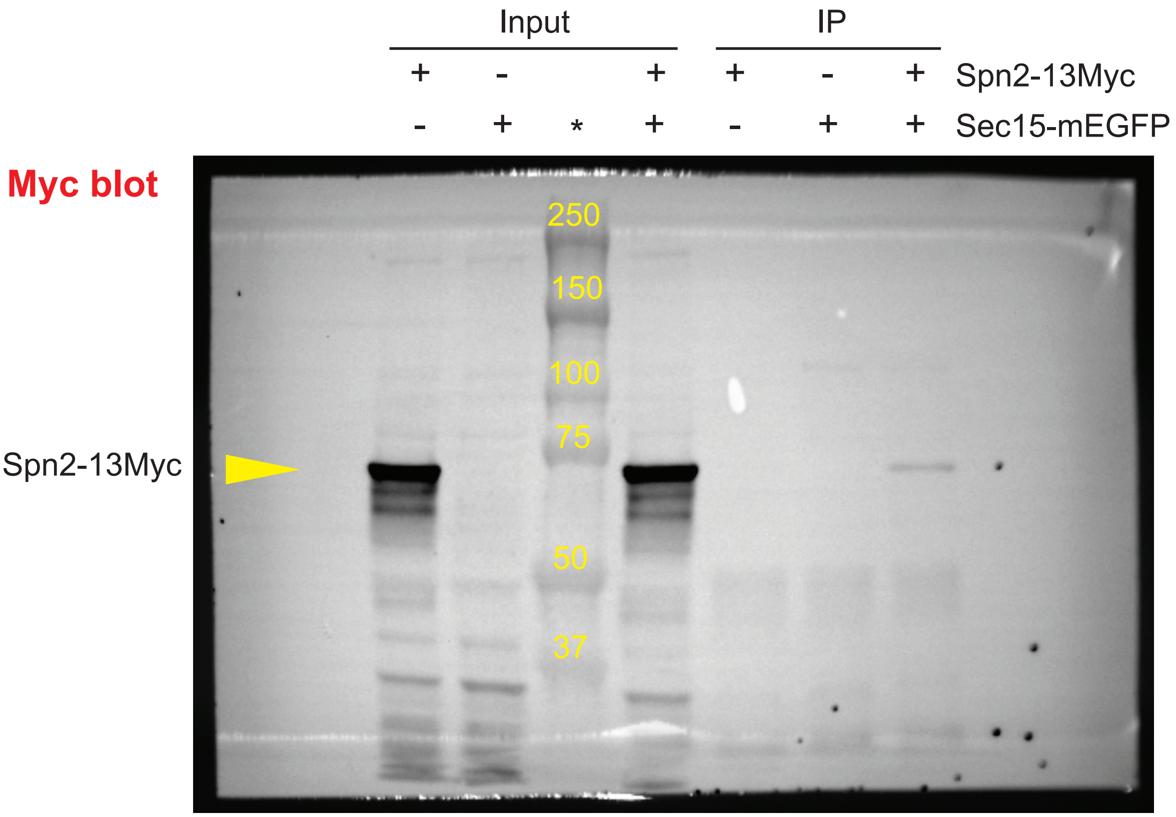

Note: White illumination near the edges of the blots are unwanted reflection of liquid from membrane and does not appear in chemiluminisence channel.

Figure 4 panel C labeled

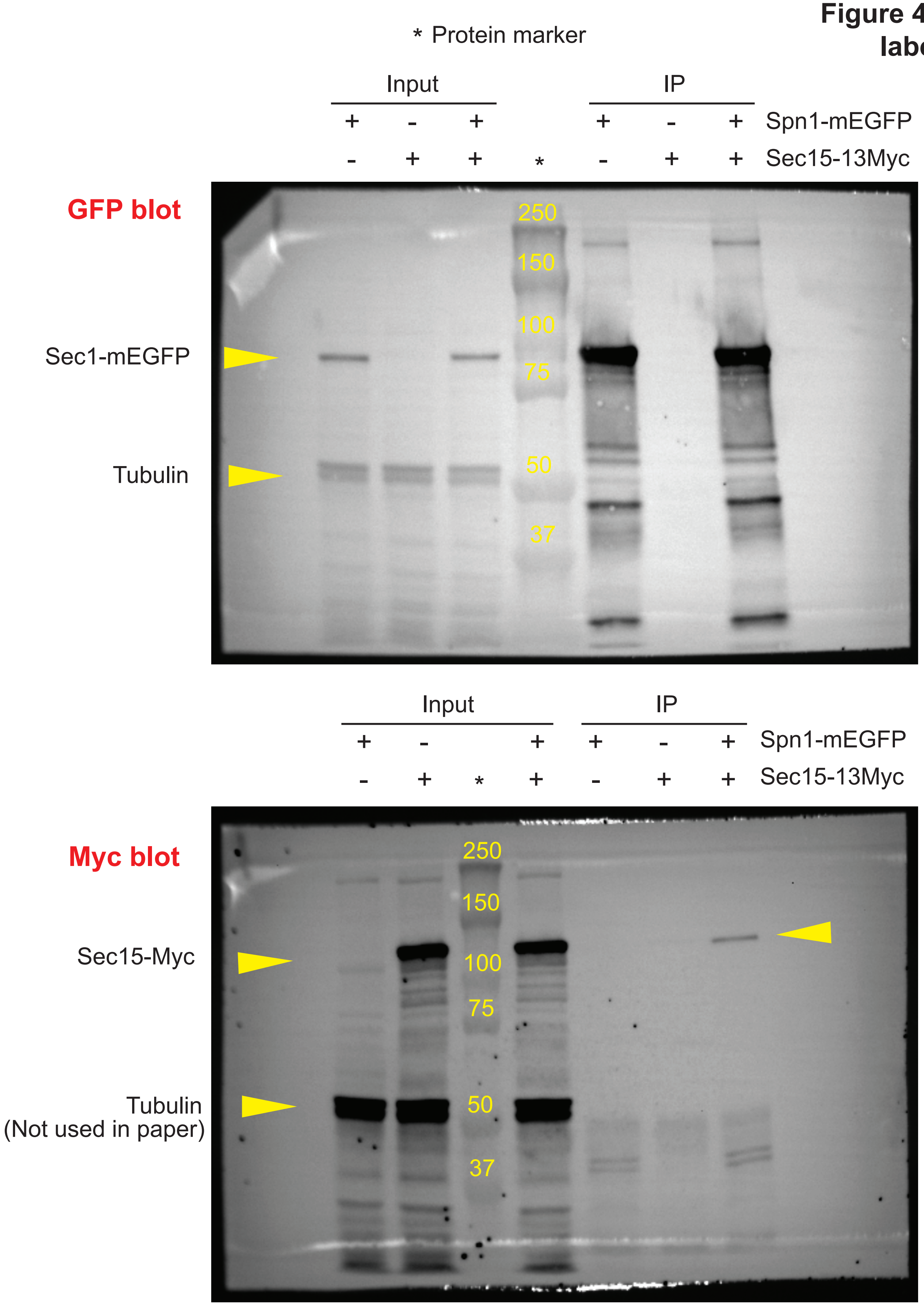

Note: White illumination near the edges of the blots are unwanted reflection of liquid from membrane and does not appear in chemiluminisence channel.

Figure 4 panel D labeled

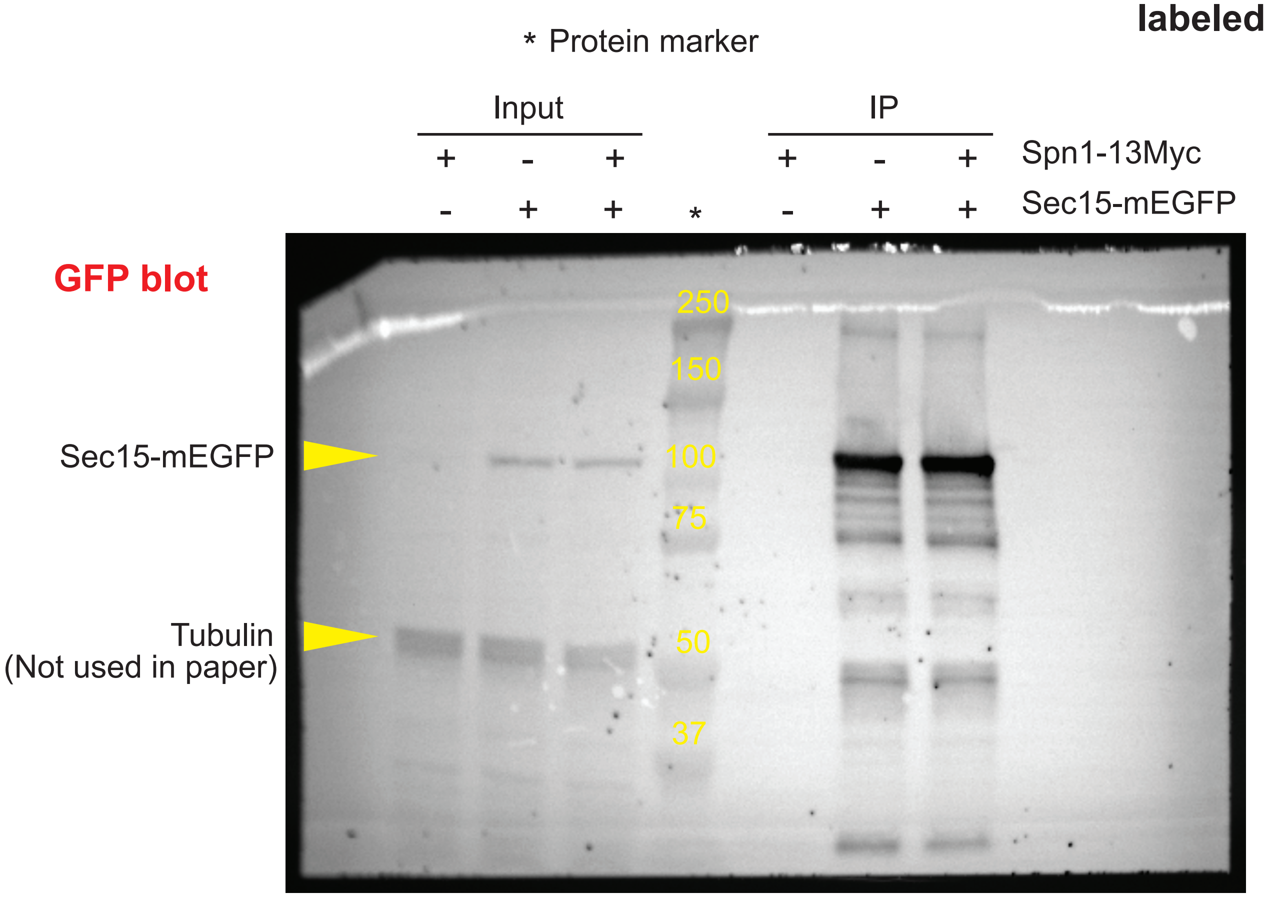

Note: White illumination near the edges of the blots are unwanted reflection of liquid from membrane and does not appear in chemiluminisence channel.

Figure 4 panel D labeled

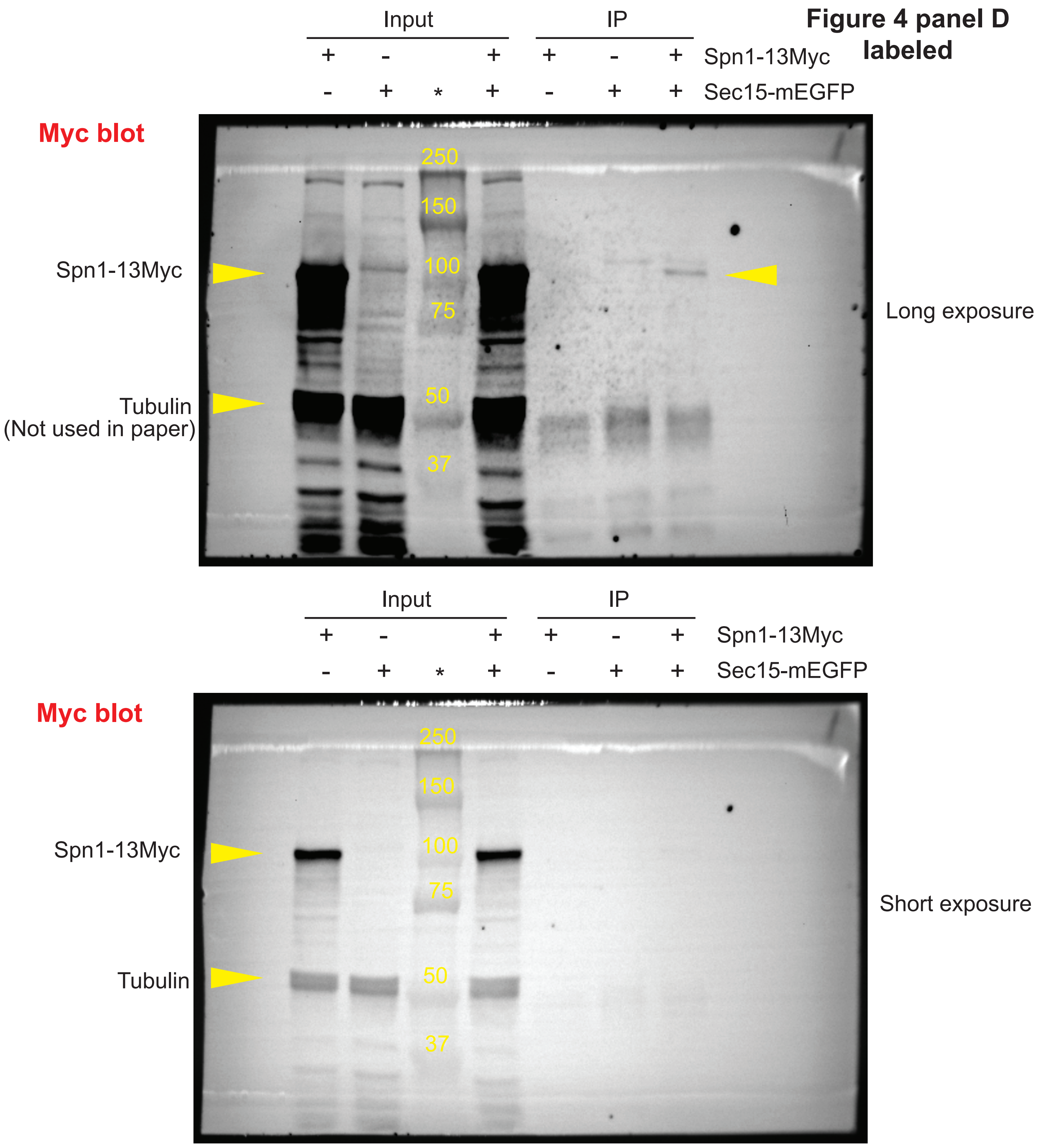

Note: White illumination near the edges of the blots are unwanted reflection of liquid from membrane and does not appear in chemiluminisence channel.
