# Supplementary material for: Septins function in exocytosis via physical interactions with the exocyst complex in fission yeast cytokinesis
Source: eLife. 2025 Oct 31;13:RP101113. doi: 10.7554/eLife.101113 (PMC12578440; doi:10.7554/eLife.101113)
Supplement: Figure 4—figure supplement 1—source data 2. [file elife-101113-fig4-figsupp1-data2.zip › Figure 4-figure supplement 1-source data 2/Figure 4-figure supplement 1-source files labeled.pdf]

Figure 4-figure supplement 1 panel A labeled

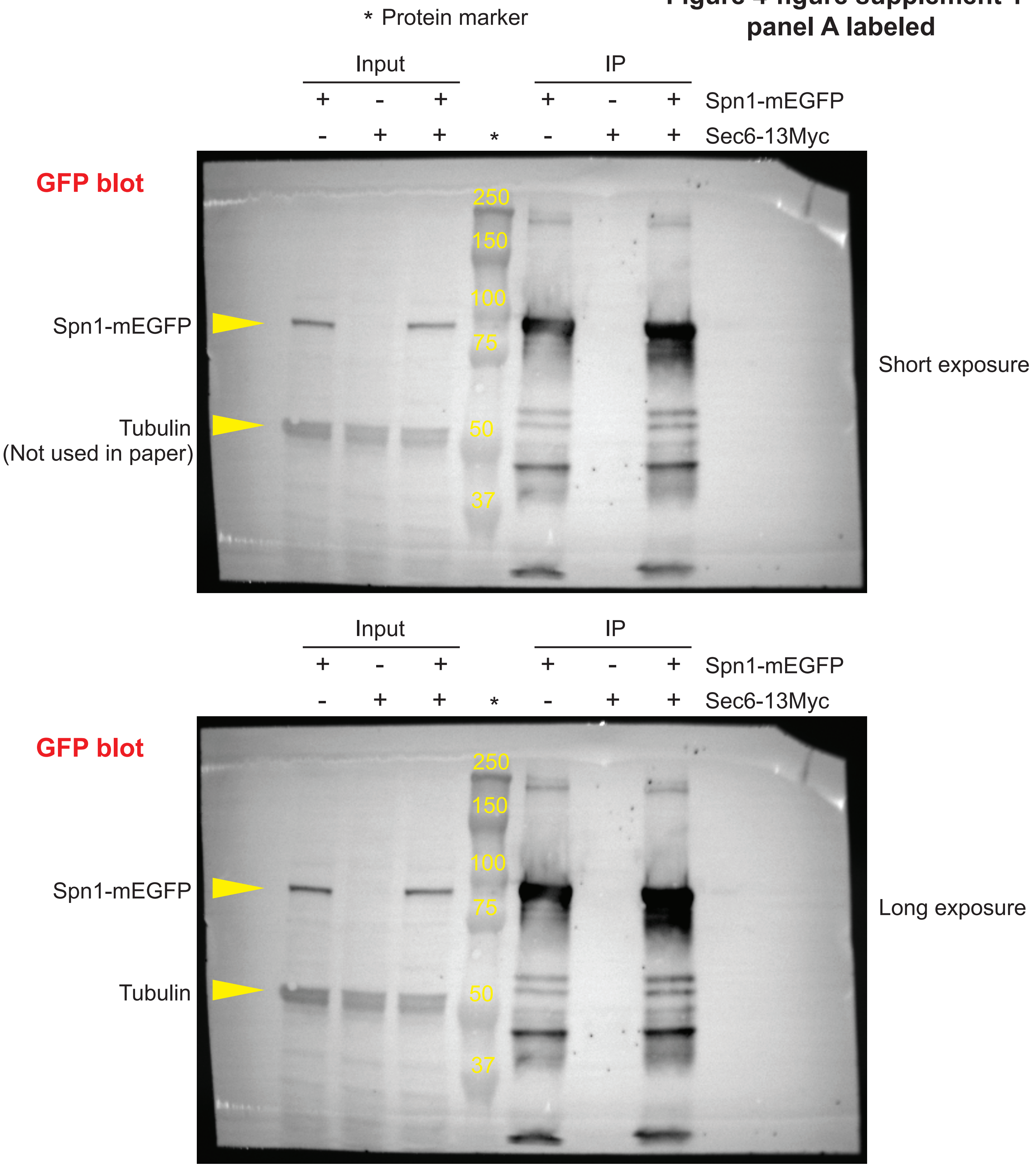

Note: White illumination near the edges of the blots are unwanted reflection of liquid from membrane and does not appear in chemiluminisence channel.

Figure 4-figure supplement 1 panel A labeled

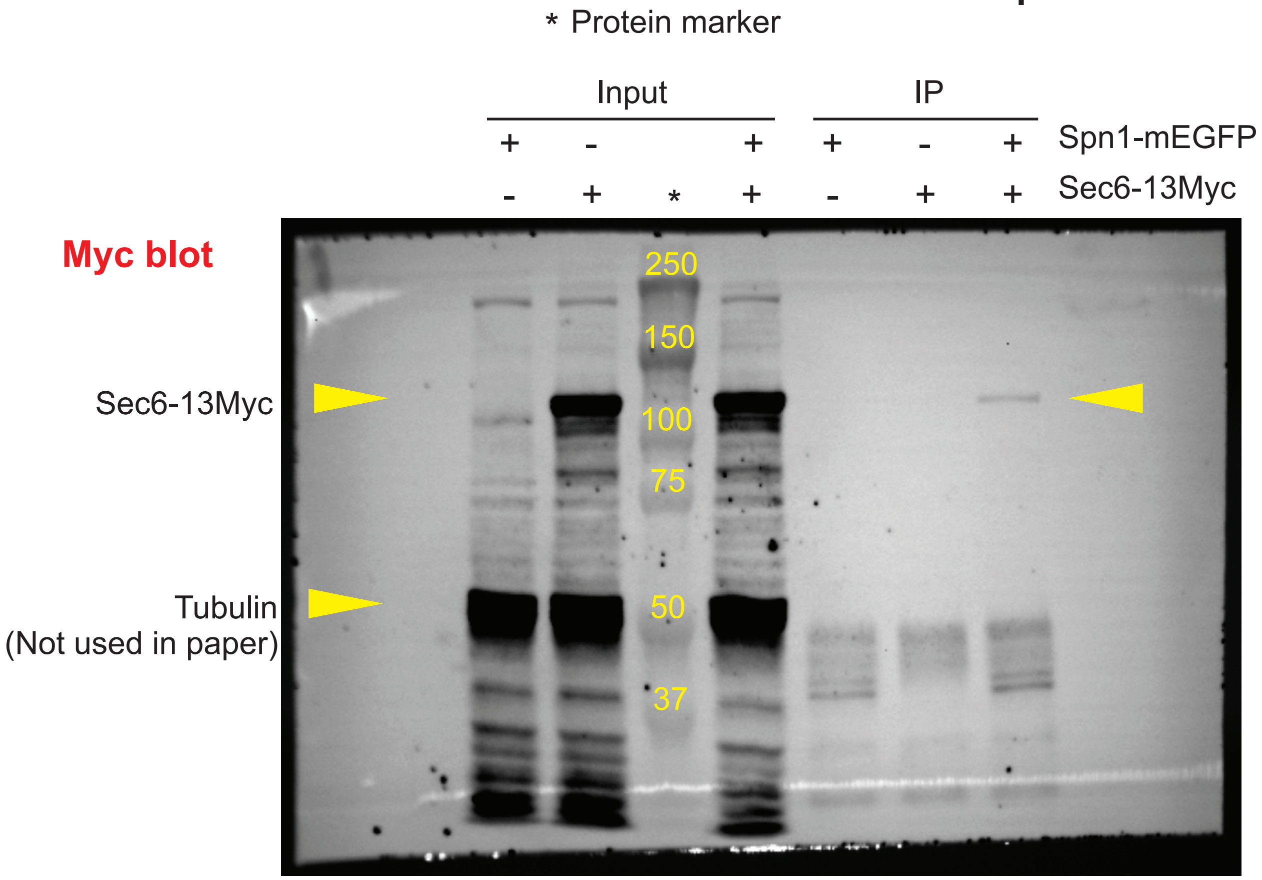

Note: White illumination near the edges of the blots are unwanted reflection of liquid from membrane and does not appear in chemiluminisence channel.

Figure 4-figure supplement 1 panel B labeled

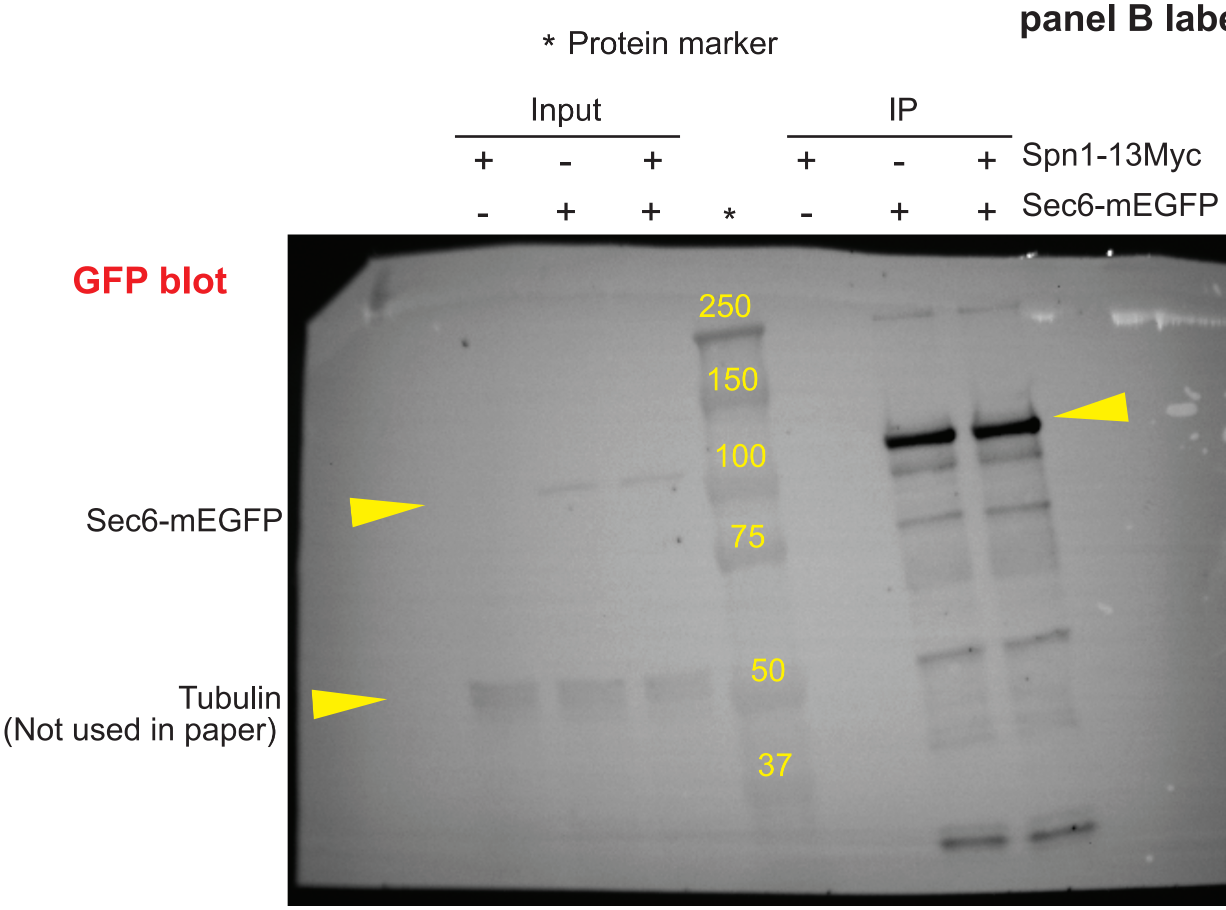

Note: White illumination near the edges of the blots are unwanted reflection of liquid from membrane and does not appear in chemiluminisence channel.

Figure 4-figure supplement 1 panel B labeled

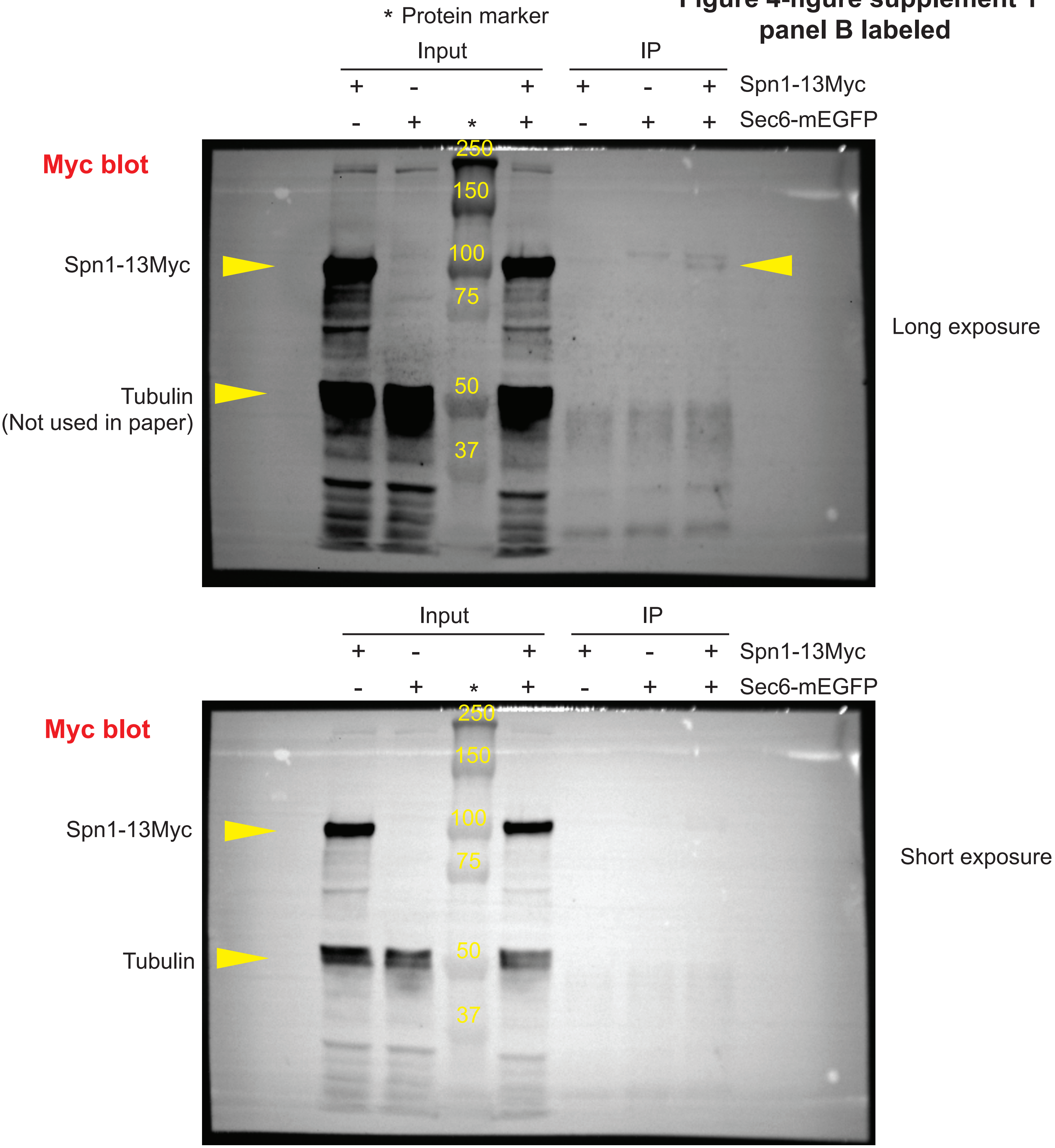

Note: White illumination near the edges of the blots are unwanted reflection of liquid from membrane and does not appear in chemiluminisence channel.

Figure 4-figure supplement 1 panel C labeled

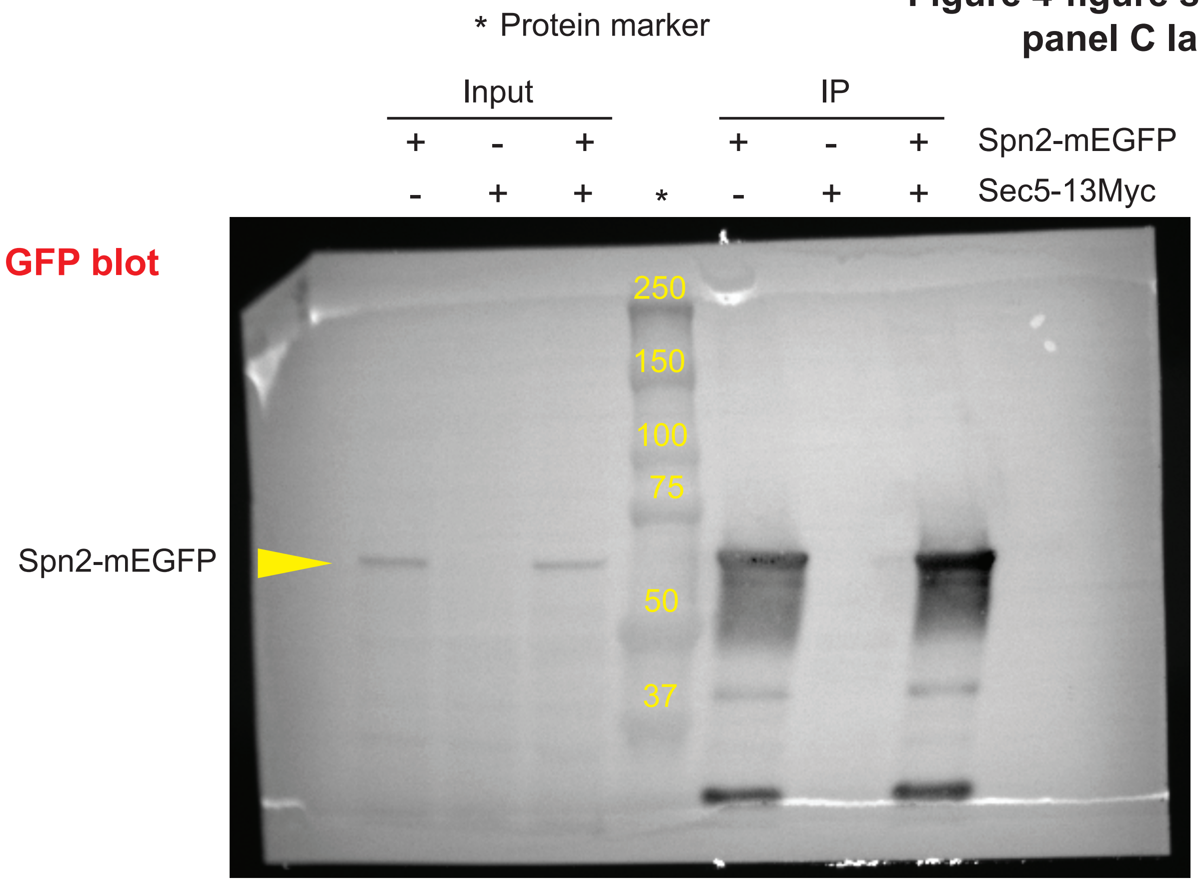

Note: White illumination near the edges of the blots are unwanted reflection of liquid from membrane and does not appear in chemiluminisence channel.

Figure 4-figure supplement 1 panel C labeled

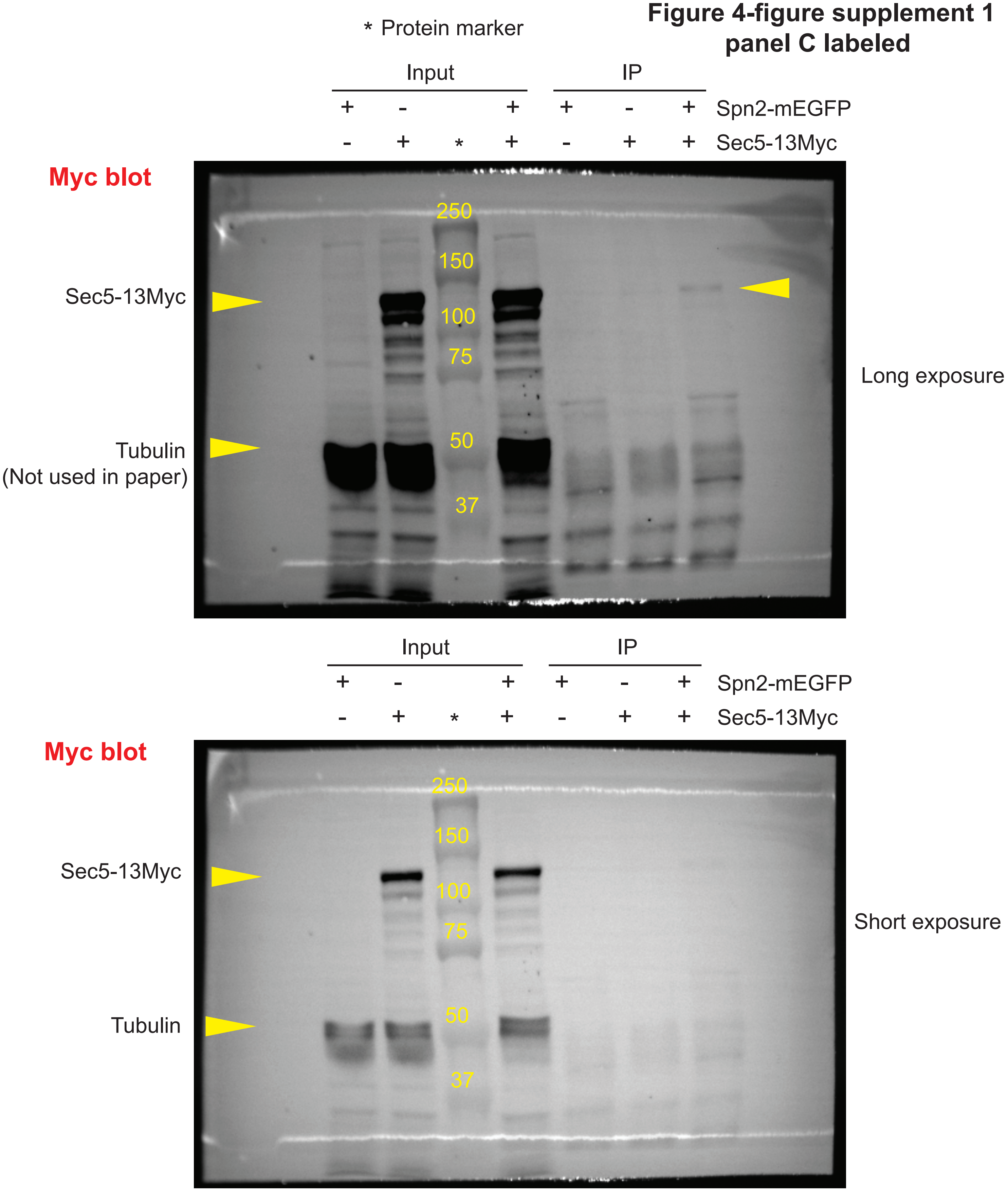

Note: White illumination near the edges of the blots are unwanted reflection of liquid from membrane and does not appear in chemiluminisence channel.

Figure 4-figure supplement 1 panel D labeled

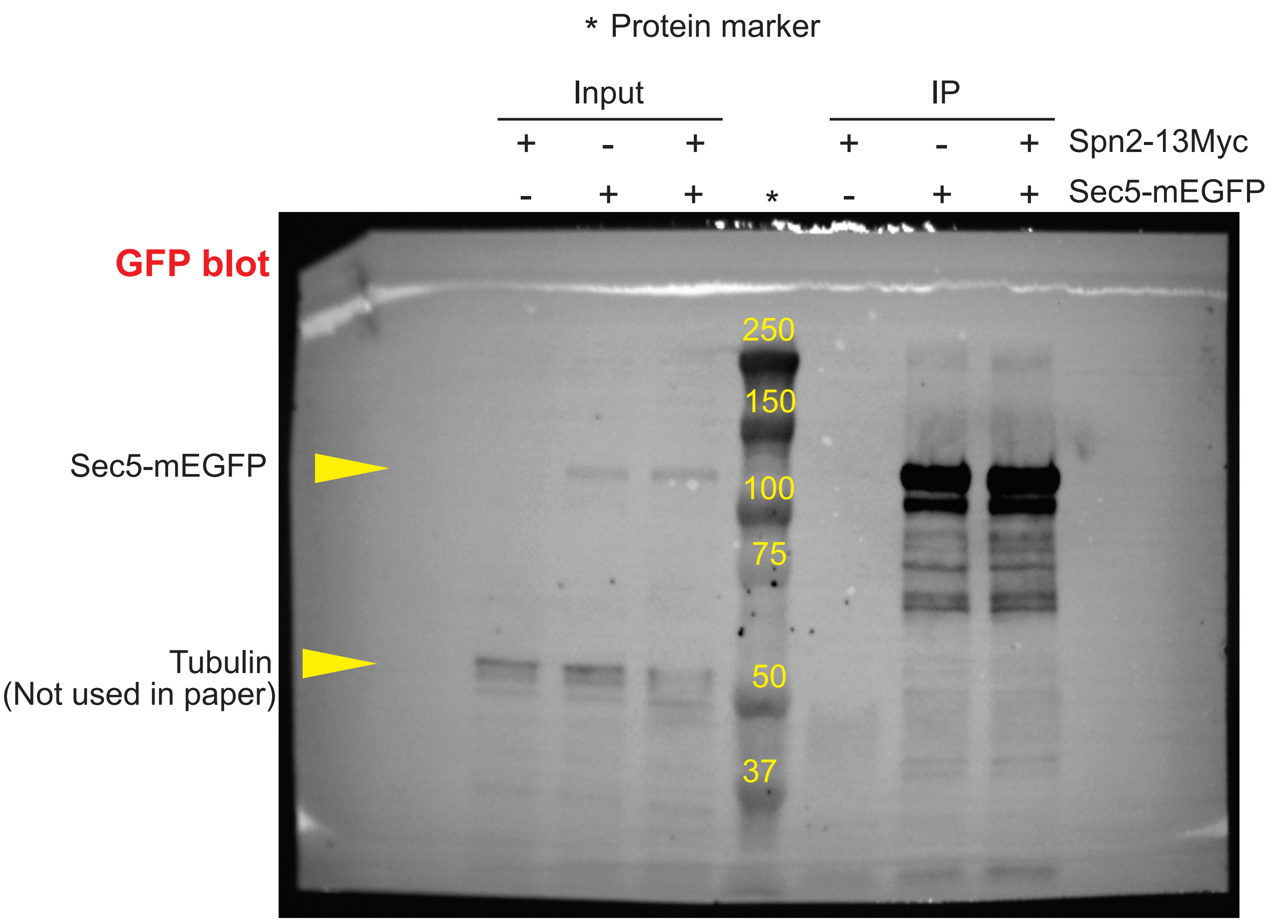

Note: White illumination near the edges of the blots are unwanted reflection of liquid from membrane and does not appear in chemiluminisence channel.

Figure 4-figure supplement 1 panel D labeled

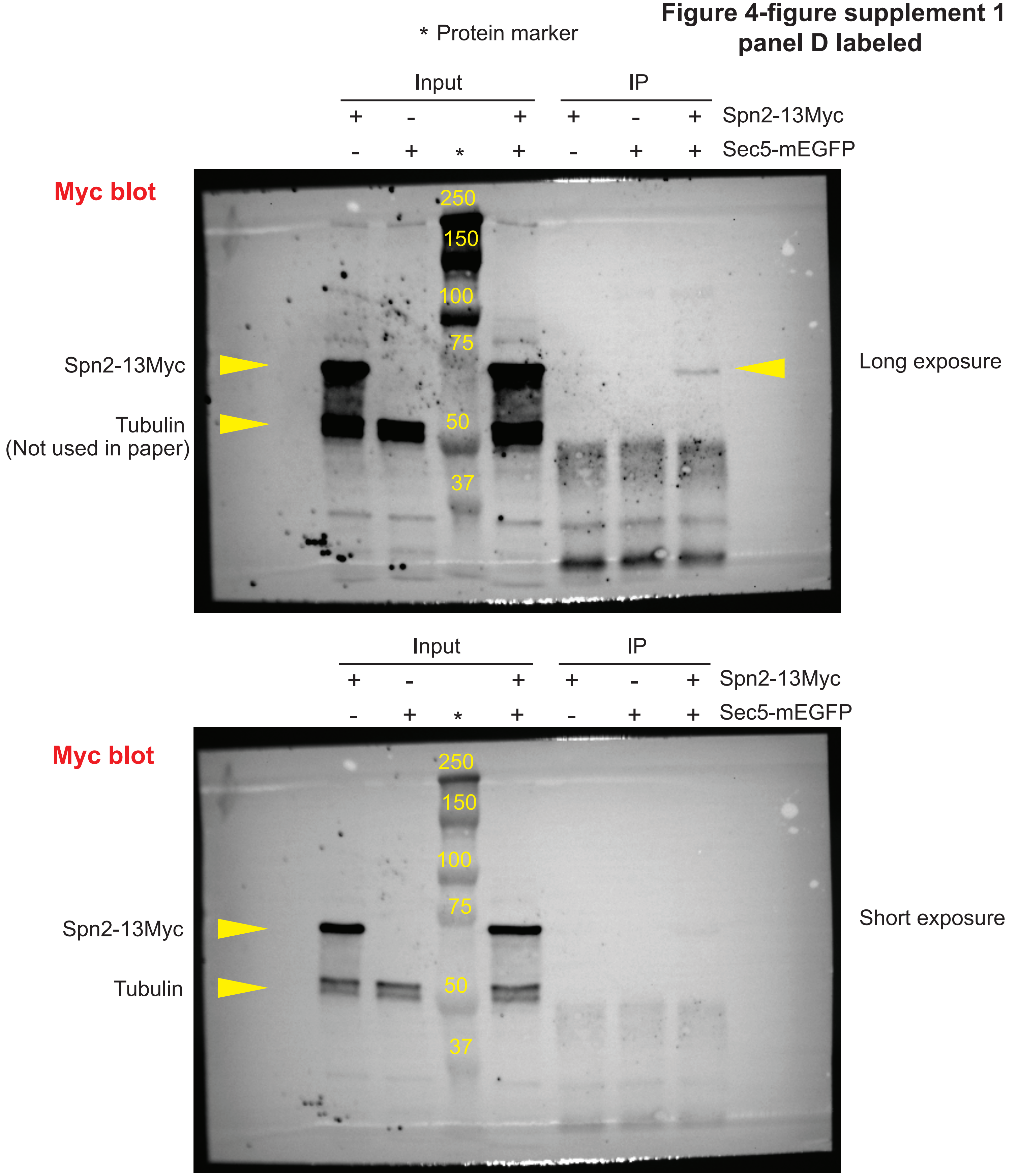

Note: White illumination near the edges of the blots are unwanted reflection of liquid from membrane and does not appear in chemiluminisence channel.

Figure 4-figure supplement 1 panel E labeled

* Protein marker

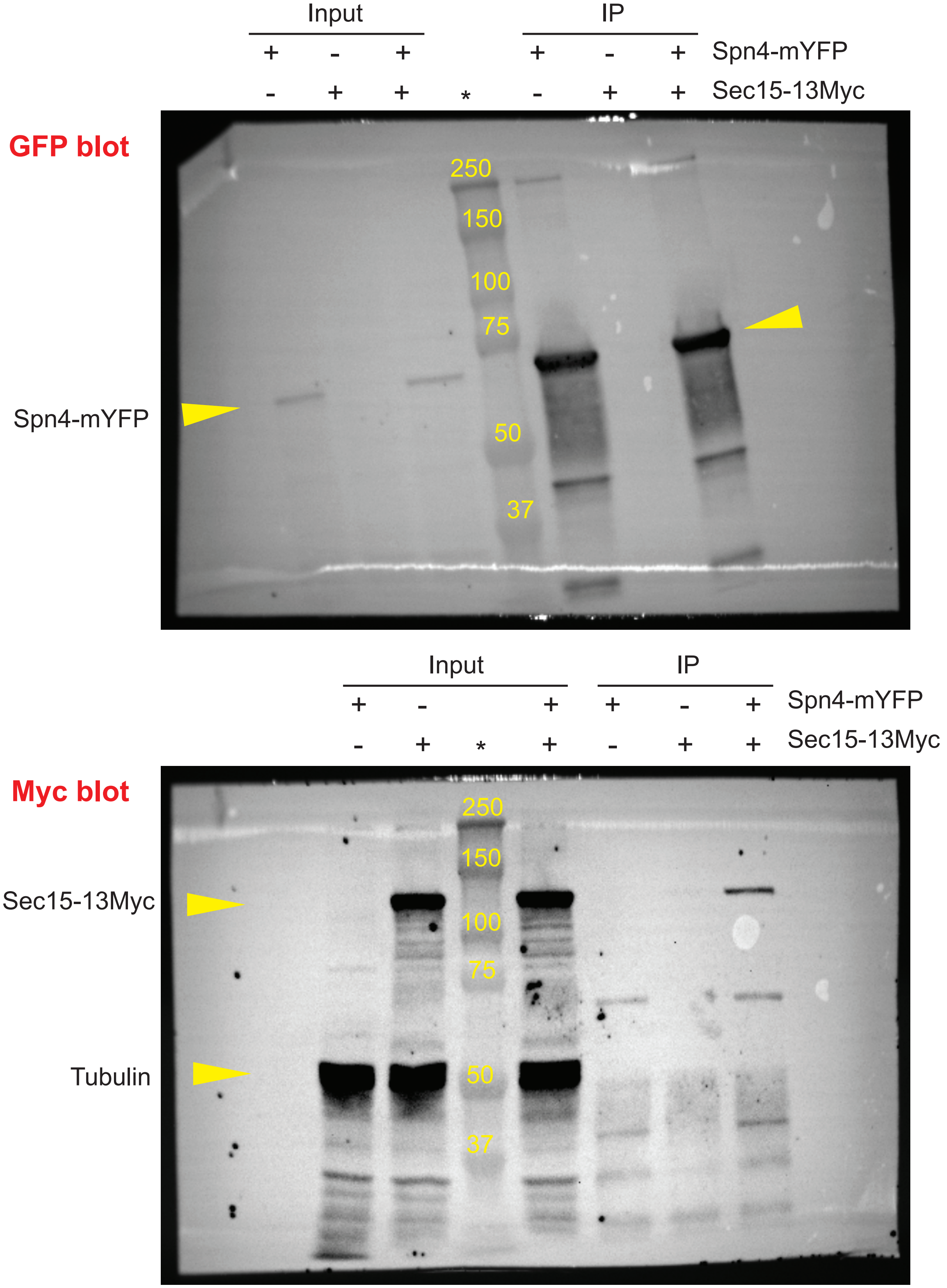

Note: White illumination near the edges of the blots are unwanted reflection of liquid from membrane and does not appear in chemiluminisence channel.

Note: Contrast was adjusted to see tubulin bands clearly from same Myc blot for the original figure in paper.

Figure 4-figure supplement 1 panel F labeled

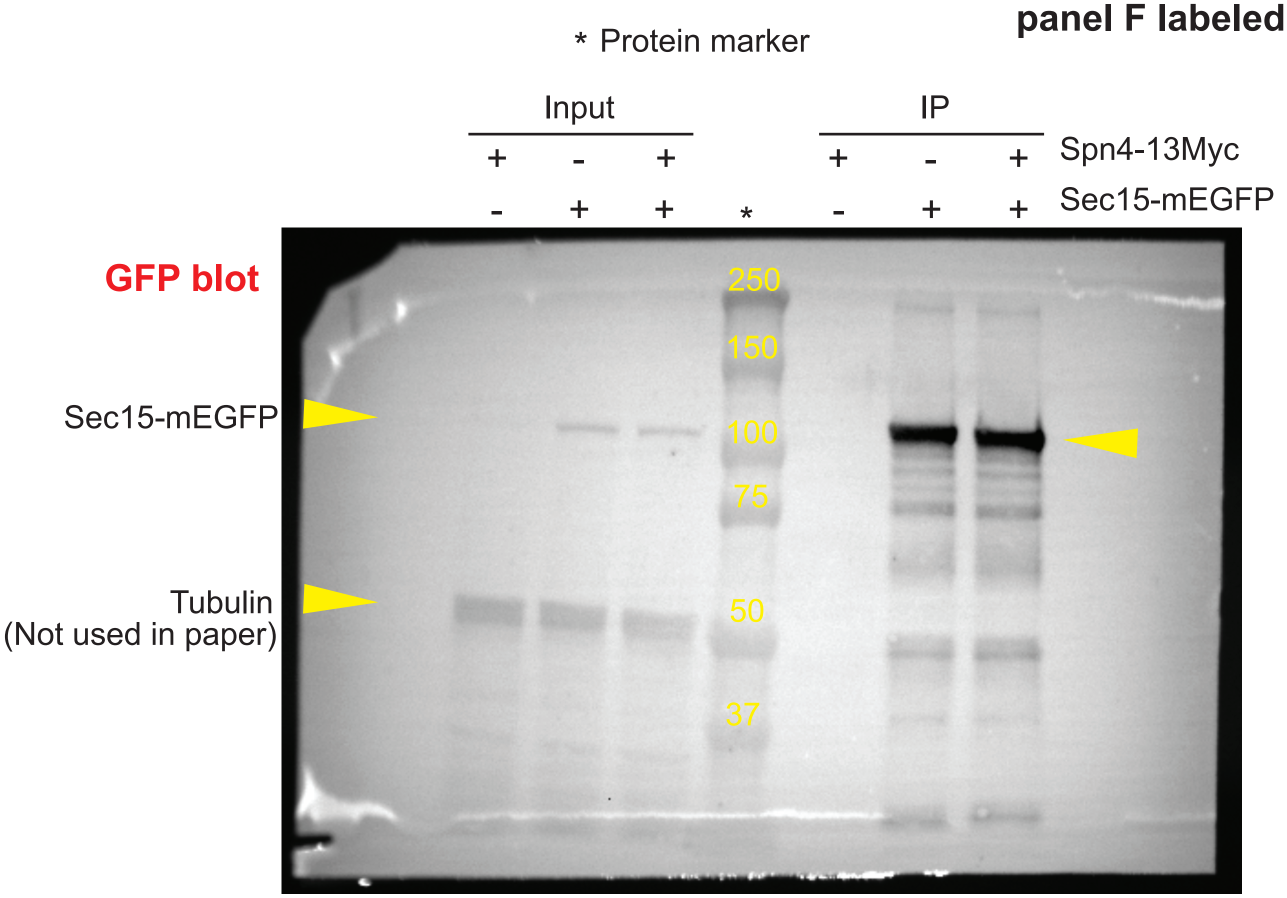

Note: White illumination near the edges of the blots are unwanted reflection of liquid from membrane and does not appear in chemiluminisence channel.

Figure 4-figure supplement 1 panel F labeled

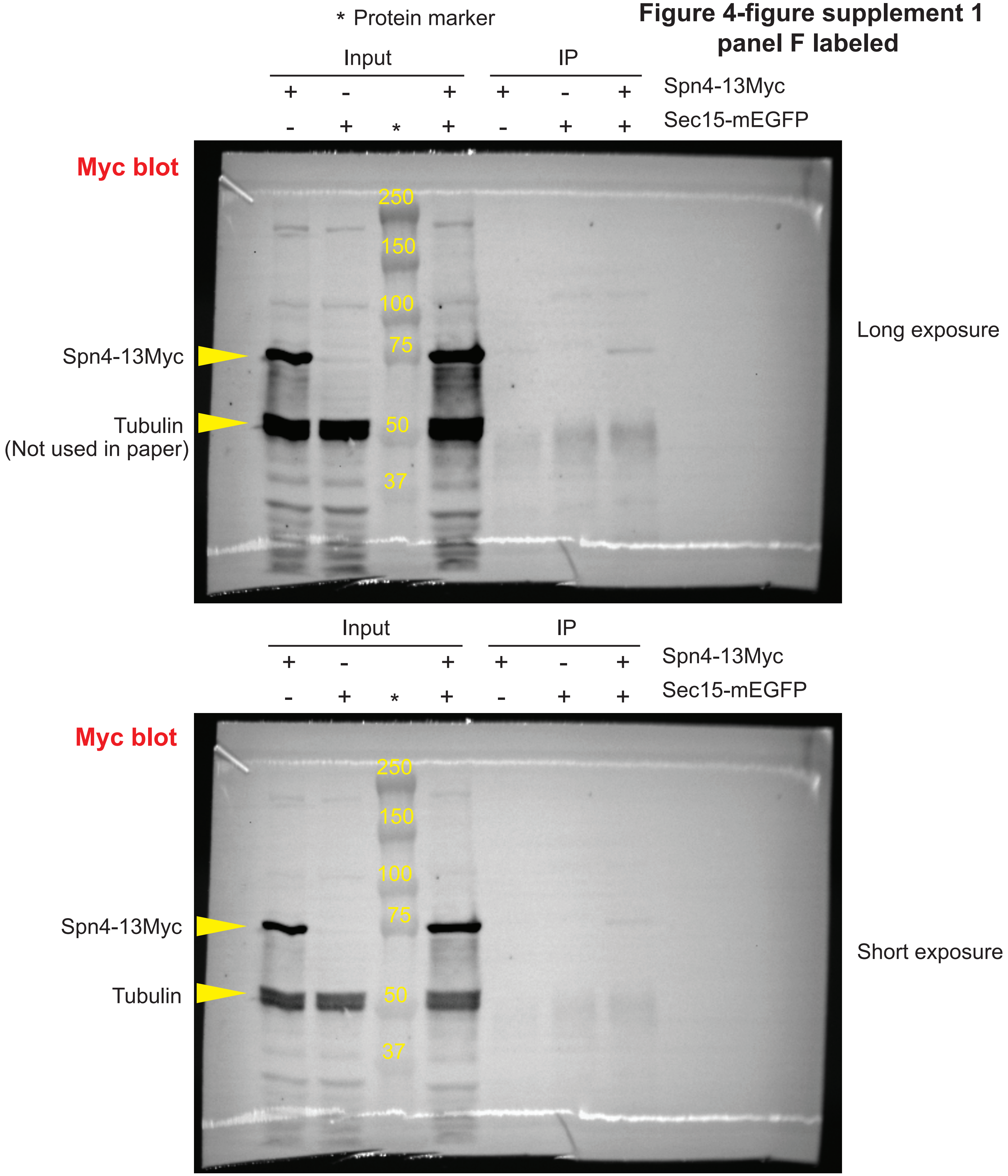

Note: White illumination near the edges of the blots are unwanted reflection of liquid from membrane and does not appear in chemiluminisence channel.

Figure 4-figure supplement 1 panel G labeled

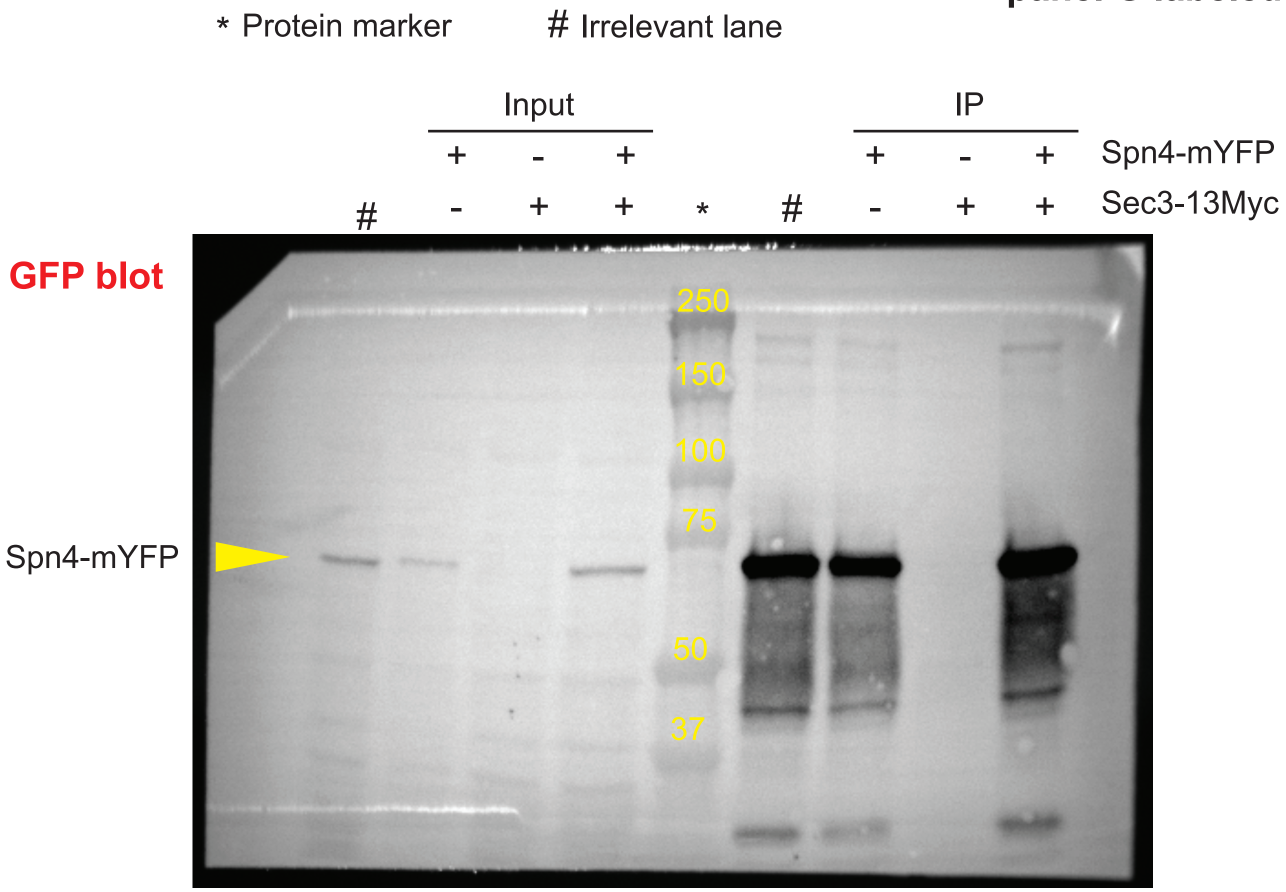

Note: White illumination near the edges of the blots are unwanted reflection of liquid from membrane and does not appear in chemiluminisence channel.

Figure 4-figure supplement 1 panel G labeled

* Protein marker # Irrelevant lane

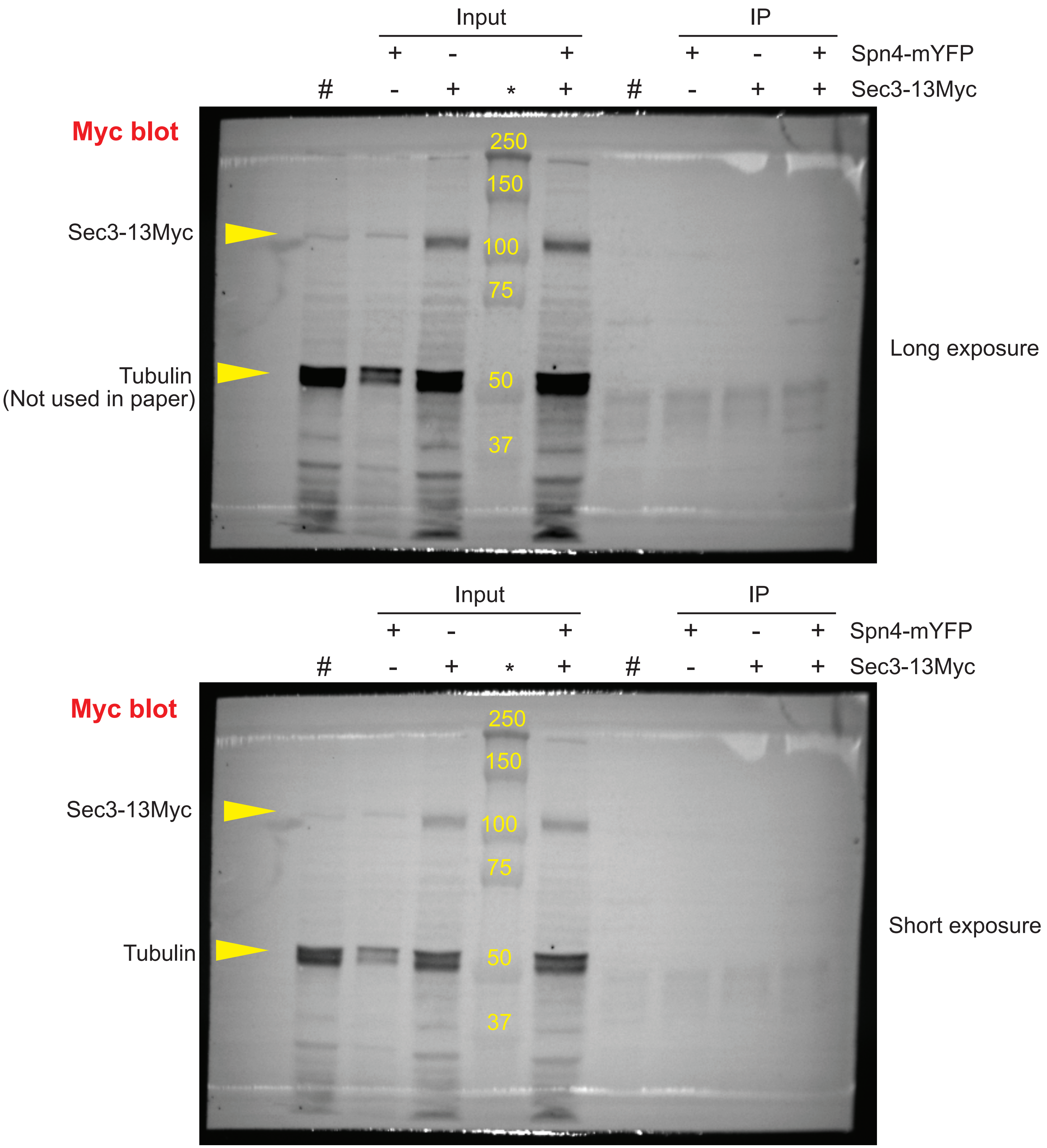

Note: White illumination near the edges of the blots are unwanted reflection of liquid from membrane and does not appear in chemiluminisence channel.

Figure 4-figure supplement 1 panel H labeled

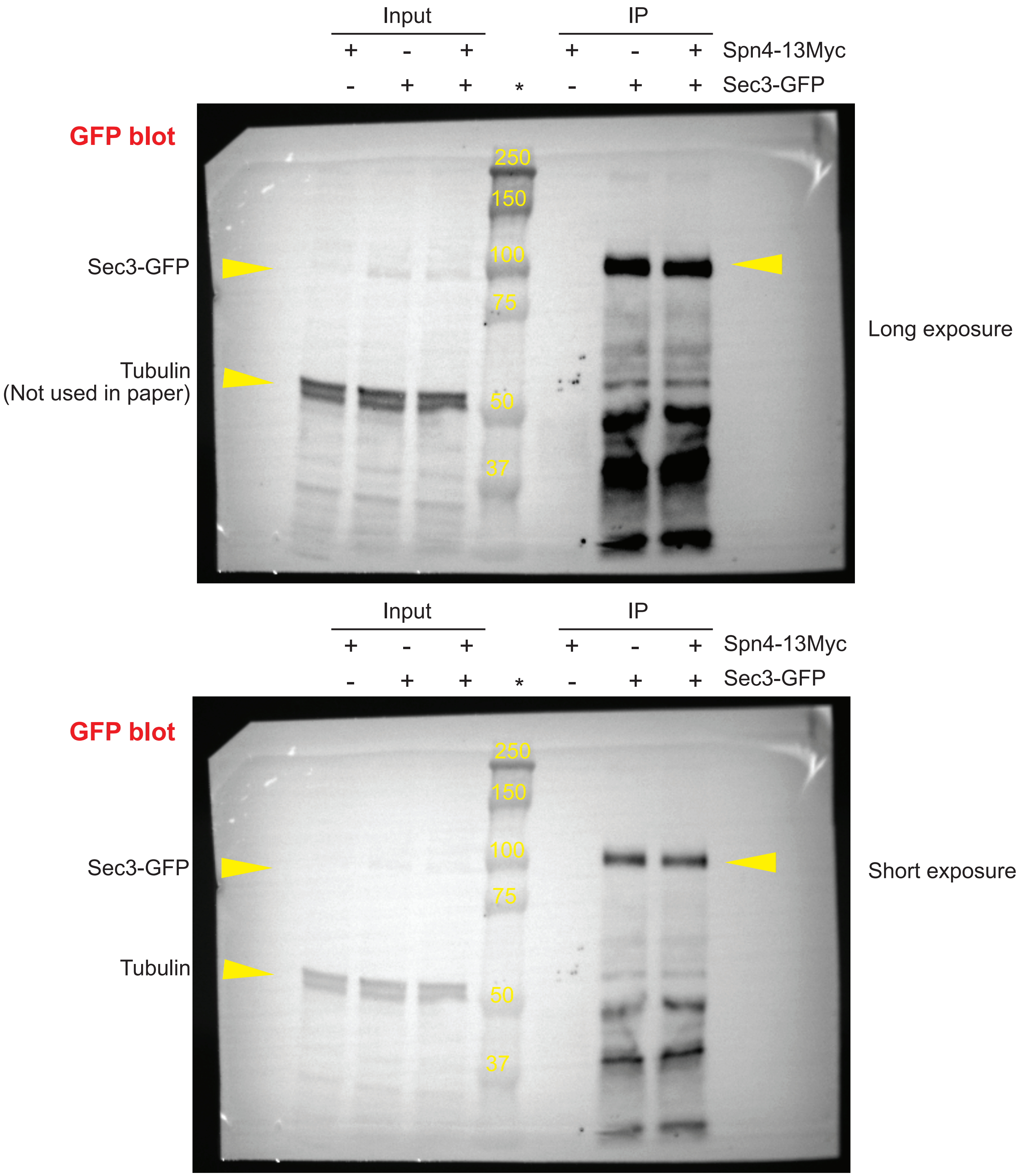

Note: White illumination near the edges of the blots are unwanted reflection of liquid from membrane and does not appear in chemiluminisence channel.

Figure 4-figure supplement 1 panel H labeled

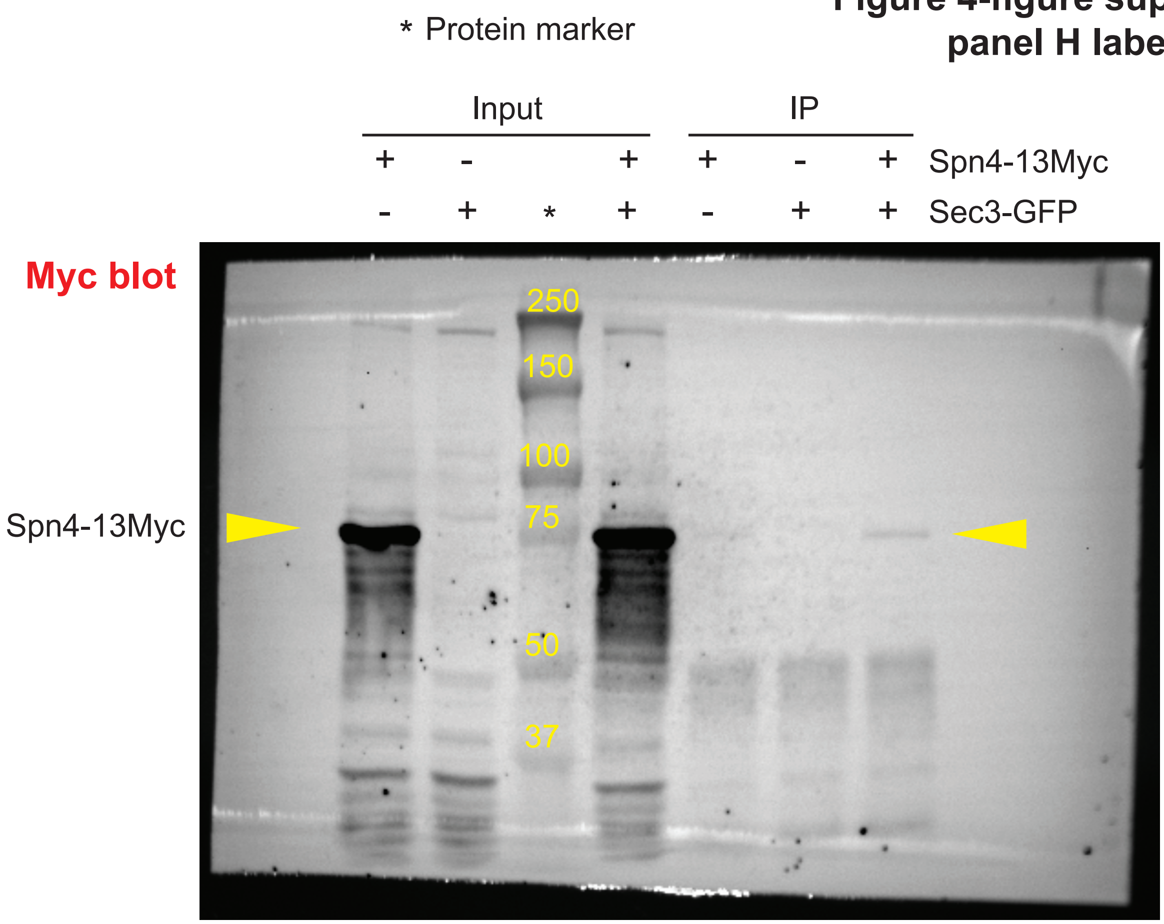

Note: White illumination near the edges of the blots are unwanted reflection of liquid from membrane and does not appear in chemiluminisence channel.
