# Supplementary material for: Septins function in exocytosis via physical interactions with the exocyst complex in fission yeast cytokinesis
Source: eLife. 2025 Oct 31;13:RP101113. doi: 10.7554/eLife.101113 (PMC12578440; doi:10.7554/eLife.101113)
Supplement: Supplementary file 1. [file elife-101113-supp1.docx]

**Supplementary file 1. *S. pombe* strains used in this study.**

| **Strain** | **Genotype** | **Figure; Video; Table; (Reference)** |
| --- | --- | --- |
| **Figure 1** | | |
| JW8692 | *sec3-tdTomato-hphMX6 spn1-mEGFP-kanMX6 sad1-mRFP1-kanMX6 ade6-M210 leu1-32 ura4-D18* | A, B, D and E |
| JW9170 | *spn1-mEGFP-kanMX6 exo70-tdTomato-natMX6 ade6-M210 leu1-32 ura4-D18* | C |
| JW1113 | *h^-^ spn1-mEGFP-kanMX6 sad1-mRFP1-kanMX6 ade6-M210 leu1-32 ura4-D18* | F |
| JW1100 | *spn1-mEGFP-kanMX6 ade6-M210 leu1-32 ura4-D18* | H and I |
| JW8928 | *spn1-mEGFP-kanMX6 sec3-913-hphMX6 ade6 leu1-32 ura4-D18* | F, H and I |
| JW8848 | *sec8-1 spn1-mEGFP-kanMX6 sad1-mRFP1-kanMX6 rng8-tdTomato-kanMX6 ade6-M210 leu1-32 ura4-D18* | G |
| **Figure 2** | | |
| IJ612 | *h^+^* *sec3-GFP-kanMX6 ade6-M216 leu1-32 ura4-D18* | A-C ([Jourdain et al., 2012](#_ENREF_59)) |
| JW7322 | *spn1-∆2::kanMX6 sec3-GFP-kanMX6 ade6-M216 leu1-32 ura4-D18* | A-C |
| JW7061 | *h^-^ sec8-GFP-ura4^+^ rlc1-tdTomato-natMX6 ade6? leu1-32 ura4-D18* | D and E |
| JW8295 | *sec8-GFP-ura4^+^ rlc1-tdTomato-natMX6 spn1-∆2::kanMX6 leu1-32 ura4-D18* | D and E |
| **Figure 4** | | |
| JW9737 | *h^+^ spn2-mEGFP-kanMX6 ade6-M210 ura4-D18 leu1-32* | A |
| JW9731 | *h^-^ sec15-13Myc-kanMX6 ade6-210 ura4-D18 leu1-32* | A and C |
| JW9757 | *spn2-mEGFP-kanMX6 sec15-13Myc-kanMX6 ade6-M210 ura4-D18 leu1-32* | A |
| JW9756 | *h^+^ spn2-13Myc-kanMX6 ade6-M210 leu1-32 ura4-D18* | B |
| JW9726 | *h^-^ sec15-mEGFP-kanMX6 ade6-210 ura4-D18 leu1-32* | B and D |
| JW9771 | *spn2-13Myc-kanMX6 sec15-mEGFP-kanMX6 ade6-210 ura4-D18 leu1-32* | B |
| JW1100 | *spn1-mEGFP-kanMX6 ade6-M210 leu1-32 ura4-D18* | C |
| JW9744 | *h^+^ spn1-mEGFP-kanMX6 sec15-13Myc-kanMX6 ade6-M210 leu1-32 ura4-D18* | C |
| JW9733 | *h^-^ spn1-13Myc-hphMX6 sec15-mEGFP-kanMX6 ade6-M210 ura4-D18 leu1-32* | D |
| JW8596 | *h^+^ spn1-13Myc-hphMX6 ade6-M210 leu1-32 ura4-D18* | D |
| **Figure 5** | | |
| JW9789 | *h^+^ spn2-∆1::hphMX6 sec15-mEGFP-kanMX6 ade6-210 ura4-D18 leu1-32* | A and B |
| JW9759 | *h^+^ sec15-mEGFP-kanMX6 ade6-210 ura4-D18 leu1-32* | A and B |
| JW9852 | *sec15-mEGFP-kanMX6 spn1-Δ2::kanMX6 ade6-M210? ura4-D18 leu1-32* | A and B |
| JW9853 | *sec15-mEGFP-kanMX6 spn4-Δ2::hphMX6 ade6-M210 ura4-D18 leu1-32* | A and B |
| JW9804 | *spn2-∆1::hphMX6 sec5-mEGFP-kanMX6 ade6-210 ura4-D18 leu1-32* | C and D |
| JW9791 | *h^+^ sec5-mEGFP-kanMX6 ade6-M210 ura4-D18 leu1-32* | C and D |
| **Figure 6** | | |
| JW81 | *h^-^ ade6-210 ura4-D18 leu1-32* | A and B |
| JW289 | *h^+^ spn1-∆2::kanMX6 leu1-32 ura4-D18* | A and B |
| MBY887 | *h^+^ sec8-1 ura4-D18 leu1-32* | A and B ([Wang et al., 2002](#_ENREF_130)) |
| JW7130 | *h^-^ kanMX6-Pypt3-mEGFP-ypt3 ade6-210 leu1-32 ura4-D18* | C |
| JW7354 | *spn1-∆2::kanMX6 kanMX6-Pypt3-mEGFP-ypt3 leu1-32 ura4-D18* | C |
| JW6548 | *h^+^ GFP-syb1-kanMX6 rlc1-tdTomato-natMX6 ade6 leu1-32 ura4-D18* | D |
| JW7385 | *spn1-∆2::kanMX6 GFP-syb1-kanMX6 rlc1-tdTomato-natMX6 leu1-32 ura4-D18* | D |
| **Figure 7** | | |
| JW5249 | *GFP-bgs1-leu1^+^ bgs1Δ::ura4^+^ rlc1-tdTomato-natMX6 ade6-M210 leu1-32 ura4-D18* | A |
| JW7264 | *GFP-bgs1-leu1^+^ bgs1Δ::ura4^+^ rlc1-tdTomato-natMX6 spn1-∆2::kanMX6 ade6 ura4-D18* | A |
| JW81 | *h^-^ ade6-210 ura4-D18 leu1-32* | B |
| JW289 | *h^+^ spn1-∆2::kanMX6 leu1-32 ura4-D18* | B |
| MBY887 | *h^+^ sec8-1 ura4-D18 leu1-32* | B ([Wang et al., 2002](#_ENREF_130)) |
| PPG37.23 | *h^-^ eng1-GFP-kan^R^ leu1-32 ura4-D18* | C ([Santos et al., 2005](#_ENREF_112)) |
| JW9057 | *eng1-GFP-kan^R^ spn1-∆2::kanMX6 leu1-32 ura4-D18* | C |
| **Figure 1-figure supplement 1** | | |
| JW1113 | *h^-^ spn1-mEGFP-kanMX6 sad1-mRFP1-kanMX6 ade6-M210 leu1-32 ura4-D18* | A, C-E; |
| JW8829 | *exo70Δ::kanMX4 spn1-mEGFP-kanMX6 sad1-mRFP1-kanMX6 ade6 leu1-32 ura4-D18* | A-D |
| JW8830 | *sec8-1 spn1-mEGFP-kanMX6 sad1-mRFP1-kanMX6 ade6-M210 leu1-32 ura4-D18* | A-D |
| JW8928 | *spn1-mEGFP-kanMX6 sec3-913-hphMX6 ade6 leu1-32 ura4-D18* | A, C and E |
| **Figure 2-figure supplement 1** | | |
| JW8929 | *h^-^ exo70-mEGFP-kanMX6 ade6-M210 ura4-D18 leu1-32* | A and C |
| JW8960 | *exo70-mEGFP-kanMX6 spn1-∆2::kanMX6 ade6-M210 leu1-32 ura4-D18* | A and C |
| JW7061 | *h^-^ sec8-GFP-ura4^+^ rlc1-tdTomato-natMX6 ade6? leu1-32 ura4-D18* | B and C |
| JW8295 | *sec8-GFP-ura4^+^ rlc1-tdTomato-natMX6 spn1-∆2::kanMX6 leu1-32 ura4-D18* | B and C |
| IJ612 | *h^+^* *sec3-GFP-kanMX6 ade6-M216 leu1-32 ura4-D18* | D and F ([Jourdain et al., 2012](#_ENREF_59)) |
| JW7322 | *spn1-∆2::kanMX6 sec3-GFP-kanMX6 ade6-M216 leu1-32 ura4-D18* | D |
| JW1100 | *spn1-mEGFP-kanMX6 ade6-M210 leu1-32 ura4-D18* | E |
| JW9058 | *gef3Δ::hphMX6 rho4Δ::kanMX4 spn1-mEGFP-kanMX6 ade6 leu1-32 ura4-D18* | E |
| JW8938 | *rho4Δ::kanMX6 sec3-GFP-kanMX6 leu1-32 ura4-D18* | F |
| JW8955 | *gef3Δ::hphMX6 sec3-GFP-kanMX6 ade6-M210 leu1-32 ura4-D18* | F |
| JW8959 | *gef3Δ::hphMX6 rho4Δ::kanMX4 sec3-GFP-kanMX6 ade6? leu1-32? ura4-D18* | F |
| **Figure 4-figure supplement 1** | | |
| JW9765 | *h^-^ sec6-13Myc-kanMX6 ade6-210 ura4-D18 leu1-32* | A |
| JW1100 | *spn1-mEGFP-kanMX6 ade6-M210 leu1-32 ura4-D18* | A |
| JW9774 | *spn1-mEGFP-kanMX6 sec6-13Myc-kanMX6 ade6-M210 leu1-32 ura4-D18* | A |
| JW8596 | *h^+^ spn1-13Myc-hphMX6 ade6-M210 leu1-32 ura4-D18* | B |
| JW9766 | *h^-^ sec6-mEGFP-kanMX6 ade6-210 ura4-D18 leu1-32* | B |
| JW9832 | *spn1-13Myc-hphMX6 sec6-mEGFP-kanMX6 ade6-210 ura4-D18 leu1-32* | B |
| JW8778 | *h^-^ spn2-mEGFP-kanMX6 ade6-M210* *leu1-32 ura4-D18* | C |
| JW9755 | *h^+^ sec5-13Myc-kanMX6 ade6-M210 leu1-32 ura4-D18* | C |
| JW9772 | *sec5-13Myc-kanMX6 spn2-mEGFP-kanMX6 ade6-210 ura4-D18 leu1-32* | C |
| JW9756 | *h^+^ spn2-13Myc-kanMX6 ade6-M210 leu1-32 ura4-D18* | D |
| JW9738 | *h^-^ sec5-mEGFP-kanMX6 ade6-M210 ura4-D18 leu1-32* | D |
| JW9775 | *spn2-13Myc-kanMX6 sec5-mEGFP-kanMX6 ade6-M210 leu1-32 ura4-D18* | D |
| JW9731 | *h^-^ sec15-13Myc-kanMX6 ade6-210 ura4-D18 leu1-32* | E |
| JW1171 | *h^+^ spn4-mYFP-kanMX6 ade6-M210 leu1-32 ura4-D18* | E and G |
| JW9829 | *sec15-13Myc-kanMX6 spn4-mYFP-kanMX6 ade6-M210 ura4-D18 leu1-32* | E |
| JW9854 | *spn4-13Myc-kanMX6 sec15-mEGFP-kanMX6 ade6-210 ura4-D18 leu1-32* | F |
| JW9768 | *h^-^ spn4-13Myc-kanMX6 ade6-210 ura4-D18 leu1-32* | F and H |
| JW9759 | *h^+^ sec15-mEGFP-kanMX6 ade6-210 ura4-D18 leu1-32* | F |
| JW9139 | *h^-^ sec3-13Myc-natMX6 ade6-210 leu1-32 ura4-D18* | G |
| JW9711 | *spn4-mYFP-kanMX6 sec3-13Myc-natMX6 ade6-M210 leu1-32 ura4-D18* | G |
| JW7300 | *h^+^ sec3-GFP-kanMX6 ade6-M210 leu1-32 ura4-D18* | H |
| JW9788 | *spn4-13Myc-kanMX6 sec3-GFP-kanMX6 ade6-210 leu1-32 ura4-D18* | H |
| **Table 1 and 2** | | |
| JW7035 | *h^-^ trs120-M1-his5^+^-kanMX6 his5Δ ade6-M210 leu1-32 ura4* | Tables 1 and 2 |
| JW289 | *h^+^ spn1-∆2::kanMX6 leu1-32 ura4-D18* | Tables 1 and 2 |
| JW8821 | *trs120-M1-his5^+^-kanMX6 spn1-∆2::kanMX6 leu1-32 ura4* | Tables 1 and 2 |
| JW7036 | *h^-^ trs120-ts1-his5^+^-kanMX6 his5Δ ade6-M210 leu1-32 ura4* | Tables 1 and 2 |
| JW8822 | *trs120-ts1-his5^+^-kanMX6 spn1-∆2::kanMX6 leu1-32 ura4* | Tables 1 and 2 |
| JW290 | *h^-^ spn1-∆2::kanMX6 his3-27 ura4-D18* | Tables 1 and 2 |
| MBY887 | *h^+^ sec8-1 ura4-D18 leu1-32* | Tables 1 and 2 |
| JW8796 | *spn1-∆2::kanMX6 sec8-1 ura4-D18* | Tables 1 and 2 |
| JW2716 | *h^+^ exo70Δ::kanMX4 ade6 leu1-32 ura4-D18* | Tables 1 and 2 |
| JW8797 | *spn1-∆2::kanMX6 exo70Δ::kanMX4 his3-27 ade6 ura4-D18* | Tables 1 and 2 |
| IJ1032 | *h^–^ sec3-916-hphMX6 ade6-M216 leu1-32 ura4-D18* | Tables 1 and 2; ([Jourdain et al., 2012](#_ENREF_59)) |
| JW8787 | *spn1-∆2::kanMX6 sec3-916-hphMX6 ade6-M216 leu1-32 ura4-D18* | Tables 1 and 2 |
| IJ767 | *h^–^ sec3-913-hphMX6 ade6-M216 leu1-32 ura4-D18* | Tables 1 and 2; ([Jourdain et al., 2012](#_ENREF_59)) |
| JW8783 | *spn1-∆2::kanMX6 sec3-913-hphMX6 ade6-M216 leu1-32 ura4-D18* | Tables 1 and 2 |
| JW8588 | *spn2-∆1::ura4^+^ ade6 leu1-32 ura4-D18* | Tables 1 and 2 |
| JW8782 | *spn2-∆1::ura4^+^ sec3-913-hphMX6 ade6 leu1-32 ura4-D18* | Tables 1 and 2 |
| JW8789 | *spn2-∆1::ura4^+^ sec3-916-hphMX6 ade6 leu1-32 ura4-D18* | Tables 1 and 2 |
| JW8590 | *h^+^ spn3-Δ2::kanMX6 ade6-M210 leu1-32 ura4-D18* | Tables 1 and 2 |
| JW8790 | *spn3-Δ2::kanMX6 sec3-913-hphMX6 ade6-M21X leu1-32 ura4-D18* | Tables 1 and 2 |
| JW8785 | *spn3-Δ2::kanMX6 sec3-916-hphMX6 ade6-M21X leu1-32 ura4-D18* | Tables 1 and 2 |
| JW293 | *h^-^ spn4-∆2::kanMX6 ura4-D18* | Tables 1 and 2 |
| JW295 | *h^+^ spn4-∆2::kanMX6 leu1-32 ura4-D18* | Tables 1 and 2 |
| JW8784 | *spn4-∆2::kanMX6 sec3-913-hphMX6 ade6-M216 leu1-32 ura4-D18* | Tables 1 and 2 |
| JW8788 | *spn4-∆2::kanMX6 sec3-916-hphMX6 leu1-32 ura4-D18* | Tables 1 and 2 |
| JW8799 | *spn4-∆2::kanMX6 sec8-1 leu1-32 ura4-D18* | Tables 1 and 2 |
| **Videos** | | |
| JW9170 | *spn1-mEGFP-kanMX6 exo70-tdTomato-natMX6 ade6-M210 leu1-32 ura4-D18* | Figure 1-videos 1-3 |
| JW9726 | *h^-^ sec15-mEGFP-kanMX6 ade6-210 ura4-D18 leu1-32* | Figure 5-video 1 |
| JW9852 | *sec15-mEGFP-kanMX6 spn1-Δ2::kanMX6 ade6-M210? ura4-D18 leu1-32* | Figure 5-video 2 |
| JW9853 | *sec15-mEGFP-kanMX6 spn4-Δ2::hphMX6 ade6-M210 ura4-D18 leu1-32* | Figure 5-video 3 |
